# Supplementary material for: scBoolSeq: Linking scRNA-seq statistics and Boolean dynamics
Source: PLoS Comput Biol. 2024 Jul 8;20(7):e1011620. doi: 10.1371/journal.pcbi.1011620 (PMC11257695; doi:10.1371/journal.pcbi.1011620)
Supplement: S1 Notebooks — The notebooks are provided as static HTML files, and Boolean networks as textual files in BoolNet format. See the Data availability statement for links to executable notebooks and code. (ZIP) [file pcbi.1011620.s001.zip › Case_Study_Early-born_Retinal_Neurons/Aggregation_comparison.html]

Aggregation\_comparison


# Aggregation Method Comparison¶

In [1]:

```
from IPython.display import display, HTML
display(HTML("<style>.container { width:95% !important; }</style>"))
import warnings
warnings.filterwarnings("ignore") # umap deprecation warnings related to numba do not concern us.
```

In [2]:

```
from pathlib import Path as path
import functools as fn
import itertools as it
import json

import numpy as np
import pandas as pd
import networkx as nx
import matplotlib.pyplot as plt
from matplotlib_venn import venn3, venn3_circles
from sklearn.utils import Bunch

from scboolseq.utils import parse_data_directory, parse_pickles
from scboolseq import scBoolSeq

from markers import (
    positive_markers, # function to generate a configuration dictionnary from a set of marker genes
    states_and_markers, # Dictionnary containing phenotypes and their marker genes
    marker_genes, # pandas.Series containing simply the name of markers
)
```

In [3]:

```
%ls *_analysis/*_grn_optimized_v1.json
```

```
bootstrap_analysis/bootstrap_grn_optimized_v1.json
mode_analysis/mode_grn_optimized_v1.json
sum_analysis/sum_grn_optimized_v1.json
```

In [5]:

```
here = path(".").resolve()
```

In [6]:

```
grns = Bunch()
for f in here.glob("*_analysis/*_grn_optimized_v1.json"):
    with open(f.as_posix(), "r") as _js:
        grns.update({f.name.split("_")[0]: nx.node_link_graph(json.load(_js))})
```

In [7]:

```
grns.keys()
```

Out[7]:

```
dict_keys(['sum', 'mode', 'bootstrap'])
```

In [8]:

```
kept_nodes = Bunch(**{k: set(v.nodes) for k, v in grns.items()})
```

In [9]:

```
venn3(
    [kept_nodes.sum, kept_nodes.mode, kept_nodes.bootstrap],
    ("Sum", "Mode", "Bootstrap")
)
```

Out[9]:

```
<matplotlib_venn._common.VennDiagram at 0x7f25ef9d9810>
```

In [10]:

```
{k: len(v) for k, v in kept_nodes.items()}
```

Out[10]:

```
{'sum': 184, 'mode': 243, 'bootstrap': 177}
```

In [18]:

```
for _method, _nodes in kept_nodes.items():
    with open(f"{_method}_kept_nodes.txt", "w") as f:
        f.write("Gene\n")
        for n in _nodes:
            f.write(f"{n}\n")
```

In [23]:

```
list(it.combinations(kept_nodes, 2))
```

Out[23]:

```
[('sum', 'mode'), ('sum', 'bootstrap'), ('mode', 'bootstrap')]
```

In [24]:

```
intersection_all = kept_nodes.mode & kept_nodes.sum & kept_nodes.bootstrap
```

In [ ]:

```

```
