# Supplementary material for: scBoolSeq: Linking scRNA-seq statistics and Boolean dynamics
Source: PLoS Comput Biol. 2024 Jul 8;20(7):e1011620. doi: 10.1371/journal.pcbi.1011620 (PMC11257695; doi:10.1371/journal.pcbi.1011620)
Supplement: S1 Notebooks — The notebooks are provided as static HTML files, and Boolean networks as textual files in BoolNet format. See the Data availability statement for links to executable notebooks and code. (ZIP) [file pcbi.1011620.s001.zip › Case_Study_Early-born_Retinal_Neurons/0.- Highly Variable Gene Selection.html]

0.- Highly Variable Gene Selection


# Highly Variable Gene Selection¶

Refs:

- Quentin Lo Giudice, Marion Leleu, Gioele La Manno, and Pierre J. Fabre. Single-cell transcriptional logic of cell-fate specification and axon guidance in early-born retinal neurons. Development (Cambridge), 146(17), 2019.
- Peter V. Kharchenko. The triumphs and limitations of computational methods for scRNA-seq. Nature Methods, 18(7):723–732, 2021

GEO Accession GSE122466

In [1]:

```
from IPython.display import display, HTML
display(HTML("<style>.container { width:95% !important; }</style>"))
```

In [2]:

```
from pathlib import Path as path
import warnings
warnings.filterwarnings("ignore") # anndata deprecation warnings related to pandas and numba do not concern us.
```

In [3]:

```
import stream as st
st.__version__
```

Out[3]:

```
'1.0'
```

In [4]:

```
import itertools as it
from markers import (
    positive_markers, # function to generate a configuration dictionnary from a set of marker genes
    states_and_markers, # Dictionnary containing phenotypes and their marker genes
)
```

In [5]:

```
st.set_figure_params(
    dpi=120,
    style='white',
    figsize=[5.4,4.8],
    rc={'image.cmap': 'viridis'}
)
```

In [6]:

```
!test -f GSE122466_Retina_Batch1_RAW.tsv || echo "Select observarions whose ID starts with Lane1_"
```

In [7]:

```
_in_dir = path(".").resolve()
_publish_dir = path(".").resolve()
if not _publish_dir.exists():
    _publish_dir.mkdir()
print(f"Reading data from: {_in_dir}")
print(f"Saving processed data to: {_publish_dir}")
```

```
Reading data from: /data
Saving processed data to: /data
```

In [8]:

```
infile = _in_dir /'GSE122466_Retina_Batch1_RAW.tsv'
infile
```

Out[8]:

```
PosixPath('/data/GSE122466_Retina_Batch1_RAW.tsv')
```

In [9]:

```
outfile = _publish_dir / "GSE122466_Retina_vargenes_batch1.csv"
outfile
```

Out[9]:

```
PosixPath('/data/GSE122466_Retina_vargenes_batch1.csv')
```

#### Read in data¶

In [10]:

```
%%time 
adata = st.read(file_name=infile.as_posix(), workdir='./stream_result')
```

```
Saving results in: ./stream_result
CPU times: user 3.76 s, sys: 60.8 ms, total: 3.82 s
Wall time: 3.82 s
```

In [11]:

```
adata.obs_names_make_unique()
adata.var_names_make_unique()
```

In [12]:

```
adata
```

Out[12]:

```
AnnData object with n_obs × n_vars = 2673 × 15176 
    uns: 'workdir'
```

In [13]:

```
import pandas as pd
```

#### Calculate QC¶

In [14]:

```
st.cal_qc(adata,assay='rna')
```

In [15]:

```
st.plot_qc(adata,jitter=0.3,)
```

In [16]:

```
### histogram plots and log-scale are also supported
st.plot_qc(adata,jitter=0.3,log_scale=[0,1,4,5],hist_plot=[0,1,4,5])
```

In [17]:

```
# From the article
st.filter_cells(adata, min_n_features=200)
st.filter_features(adata, min_n_cells =3)
```

```
filter cells based on min_n_features
after filtering out low-quality cells: 
2673 cells, 15176 genes
Filter genes based on min_n_cells
After filtering out low-expressed genes: 
2673 cells, 14518 genes
```

In [18]:

```
###Normalize gene expression based on library size  
st.normalize(adata,method='lib_size')
###Logarithmize gene expression 
st.log_transform(adata)
###Remove mitochondrial genes 
st.remove_mt_genes(adata)
```

```
remove mitochondrial genes:
['mt-Nd1', 'mt-Nd2', 'mt-Co1', 'mt-Co2', 'mt-Atp8', 'mt-Atp6', 'mt-Co3', 'mt-Nd3', 'mt-Nd4l', 'mt-Nd4', 'mt-Nd5', 'mt-Nd6', 'mt-Cytb']
```

#### Feature selection¶

Please check if the blue curve fits the points well. If not, please adjust the parameter **'loess\_frac'** (usually by lowering it) until the blue curve fits well.

In [19]:

```
%%time
st.select_variable_genes(adata,loess_frac=0.01,n_genes=1648) 
# The number of variable genes is set to 1_648 because it was the number of genes kept in the original article's analyses
```

```
1648 variable genes are selected
CPU times: user 3.02 s, sys: 715 ms, total: 3.74 s
Wall time: 5.35 s
```

In [20]:

```
marker_genes = pd.Series(list(set(it.chain.from_iterable(states_and_markers.values()))))
len(marker_genes)
```

Out[20]:

```
22
```

In [21]:

```
100 * marker_genes.isin(adata.var_names).mean()
```

Out[21]:

```
100.0
```

In [22]:

```
100 * marker_genes.isin(adata.uns["var_genes"]).mean()
```

Out[22]:

```
90.9090909090909
```

Not all marker genes were selected as highly variable, but they can be manually added:

In [23]:

```
marker_genes[~marker_genes.isin(adata.uns["var_genes"])]
```

Out[23]:

```
11    Pou4f2
19      Rbp4
dtype: object
```

In [33]:

```
export_markers = adata.uns['var_genes'].copy(deep=True)
export_markers = export_markers.append(pd.Index(marker_genes[~marker_genes.isin(adata.uns["var_genes"])]))
export_markers
```

Out[33]:

```
Index(['Tubb3', 'Malat1', 'Stmn2', 'Fgf15', 'Gap43', 'Xist', 'Sncg', 'Hmgb2',
       'Top2a', 'Meg3',
       ...
       'Prdm13', 'Kif14', 'Rpl24', 'Etfb', 'Cd320', 'Fam98b', 'Odf2', 'Fbxo36',
       'Rbp4', 'Pou4f2'],
      dtype='object', length=1650)
```

#### Save most variable genes to a new dataframe¶

In [34]:

```
# extract the genes index
var_genes = adata.uns["var_genes"]
var_genes
```

Out[34]:

```
Index(['Tubb3', 'Malat1', 'Stmn2', 'Fgf15', 'Gap43', 'Xist', 'Sncg', 'Hmgb2',
       'Top2a', 'Meg3',
       ...
       'Depdc1a', 'Agpat1', 'Prdm13', 'Kif14', 'Rpl24', 'Etfb', 'Cd320',
       'Fam98b', 'Odf2', 'Fbxo36'],
      dtype='object', length=1648)
```

In [35]:

```
list(_publish_dir.glob("*csv"))
```

Out[35]:

```
[]
```

In [36]:

```
adata.to_df()[export_markers].to_csv(outfile.as_posix())
```

In [37]:

```
list(_publish_dir.glob("*csv"))
```

Out[37]:

```
[PosixPath('/data/GSE122466_Retina_vargenes_batch1.csv')]
```

In [ ]:

```

```
