# Supplementary material for: scBoolSeq: Linking scRNA-seq statistics and Boolean dynamics
Source: PLoS Comput Biol. 2024 Jul 8;20(7):e1011620. doi: 10.1371/journal.pcbi.1011620 (PMC11257695; doi:10.1371/journal.pcbi.1011620)
Supplement: S1 Notebooks — The notebooks are provided as static HTML files, and Boolean networks as textual files in BoolNet format. See the Data availability statement for links to executable notebooks and code. (ZIP) [file pcbi.1011620.s001.zip › Case_Study_Early-born_Retinal_Neurons/2.- Trajectory Inference (STREAM).html]

2.- Trajectory Inference (STREAM)


Refs:

- Quentin Lo Giudice, Marion Leleu, Gioele La Manno, and Pierre J. Fabre. Single-cell transcriptional logic of cell-fate specification and axon guidance in early-born retinal neurons. Development (Cambridge), 146(17), 2019.
- Peter V. Kharchenko. The triumphs and limitations of computational methods for scRNA-seq. Nature Methods, 18(7):723–732, 2021

GEO Accession GSE122466

In [4]:

```
from IPython.display import display, HTML
display(HTML("<style>.container { width:95% !important; }</style>"))
```

In [5]:

```
from pathlib import Path as path
import itertools as it
import warnings
warnings.filterwarnings("ignore") # anndata deprecation warnings related to pandas and numba do not concern us.
```

In [6]:

```
import stream as st
st.__version__
```

Out[6]:

```
'1.0'
```

In [7]:

```
import itertools as it
from markers import (
    positive_markers, # function to generate a configuration dictionnary from a set of marker genes
    states_and_markers, # Dictionnary containing phenotypes and their marker genes
)
```

In [8]:

```
st.set_figure_params(
    dpi=120,
    style='white',
    figsize=[5.4,4.8],
    rc={'image.cmap': 'viridis'}
)
```

In [1]:

```
%ls *tsv
```

```
GSE122466_metadata_batch1_v3.tsv  GSE122466_Retina_Batch1_RAW.tsv
```

In [10]:

```
!test -f GSE122466_Retina_Batch1_RAW.tsv || echo "Select observarions whose ID starts with Lane1_"
```

In [11]:

```
_in_dir = path(".").resolve()
_publish_dir = path(".").resolve()
if not _publish_dir.exists():
    _publish_dir.mkdir()
print(f"Reading data from: {_in_dir}")
print(f"Saving processed data to: {_publish_dir}")
```

```
Reading data from: /data
Saving processed data to: /data
```

In [12]:

```
infile = _in_dir /'GSE122466_Retina_Batch1_RAW.tsv'
infile
```

Out[12]:

```
PosixPath('/data/GSE122466_Retina_Batch1_RAW.tsv')
```

#### Read in data¶

In [13]:

```
%%time 
adata = st.read(file_name=infile.as_posix(), workdir='./stream_result')
```

```
Saving results in: ./stream_result
CPU times: user 3.87 s, sys: 94 ms, total: 3.97 s
Wall time: 3.96 s
```

> **To load and use 10x Genomics single cell RNA-seq data processed with Cell Ranger:**  
> (*The variable index can be reset by choosing a different column in `gene.tsv`*)

```
adata=st.read(file_name='./filtered_gene_bc_matrices/matrix.mtx',
              file_feature='./filtered_gene_bc_matrices/genes.tsv',
              file_sample='./filtered_gene_bc_matrices/barcodes.tsv',
              file_format='mtx',workdir='./stream_result')    
adata.var.index = adata.var[1].values
```

> **If the Anndata object is already created, to run STREAM, please simply specify work directory:**

```
st.set_workdir(adata,'./stream_result')
```

In [14]:

```
adata.obs_names_make_unique()
adata.var_names_make_unique()
```

In [15]:

```
adata
```

Out[15]:

```
AnnData object with n_obs × n_vars = 2673 × 15176 
    uns: 'workdir'
```

In [16]:

```
st.add_metadata(
    adata, 
    file_name='GSE122466_metadata_batch1_v3.tsv'
)
adata.obs.head()
```

Out[16]:

|  | label | label\_color | louvain | discard |
| --- | --- | --- | --- | --- |
| Lane1\_AAACCTGAGATGTCGG | RPC | #66c2a5 | 0 | False |
| Lane1\_AAACCTGCAATCCAAC | RPC | #66c2a5 | 0 | False |
| Lane1\_AAACCTGGTTCCTCCA | RGC | #fc8d62 | 6 | False |
| Lane1\_AAACCTGTCCAATGGT | AC | #8da0cb | 2 | False |
| Lane1\_AAACGGGAGGCAATTA | RPC | #66c2a5 | 1 | False |

In [17]:

```
import pandas as pd
import matplotlib.pyplot as plt
```

#### Plot QC¶

This is the same as shown in notebook `0.- Highly Variable Gene Selection.ipynb`. It is not executed to save space but the code is the following:

```
st.plot_qc(adata,jitter=0.3,)
st.plot_qc(adata,jitter=0.3,log_scale=[0,1,4,5],hist_plot=[0,1,4,5])
```

In [18]:

```
st.cal_qc(adata,assay='rna')
```

In [19]:

```
# filtering criteria taken from the article:
st.filter_cells(adata, min_n_features=200)
st.filter_features(adata, min_n_cells =3)
```

```
filter cells based on min_n_features
after filtering out low-quality cells: 
2673 cells, 15176 genes
Filter genes based on min_n_cells
After filtering out low-expressed genes: 
2673 cells, 14518 genes
```

In [20]:

```
###Normalize gene expression based on library size  
st.normalize(adata,method='lib_size')
###Logarithmize gene expression 
st.log_transform(adata)
###Remove mitochondrial genes 
st.remove_mt_genes(adata)
```

```
remove mitochondrial genes:
['mt-Nd1', 'mt-Nd2', 'mt-Co1', 'mt-Co2', 'mt-Atp8', 'mt-Atp6', 'mt-Co3', 'mt-Nd3', 'mt-Nd4l', 'mt-Nd4', 'mt-Nd5', 'mt-Nd6', 'mt-Cytb']
```

#### Feature selection¶

Please check if the blue curve fits the points well. If not, please adjust the parameter **'loess\_frac'** (usually by lowering it) until the blue curve fits well.

In [21]:

```
%%time
st.select_variable_genes(adata,loess_frac=0.01,n_genes=1648)
# ^ The number of genes is set to retain the same number of variable genes selected in the original work.
```

```
1648 variable genes are selected
CPU times: user 2.88 s, sys: 1.08 s, total: 3.96 s
Wall time: 5.11 s
```

In [22]:

```
marker_genes = pd.Series(list(set(it.chain.from_iterable(states_and_markers.values()))))
```

In [23]:

```
if (~marker_genes.isin(adata.uns["var_genes"])).any():
    print("Appending missing markers")
    adata.uns['var_genes'] = adata.uns['var_genes'].append(
        pd.Index(marker_genes[~marker_genes.isin(adata.uns["var_genes"])])
    )
```

```
Appending missing markers
```

In [24]:

```
st.select_top_principal_components(adata, n_pc=25, first_pc=True, feature="var_genes")
```

```
using top variable genes ...
25 PCs are selected
```

- 'se': Spectral embedding algorithm
- 'mlle': Modified locally linear embedding algorithm
- 'umap': Uniform Manifold Approximation and Projection
- 'pca': Principal component analysis

In [25]:

```
adata
```

Out[25]:

```
AnnData object with n_obs × n_vars = 2673 × 14505 
    obs: 'label', 'label_color', 'louvain', 'discard', 'n_counts', 'n_genes', 'pct_genes', 'pct_mt'
    var: 'n_counts', 'n_cells', 'pct_cells'
    uns: 'workdir', 'label_color', 'assay', 'var_genes', 'pca_variance_ratio', 'top_pcs', 'params'
    obsm: 'var_genes', 'pca', 'top_pcs'
```

In [27]:

```
# See notebook `1.- Retinal Differentiation Clustering and Metadata.ipynb`
if adata.obs[adata.obs['discard']].index.isin(adata.obs.index).any():
    print("Removing outlier group (poorly mapped to known phenotypes)")
    adata = adata[~adata.obs['discard'], :]
else:
    print("Dataset is ready for trajectory inference")
```

```
Dataset is ready for trajectory inference
```

In [28]:

```
st.dimension_reduction(
    adata, method='umap',
    feature='top_pcs', n_components=3,
    n_neighbors=15, n_jobs=12
)
st.plot_dimension_reduction(adata, color=["label", "n_genes", *states_and_markers['Cones']], n_components=2, show_graph=False, show_text=False)
```

```
feature top_pcs is being used ...
12 cpus are being used ...
```

```
Trying to set attribute `.uns` of view, making a copy.
```

In [31]:

```
st.seed_elastic_principal_graph(adata,n_clusters=10)
```

```
Seeding initial elastic principal graph...
Clustering...
K-Means clustering ...
The number of initial nodes is 10
Calculatng minimum spanning tree...
Number of initial branches: 4
```

In [32]:

```
st.plot_dimension_reduction(adata,color=['label', *states_and_markers['NB1']],n_components=2,show_graph=True,show_text=False)
```

In [33]:

```
st.elastic_principal_graph(adata,epg_alpha=0.01,epg_mu=0.07,epg_lambda=0.05)
```

```
Learning elastic principal graph...
[1]
 "Constructing tree 1 of 1 / Subset 1 of 1"


[1]
 "Computing EPG with 50 nodes on 2559 points and 3 dimensions"


[1]
 "Using a single core"


Nodes = 
10
 
11
 
12
 
13
 
14
 
15
 
16
 
17
 
18
 
19
 
20
 
21
 
22
 
23
 
24
 
25
 
26
 
27
 
28
 
29
 
30
 
31
 
32
 
33
 
34
 
35
 
36
 
37
 
38
 
39
 
40
 
41
 
42
 
43
 
44
 
45
 
46
 
47
 
48
 
49
 


BARCODE	ENERGY	NNODES	NEDGES	NRIBS	NSTARS	NRAYS	NRAYS2	MSE	MSEP	FVE	FVEP	UE	UR	URN	URN2	URSD

1|1||50
	
1.449
	
50
	
49
	
43
	
1
	
0
	
0
	
0.5551
	
0.5358
	
0.9851
	
0.9856
	
0.8476
	
0.0467
	
2.335
	
116.8
	
0


17.57 sec elapsed

[[1]]


Number of branches after learning elastic principal graph: 6
```

In [35]:

```
st.plot_dimension_reduction(adata,color=['label'], n_components=3,show_graph=True,show_text=False, plotly=True)
```

In [36]:

```
st.prune_elastic_principal_graph(adata,epg_collapse_mode='EdgesNumber',epg_collapse_par=3)
st.plot_dimension_reduction(adata,show_graph=True,show_text=False, plotly=True)
st.plot_branches(adata,show_text=False, plotly=True)
```

```
Collasping small branches ...
[1]
 "Removing the terminal branch with nodes: 7 22 44"


[1]
 "Constructing tree 1 of 1 / Subset 1 of 1"


[1]
 "Computing EPG with 48 nodes on 2559 points and 3 dimensions"


[1]
 "Using a single core"


0.017 sec elapsed

Number of branches after pruning ElPiGraph: 4
```

In [37]:

```
###Extend leaf branch to reach further cells 
st.extend_elastic_principal_graph(adata, epg_ext_mode='WeigthedCentroid',epg_ext_par=0.8)
st.plot_dimension_reduction(adata,color=['label'],n_components=3,show_graph=True,show_text=True, plotly=True)
```

```
Extending leaves with additional nodes ...
Number of branches after extending leaves: 4
```

In [38]:

```
ROOT = "S3"
```

In [39]:

```
st.plot_flat_tree(adata,color=['label','branch_id_alias', f'S3_pseudotime'], dist_scale=0.5,show_graph=True,show_text=True)
```

In [40]:

```
st.plot_stream_sc(adata,root=ROOT, color=['label'],
                  dist_scale=0.3,show_graph=True,show_text=True)
```

In [41]:

```
st.plot_stream(adata,root=ROOT, color=['label'])
```

In [42]:

```
adata.obs.head()
```

Out[42]:

|  | label | label\_color | louvain | discard | n\_counts | n\_genes | pct\_genes | pct\_mt | kmeans | node | branch\_id | branch\_id\_alias | branch\_lam | branch\_dist | S0\_pseudotime | S4\_pseudotime | S3\_pseudotime | S2\_pseudotime | S1\_pseudotime |
| --- | --- | --- | --- | --- | --- | --- | --- | --- | --- | --- | --- | --- | --- | --- | --- | --- | --- | --- | --- |
| Lane1\_AAACCTGAGATGTCGG | RPC | #66c2a5 | 0 | False | 3222 | 1414 | 0.093173 | 0.034761 | cluster 7 | 6 | (49, 3) | (S3, S0) | 4.458407 | 0.903146 | 9.088535 | 14.304056 | 4.458407 | 17.268371 | 13.654737 |
| Lane1\_AAACCTGCAATCCAAC | RPC | #66c2a5 | 0 | False | 2551 | 1281 | 0.084410 | 0.038024 | cluster 7 | 6 | (49, 3) | (S3, S0) | 4.519410 | 0.749472 | 9.027533 | 14.243054 | 4.519410 | 17.207369 | 13.593734 |
| Lane1\_AAACCTGGTTCCTCCA | RGC | #fc8d62 | 6 | False | 10358 | 2996 | 0.197417 | 0.027708 | cluster 2 | 50 | (50, 3) | (S2, S0) | 0.066593 | 0.595877 | 8.113243 | 13.328764 | 21.660185 | 0.066593 | 12.679444 |
| Lane1\_AAACCTGTCCAATGGT | AC | #8da0cb | 2 | False | 2736 | 1410 | 0.092910 | 0.034722 | cluster 9 | 39 | (48, 3) | (S4, S0) | 4.432241 | 0.475334 | 0.783281 | 4.432241 | 14.330223 | 8.963117 | 5.349482 |
| Lane1\_AAACGGGAGGCAATTA | RPC | #66c2a5 | 1 | False | 9226 | 2755 | 0.181537 | 0.024388 | cluster 4 | 49 | (49, 3) | (S3, S0) | 0.000000 | 0.251461 | 13.546943 | 18.762464 | 0.000000 | 21.726779 | 18.113144 |

In [54]:

```
import matplotlib.pyplot as plt
adata.obs["S3_pseudotime"].plot.kde()
plt.axvline(adata.obs["S3_pseudotime"].quantile(.25), c="red")
plt.axvline(adata.obs["S3_pseudotime"].quantile(.75), c="red")
```

Out[54]:

```
<matplotlib.lines.Line2D at 0x7f49c0ae6490>
```

In [43]:

```
adata.obs.groupby("branch_id_alias").first()
```

Out[43]:

|  | label | label\_color | louvain | discard | n\_counts | n\_genes | pct\_genes | pct\_mt | kmeans | node | branch\_id | branch\_lam | branch\_dist | S0\_pseudotime | S4\_pseudotime | S3\_pseudotime | S2\_pseudotime | S1\_pseudotime |
| --- | --- | --- | --- | --- | --- | --- | --- | --- | --- | --- | --- | --- | --- | --- | --- | --- | --- | --- |
| branch\_id\_alias |  |  |  |  |  |  |  |  |  |  |  |  |  |  |  |  |  |  |
| (S1, S0) | Cones | #e78ac3 | 9 | False | 2464 | 1247 | 0.082169 | 0.018669 | cluster 5 | 51 | (51, 3) | 0.482728 | 0.007014 | 4.083473 | 9.298994 | 17.630416 | 12.263309 | 0.482728 |
| (S2, S0) | RGC | #fc8d62 | 6 | False | 10358 | 2996 | 0.197417 | 0.027708 | cluster 2 | 50 | (50, 3) | 0.066593 | 0.595877 | 8.113243 | 13.328764 | 21.660185 | 0.066593 | 12.679444 |
| (S3, S0) | RPC | #66c2a5 | 0 | False | 3222 | 1414 | 0.093173 | 0.034761 | cluster 7 | 6 | (49, 3) | 4.458407 | 0.903146 | 9.088535 | 14.304056 | 4.458407 | 17.268371 | 13.654737 |
| (S4, S0) | AC | #8da0cb | 2 | False | 2736 | 1410 | 0.092910 | 0.034722 | cluster 9 | 39 | (48, 3) | 4.432241 | 0.475334 | 0.783281 | 4.432241 | 14.330223 | 8.963117 | 5.349482 |

In [44]:

```
main_nodes = set(it.chain.from_iterable(adata.obs.groupby("branch_id_alias").first().branch_id.to_list()))
main_nodes
```

Out[44]:

```
{3, 48, 49, 50, 51}
```

In [45]:

```
trajectory_main_points_ls = []
for node in main_nodes:
    _subset = adata.obs.query(f"node == {node}")
    print(f"Node {node} has", _subset.shape[0], "cells", "with label", _subset.label.mode()[0])
    trajectory_main_points_ls.append(
        pd.Series( _subset.label.mode()[0], index=_subset.index)
    )
```

```
Node 3 has 17 cells with label NB2
Node 48 has 109 cells with label AC
Node 49 has 78 cells with label RPC
Node 50 has 133 cells with label RGC
Node 51 has 69 cells with label Cones
```

In [46]:

```
observations = pd.concat(trajectory_main_points_ls)
observations.value_counts()
```

Out[46]:

```
RGC      133
AC       109
RPC       78
Cones     69
NB2       17
dtype: int64
```

In [47]:

```
desc = adata.obs.query("label == 'NB1'")['S3_pseudotime'].describe()
desc
```

Out[47]:

```
count    161.000000
mean       8.415242
std        0.594124
min        6.865974
25%        8.011575
50%        8.493421
75%        8.947669
max       10.092439
Name: S3_pseudotime, dtype: float64
```

In [48]:

```
neuroblast_midpoint =  adata.obs.query(f"label == 'NB1' & S3_pseudotime > {desc['25%']} & S3_pseudotime < {desc['75%']}")
neuroblast_midpoint.head()
```

Out[48]:

|  | label | label\_color | louvain | discard | n\_counts | n\_genes | pct\_genes | pct\_mt | kmeans | node | branch\_id | branch\_id\_alias | branch\_lam | branch\_dist | S0\_pseudotime | S4\_pseudotime | S3\_pseudotime | S2\_pseudotime | S1\_pseudotime |
| --- | --- | --- | --- | --- | --- | --- | --- | --- | --- | --- | --- | --- | --- | --- | --- | --- | --- | --- | --- |
| Lane1\_AAAGCAAAGACCTAGG | NB1 | #ffd92f | 7 | False | 4420 | 1783 | 0.117488 | 0.033032 | cluster 8 | 17 | (49, 3) | (S3, S0) | 8.356464 | 0.565656 | 5.190479 | 10.406000 | 8.356464 | 13.370315 | 9.756680 |
| Lane1\_AAAGTAGTCATGTCTT | NB1 | #ffd92f | 7 | False | 4100 | 1779 | 0.117225 | 0.017073 | cluster 8 | 17 | (49, 3) | (S3, S0) | 8.769578 | 0.379094 | 4.777365 | 9.992886 | 8.769578 | 12.957201 | 9.343566 |
| Lane1\_AACCATGCATGAACCT | NB1 | #ffd92f | 7 | False | 3681 | 1634 | 0.107670 | 0.030155 | cluster 8 | 17 | (49, 3) | (S3, S0) | 8.222750 | 0.234512 | 5.324192 | 10.539714 | 8.222750 | 13.504029 | 9.890394 |
| Lane1\_ACACCAAAGATCGGGT | NB1 | #ffd92f | 7 | False | 5734 | 2054 | 0.135345 | 0.027381 | cluster 8 | 17 | (49, 3) | (S3, S0) | 8.793867 | 0.665413 | 4.753075 | 9.968597 | 8.793867 | 12.932912 | 9.319277 |
| Lane1\_ACAGCTAAGGCTCAGA | NB1 | #ffd92f | 7 | False | 4827 | 1799 | 0.118542 | 0.031697 | cluster 8 | 17 | (49, 3) | (S3, S0) | 8.322970 | 0.314386 | 5.223972 | 10.439493 | 8.322970 | 13.403808 | 9.790174 |

In [49]:

```
final_observations = observations.append(
    pd.Series(neuroblast_midpoint.label.unique()[0], index=neuroblast_midpoint.index),
    verify_integrity=True
)
final_observations.name = 'observation'
```

In [51]:

```
final_observations.value_counts()
```

Out[51]:

```
RGC      133
AC       109
NB1       79
RPC       78
Cones     69
NB2       17
Name: observation, dtype: int64
```

In [57]:

```
final_observations.to_frame().to_csv("pseudotime_inferred_observations.csv")
```

In [ ]:

```

```
