# Supplementary material for: scBoolSeq: Linking scRNA-seq statistics and Boolean dynamics
Source: PLoS Comput Biol. 2024 Jul 8;20(7):e1011620. doi: 10.1371/journal.pcbi.1011620 (PMC11257695; doi:10.1371/journal.pcbi.1011620)
Supplement: S1 Notebooks — The notebooks are provided as static HTML files, and Boolean networks as textual files in BoolNet format. See the Data availability statement for links to executable notebooks and code. (ZIP) [file pcbi.1011620.s001.zip › Case_Study_Early-born_Retinal_Neurons/3.- Retinal Differentiation BN Inference-Mode.html]

3.- Retinal Differentiation BN Inference-Mode


In [1]:

```
from IPython.display import display, HTML
display(HTML("<style>.container { width:95% !important; }</style>"))
import warnings
warnings.filterwarnings("ignore") # umap deprecation warnings related to numba do not concern us.
```

In [2]:

```
from pathlib import Path as path
import functools as fn

import numpy as np
import pandas as pd

import matplotlib.pyplot as plt

from scboolseq.utils import parse_data_directory, parse_pickles
from scboolseq import scBoolSeq

from markers import (
    positive_markers, # function to generate a configuration dictionnary from a set of marker genes
    states_and_markers, # Dictionnary containing phenotypes and their marker genes
    marker_genes, # pandas.Series containing simply the name of markers
)
```

In [3]:

```
%ls *csv
```

```
dorothea_mouse_tfs.csv                pseudotime_inferred_observations.csv
GSE122466_metadata_batch1.csv         scboolseq_inferred_observations.csv
GSE122466_Retina_vargenes_batch1.csv
```

In [4]:

```
data = pd.read_csv("GSE122466_Retina_vargenes_batch1.csv", index_col=0)
print(data.shape)
data.head()
```

```
(2673, 1650)
```

Out[4]:

|  | Tubb3 | Malat1 | Stmn2 | Fgf15 | Gap43 | Xist | Sncg | Hmgb2 | Top2a | Meg3 | ... | Prdm13 | Kif14 | Rpl24 | Etfb | Cd320 | Fam98b | Odf2 | Fbxo36 | Rbp4 | Pou4f2 |
| --- | --- | --- | --- | --- | --- | --- | --- | --- | --- | --- | --- | --- | --- | --- | --- | --- | --- | --- | --- | --- | --- |
| Lane1\_AAACCTGAGATGTCGG | 0.000000 | 14.687273 | 9.280150 | 10.278990 | 0.000000 | 0.000000 | 8.282469 | 10.863565 | 8.282469 | 8.282469 | ... | 0.0 | 0.0 | 11.737682 | 0.000000 | 0.000000 | 0.000000 | 0.000000 | 0.0 | 0.0 | 0.0 |
| Lane1\_AAACCTGCAATCCAAC | 0.000000 | 14.568977 | 0.000000 | 10.200911 | 0.000000 | 0.000000 | 0.000000 | 10.200911 | 0.000000 | 0.000000 | ... | 0.0 | 0.0 | 10.615642 | 9.616560 | 0.000000 | 8.618397 | 0.000000 | 0.0 | 0.0 | 0.0 |
| Lane1\_AAACCTGGTTCCTCCA | 12.822128 | 16.422850 | 11.681013 | 0.000000 | 12.722607 | 0.000000 | 12.615708 | 0.000000 | 0.000000 | 11.237565 | ... | 0.0 | 0.0 | 9.402599 | 6.607977 | 0.000000 | 6.607977 | 6.607977 | 0.0 | 0.0 | 0.0 |
| Lane1\_AAACCTGTCCAATGGT | 12.601411 | 16.106194 | 10.514703 | 0.000000 | 8.517658 | 0.000000 | 8.517658 | 10.099994 | 0.000000 | 0.000000 | ... | 0.0 | 0.0 | 10.099994 | 0.000000 | 8.517658 | 0.000000 | 0.000000 | 0.0 | 0.0 | 0.0 |
| Lane1\_AAACGGGAGGCAATTA | 0.000000 | 14.858160 | 0.000000 | 12.375060 | 7.766719 | 11.082672 | 6.773328 | 11.889742 | 11.461031 | 7.766719 | ... | 0.0 | 0.0 | 11.284220 | 6.773328 | 0.000000 | 8.349471 | 6.773328 | 0.0 | 0.0 | 0.0 |

5 rows × 1650 columns

In [5]:

```
obs_tags = pd.read_csv("pseudotime_inferred_observations.csv", index_col=0)
obs_tags.observation.value_counts()
```

Out[5]:

```
observation
RGC      138
AC       110
RPC       79
NB1       79
Cones     69
NB2       16
Name: count, dtype: int64
```

In [6]:

```
%%time
scbool = scBoolSeq(
    dor_threshold=0.995, # To retain one of the markers
    confidence=.75 # To maximize binarization
)
scbool.fit(data)
```

```
Computing bimodality index for 853/1650 genes
Computing bimodality index for 43/1650 genes
CPU times: user 1min, sys: 1.62 s, total: 1min 2s
Wall time: 12.3 s
```

Out[6]:

```
scBoolSeqBinarizer(confidence=0.75, dor_threshold=0.995)
```

**In a Jupyter environment, please rerun this cell to show the HTML representation or trust the notebook.   
On GitHub, the HTML representation is unable to render, please try loading this page with nbviewer.org.**

scBoolSeqBinarizer

```
scBoolSeqBinarizer(confidence=0.75, dor_threshold=0.995)
```

In [7]:

```
scbool.criteria_.Category.value_counts()
```

Out[7]:

```
Category
Bimodal     853
ZeroInf     723
Unimodal     74
Name: count, dtype: int64
```

In [8]:

```
scbool.criteria_.loc[marker_genes, :].Category.value_counts()
```

Out[8]:

```
Category
ZeroInf    12
Bimodal    10
Name: count, dtype: int64
```

In [9]:

```
%time bin_data = scbool.binarize(data)
```

```
CPU times: user 1.24 s, sys: 4 ms, total: 1.24 s
Wall time: 1.24 s
```

In [10]:

```
partial_bin_configs = bin_data[marker_genes]
partial_bin_configs.fillna('').head()
```

Out[10]:

|  | Penk | Sstr2 | Btg2 | Isl1 | Pou4f2 | Otx2 | Prc1 | Sox2 | Pou6f2 | Onecut2 | ... | Crx | Pcdh17 | Elavl4 | Rbp4 | Onecut1 | Top2a | Prox1 | Pax6 | Neurod4 | Hes1 |
| --- | --- | --- | --- | --- | --- | --- | --- | --- | --- | --- | --- | --- | --- | --- | --- | --- | --- | --- | --- | --- | --- |
| Lane1\_AAACCTGAGATGTCGG |  |  | 0.0 | 0.0 |  |  | 0.0 | 1.0 | 1.0 | 0.0 | ... |  |  | 0.0 |  |  | 1.0 |  | 1.0 |  | 1.0 |
| Lane1\_AAACCTGCAATCCAAC |  |  | 1.0 | 0.0 |  |  | 0.0 |  | 0.0 | 0.0 | ... |  |  | 0.0 |  |  | 0.0 | 1.0 | 1.0 |  | 1.0 |
| Lane1\_AAACCTGGTTCCTCCA |  |  | 0.0 | 1.0 |  |  | 0.0 |  | 1.0 | 0.0 | ... |  | 1.0 | 1.0 |  | 1.0 | 0.0 |  | 1.0 |  | 0.0 |
| Lane1\_AAACCTGTCCAATGGT |  |  | 1.0 | 1.0 |  |  | 0.0 |  | 0.0 | 0.0 | ... |  |  | 0.0 |  |  | 0.0 |  | 1.0 |  | 0.0 |
| Lane1\_AAACGGGAGGCAATTA |  |  | 1.0 | 0.0 |  |  | 1.0 |  | 0.0 | 0.0 | ... |  | 1.0 | 0.0 |  |  | 1.0 |  | 1.0 |  | 1.0 |

5 rows × 22 columns

In [11]:

```
binarized_groups = {}
for obs, frame in obs_tags.groupby('observation'):
    binarized_groups.update({
        obs: bin_data.loc[frame.index, :]
    })
```

In [12]:

```
binarized_groups.keys()
```

Out[12]:

```
dict_keys(['AC', 'Cones', 'NB1', 'NB2', 'RGC', 'RPC'])
```

### Aggregate groups via the mode¶

In [13]:

```
%%time
agg_obs_mode = []
for group_name, binarized_group in binarized_groups.items():
    _meta_bin = binarized_group.mode(dropna=False).loc[0, ].to_frame() # Only take the first mode, otherwise there can be multiple rows
    _meta_bin.columns = [group_name]
    agg_obs_mode.append(_meta_bin)
    
meta_mode = pd.concat(agg_obs_mode, axis=1).T
```

```
CPU times: user 2.23 s, sys: 12.7 ms, total: 2.25 s
Wall time: 2.24 s
```

In [14]:

```
for pheno, genes in states_and_markers.items():
    if pheno in meta_mode.index:
        print(pheno, "\t:\t", meta_mode.loc[pheno, list(genes)].to_dict())
```

```
RPC 	:	 {'Fos': 1.0, 'Sox2': 1.0, 'Hes1': 1.0}
NB1 	:	 {'Sstr2': nan, 'Top2a': 1.0, 'Penk': 1.0, 'Btg2': 1.0, 'Prc1': 1.0}
NB2 	:	 {'Pcdh17': nan, 'Neurod4': nan, 'Pax6': 1.0}
RGC 	:	 {'Elavl4': 1.0, 'Isl1': 1.0, 'Pou4f2': nan, 'Pou6f2': 1.0}
AC 	:	 {'Onecut2': 1.0, 'Prox1': nan}
Cones 	:	 {'Otx2': 1.0, 'Thrb': 1.0, 'Rbp4': nan, 'Crx': 1.0}
```

In [30]:

```
meta_mode[marker_genes].T
```

Out[30]:

|  | AC | Cones | NB1 | NB2 | RGC | RPC |
| --- | --- | --- | --- | --- | --- | --- |
| Crx | NaN | 1.0 | NaN | NaN | NaN | NaN |
| Onecut1 | NaN | NaN | NaN | NaN | NaN | NaN |
| Neurod4 | NaN | 1.0 | NaN | NaN | NaN | NaN |
| Prox1 | NaN | NaN | NaN | NaN | NaN | NaN |
| Hes1 | 0.0 | 0.0 | 0.0 | 0.0 | 0.0 | 1.0 |
| Sox2 | NaN | NaN | NaN | NaN | NaN | 1.0 |
| Sstr2 | NaN | NaN | NaN | NaN | NaN | NaN |
| Isl1 | 0.0 | 0.0 | 0.0 | 0.0 | 1.0 | 0.0 |
| Fos | 0.0 | 0.0 | 0.0 | 0.0 | 0.0 | 1.0 |
| Pax6 | 1.0 | 0.0 | 1.0 | 1.0 | 1.0 | 1.0 |
| Prc1 | 0.0 | 0.0 | 1.0 | 0.0 | 0.0 | 1.0 |
| Elavl4 | 0.0 | 0.0 | 0.0 | 0.0 | 1.0 | 0.0 |
| Otx2 | NaN | 1.0 | 1.0 | NaN | NaN | NaN |
| Penk | NaN | NaN | 1.0 | NaN | NaN | NaN |
| Pcdh17 | NaN | NaN | NaN | NaN | NaN | NaN |
| Top2a | 0.0 | 0.0 | 1.0 | 0.0 | 0.0 | 1.0 |
| Rbp4 | NaN | NaN | NaN | NaN | NaN | NaN |
| Pou6f2 | 0.0 | 0.0 | 0.0 | 0.0 | 1.0 | 0.0 |
| Thrb | NaN | 1.0 | NaN | NaN | NaN | NaN |
| Btg2 | 0.0 | 1.0 | 1.0 | 1.0 | 0.0 | 0.0 |
| Onecut2 | 1.0 | 1.0 | 0.0 | 0.0 | 1.0 | 0.0 |
| Pou4f2 | NaN | NaN | NaN | NaN | NaN | NaN |

In [91]:

```
null_var_mask = meta_mode.var(skipna=False) == 0
null_var_genes = meta_mode.columns[null_var_mask]
```

In [93]:

```
with open("mode_active_invariant_genes.txt", "w") as _active_genes, open("mode_inactive_invariant_genes.txt", "w") as _inactive_genes:
    _active_genes.write(f"Gene\n")
    _inactive_genes.write(f"Gene\n")
    for _gene, _val in meta_mode[null_var_genes].mode().T.itertuples(index=True):
        if _val:
            _active_genes.write(f"{_gene}\n")
        else:
            _inactive_genes.write(f"{_gene}\n")
```

In [83]:

```
print(meta_mode.shape)
#print(f"Removing {100*(meta_mode.var(skipna=False) == 0).mean():.2f}% of genes (because they zero variance)")
meta_mode_var = meta_mode #meta_mode[meta_mode.columns[~(meta_mode.var(skipna=False) == 0)]].copy(deep=True)
print(meta_mode_var.shape)
meta_mode_var.iloc[:, :10]
```

```
(6, 1650)
(6, 1650)
```

Out[83]:

|  | Tubb3 | Malat1 | Stmn2 | Fgf15 | Gap43 | Xist | Sncg | Hmgb2 | Top2a | Meg3 |
| --- | --- | --- | --- | --- | --- | --- | --- | --- | --- | --- |
| AC | 1.0 | NaN | 1.0 | 0.0 | 1.0 | 0.0 | 0.0 | 0.0 | 0.0 | 1.0 |
| Cones | 1.0 | 1.0 | 0.0 | 0.0 | 0.0 | 0.0 | 0.0 | 0.0 | 0.0 | 1.0 |
| NB1 | 1.0 | NaN | 0.0 | 1.0 | 0.0 | 0.0 | 0.0 | 1.0 | 1.0 | 0.0 |
| NB2 | 1.0 | NaN | 1.0 | 0.0 | 0.0 | 1.0 | 0.0 | 1.0 | 0.0 | 0.0 |
| RGC | 1.0 | NaN | 1.0 | 0.0 | 1.0 | 0.0 | 1.0 | 1.0 | 0.0 | 1.0 |
| RPC | 1.0 | NaN | 0.0 | 1.0 | 0.0 | 0.0 | 0.0 | 1.0 | 1.0 | 0.0 |

In [84]:

```
dorothea_db = pd.read_csv("dorothea_mouse_tfs.csv")
dorothea_db.columns = dorothea_db.columns[:-1].to_list() + ['sign']
dorothea_db.head()
```

Out[84]:

|  | tf | confidence | target | sign |
| --- | --- | --- | --- | --- |
| 0 | 4932411N23Rik | E | Smad4 | 1 |
| 1 | 4932411N23Rik | E | 0610030E20Rik | 1 |
| 2 | 4932411N23Rik | E | 1700017N19Rik | 1 |
| 3 | 4932411N23Rik | E | 4931428F04Rik | 1 |
| 4 | 4932411N23Rik | E | 4932438A13Rik | 1 |

In [85]:

```
def in_db(frame: pd.DataFrame, db: pd.DataFrame):
    is_tf = frame.columns.isin(db.tf)
    is_target = frame.columns.isin(db.target)
    in_db = pd.Series(
        fn.reduce(np.logical_or, [is_tf, is_target]), 
        index=frame.columns
    )
    return in_db
```

In [86]:

```
in_db(meta_mode_var, dorothea_db).mean()
```

Out[86]:

```
0.8987878787878788
```

In [87]:

```
in_db(meta_mode_var[marker_genes], dorothea_db).mean()
```

Out[87]:

```
1.0
```

In [97]:

```
meta_mode_var[null_var_genes].columns[~in_db(meta_mode_var[null_var_genes], dorothea_db)]
```

Out[97]:

```
Index(['Mir124a-1hg', 'Selk', 'Pold3', 'Foxp1', 'Gm26699', 'Hn1l', 'Snhg9',
       'AY036118', 'Gm42418', 'Zcchc11', 'BC003331', 'Gm26518', 'Rps18-ps3',
       'Mum1'],
      dtype='object')
```

In [98]:

```
print(meta_mode_var.shape)
meta_mode_var_db = meta_mode_var[meta_mode_var.columns[in_db(meta_mode_var, dorothea_db)]]
print(meta_mode_var_db.shape)
meta_mode_var_db.iloc[:, :10]
```

```
(6, 1650)
(6, 1483)
```

Out[98]:

|  | Tubb3 | Stmn2 | Fgf15 | Gap43 | Sncg | Hmgb2 | Top2a | Ccnd1 | Tubb2b | Ebf1 |
| --- | --- | --- | --- | --- | --- | --- | --- | --- | --- | --- |
| AC | 1.0 | 1.0 | 0.0 | 1.0 | 0.0 | 0.0 | 0.0 | 0.0 | 1.0 | 0.0 |
| Cones | 1.0 | 0.0 | 0.0 | 0.0 | 0.0 | 0.0 | 0.0 | 0.0 | 1.0 | 0.0 |
| NB1 | 1.0 | 0.0 | 1.0 | 0.0 | 0.0 | 1.0 | 1.0 | 1.0 | 1.0 | 0.0 |
| NB2 | 1.0 | 1.0 | 0.0 | 0.0 | 0.0 | 1.0 | 0.0 | 0.0 | 1.0 | 0.0 |
| RGC | 1.0 | 1.0 | 0.0 | 1.0 | 1.0 | 1.0 | 0.0 | 0.0 | 1.0 | 1.0 |
| RPC | 1.0 | 0.0 | 1.0 | 0.0 | 0.0 | 1.0 | 1.0 | 1.0 | 1.0 | 0.0 |

In [99]:

```
meta_mode_var_db.T.isna().mean()
```

Out[99]:

```
AC       0.469319
Cones    0.461902
NB1      0.449090
NB2      0.472016
RGC      0.459879
RPC      0.432232
dtype: float64
```

In [100]:

```
meta_mode_var_db.isna().mean().mean()
```

Out[100]:

```
0.4574061586873455
```

In [101]:

```
import networkx as nx
from grn import *
```

In [102]:

```
dorothea_db.confidence.unique()
```

Out[102]:

```
array(['E', 'D', 'B', 'A', 'C'], dtype=object)
```

In [103]:

```
# The whole grn is weakly connected if we consider all confidence levels
whole_grn = df_to_graph(dorothea_db.query("confidence != 'E' & confidence != 'D'"))
induced_grn = nx.induced_subgraph(whole_grn, meta_mode_var_db.columns)
len(whole_grn), len(induced_grn)
```

Out[103]:

```
(5201, 622)
```

In [104]:

```
core_tf_nw = extract_largest_scc(whole_grn)
len(core_tf_nw)
```

Out[104]:

```
157
```

In [105]:

```
prior_grn = nx.induced_subgraph(whole_grn, set(core_tf_nw).union(meta_mode_var_db.columns))
len(prior_grn)
```

Out[105]:

```
749
```

In [106]:

```
nx.is_weakly_connected(prior_grn)
```

Out[106]:

```
False
```

In [107]:

```
pkn_biggest_wcc = nx.subgraph(prior_grn, max(nx.weakly_connected_components(prior_grn), key=len))
len(pkn_biggest_wcc)
```

Out[107]:

```
644
```

In [108]:

```
all(i in pkn_biggest_wcc for i in core_tf_nw)
```

Out[108]:

```
True
```

In [109]:

```
marker_genes.isin(pkn_biggest_wcc).mean()
```

Out[109]:

```
0.6363636363636364
```

In [110]:

```
import bonesis
```

In [111]:

```
def df_to_bonesis_data(df: pd.DataFrame):
    data = {}
    for config, genes in df.iterrows():
        data.update({config: genes.dropna().to_dict()})
    return data
```

In [112]:

```
data_meta_mode = df_to_bonesis_data(meta_mode_var_db) 
data_meta_mode.keys()
```

Out[112]:

```
dict_keys(['AC', 'Cones', 'NB1', 'NB2', 'RGC', 'RPC'])
```

In [113]:

```
pkn = bonesis.domains.InfluenceGraph(
    pkn_biggest_wcc, maxclause=8, allow_skipping_nodes=True, canonic=False
)
```

In [115]:

```
bo = bonesis.BoNesis(pkn, data_meta_mode)
bo
```

Out[115]:

```
<bonesis.BoNesis at 0x7fced8a1ed50>
```

In [123]:

```
with open("mode_prior_grn.json", "w") as _prior_grn, open("mode_data_meta.json", "w") as _data:
    json.dump(nx.node_link_data(pkn.as_nx), _prior_grn)
    json.dump(data_meta_mode, _data)
```

In [116]:

```
~bo.obs("RPC") >= ~bo.obs("NB1") >= ~bo.obs("NB2")
~bo.obs("NB2") >= bo.fixed(~bo.obs("Cones"))
~bo.obs("NB2") >= bo.fixed(~bo.obs("RGC"))
~bo.obs("NB2") >= bo.fixed(~bo.obs("AC"))
None
```

In [117]:

```
import datetime
from pathlib import Path as path
from scboolseq.utils import Timer
```

In [118]:

```
date = datetime.datetime.now()
bo.maximize_nodes()
bo.maximize_strong_constants()
view = bonesis.NonStrongConstantNodesView(bo, mode="optN")
view.standalone(
    output_filename=f"optim_dorothea_v2_mode_{date.strftime('%Y-%m-%d')}.sh"
)
print("Standalone shell file written", flush=True)

kept_nodes = {}
with Timer("Optimizing NonStrongConstantNodesView(bo, mode='optN')"):
    for kept_nodes in view:
        break

_replica_grn = nx.subgraph(prior_grn, kept_nodes)
```

```
Standalone shell file written
Grounding...done in 1.0s
<class 'bonesis0.gil_utils.BGIteratorPersistent'>
Optimizing NonStrongConstantNodesView(bo, mode='optN'): 5328.99843
```

In [119]:

```
len(kept_nodes)
```

Out[119]:

```
243
```

In [120]:

```
marker_genes.isin(_replica_grn).mean()
```

Out[120]:

```
0.3181818181818182
```

In [121]:

```
bonesis.InfluenceGraph(_replica_grn)
```

```
# computing graph layout...
```

Out[121]:

In [122]:

```
EXPORT = True

if EXPORT:
    nx.nx_pydot.write_dot(extract_largest_scc(bonesis.InfluenceGraph(_replica_grn).as_nx), "mode_grn_scc_v1.dot")
    import json
    with open("mode_grn_optimized_v1.json", "w") as f:
        json.dump(nx.node_link_data(_replica_grn), f)
    
    with open("mode_pkn_bonesis_obj_optimized_v1.json", "w") as f:
        json.dump(nx.node_link_data(bonesis.InfluenceGraph(_replica_grn).as_nx), f)
    print("Exported")
else:
    print("Not exported")
```

```
Exported
```

In [ ]:

```

```
