# Supplementary material for: scBoolSeq: Linking scRNA-seq statistics and Boolean dynamics
Source: PLoS Comput Biol. 2024 Jul 8;20(7):e1011620. doi: 10.1371/journal.pcbi.1011620 (PMC11257695; doi:10.1371/journal.pcbi.1011620)
Supplement: S1 Notebooks — The notebooks are provided as static HTML files, and Boolean networks as textual files in BoolNet format. See the Data availability statement for links to executable notebooks and code. (ZIP) [file pcbi.1011620.s001.zip › Case_Study_Early-born_Retinal_Neurons/1.- Retinal Differentiation Clustering and Metadata.html]

1.- Retinal Differentiation Clustering and Metadata


# Cell Identification via Boolean Values + Clustering + Metadata¶

In this notebook we showcase how the coarse-grained values obtained with `scBoolSeq` can be used in order to resolve cell identities (given a set of markers).
We show that these binary values can be used to identify "unambiguous cells" which match only a single type of markers.
Subsequently these identified cells are used to resolve cluster identities (part standard scRNA-Seq analyses).
These results are consistent with what was found in the original article from which we took the data:

```
Giudice, Q. Lo, Leleu, M., Manno, G. La, & Fabre, P. J. (2019).
Single-cell transcriptional logic of cell-fate specification and axon guidance in early-born retinal neurons. 
Development (Cambridge), 146(17). https://doi.org/10.1242/dev.178103
```

GEO accession GSE122466

In [1]:

```
from IPython.display import display, HTML
display(HTML("<style>.container { width:95% !important; }</style>"))
import warnings
warnings.filterwarnings("ignore") # umap deprecation warnings related to numba do not concern us.
```

In [2]:

```
import functools as fn
import itertools as it

import numpy as np
import pandas as pd

import sklearn
from sklearn.utils import Bunch
from sklearn.decomposition import PCA
from sklearn.pipeline import Pipeline
from sklearn.manifold import TSNE
from sklearn.preprocessing import FunctionTransformer
from umap import umap_ as umap

import matplotlib.pyplot as plt
import matplotlib as mpl
import seaborn as sns
import plotnine as pn
from plotnine import *

import scanpy as sc

from scboolseq.utils import parse_data_directory, parse_pickles
from scboolseq import scBoolSeq
```

In [3]:

```
%ls *csv
```

```
dorothea_mouse_tfs.csv         GSE122466_Retina_vargenes_batch1.csv
GSE122466_metadata_batch1.csv  pseudotime_inferred_observations.csv
```

## HVGs binarisation with `scBoolSeq`¶

Here we import the Highly Variable Genes previously identified in notebook `0.- Highly Variable Gene Selection.ipynb`.
We used `STREAM` software (Single-cell Trajectories Reconstruction, Exploration And Mapping, github repo).

Overview of the pipeline:

- Import HVGs
- Train a `scBoolSeq` instance
  - Inspect the distribution of categories
- Define a series of markers (known a priori)
- Binarise (coarse-grain) the HVG pseudocount dataframe
- Query the binary dataframe for cells matching the defined markers

In [4]:

```
data = pd.read_csv("GSE122466_Retina_vargenes_batch1.csv", index_col=0)
print(data.shape)
data.head()
```

```
(2673, 1650)
```

Out[4]:

|  | Tubb3 | Malat1 | Stmn2 | Fgf15 | Gap43 | Xist | Sncg | Hmgb2 | Top2a | Meg3 | ... | Prdm13 | Kif14 | Rpl24 | Etfb | Cd320 | Fam98b | Odf2 | Fbxo36 | Rbp4 | Pou4f2 |
| --- | --- | --- | --- | --- | --- | --- | --- | --- | --- | --- | --- | --- | --- | --- | --- | --- | --- | --- | --- | --- | --- |
| Lane1\_AAACCTGAGATGTCGG | 0.000000 | 14.687273 | 9.280150 | 10.278990 | 0.000000 | 0.000000 | 8.282469 | 10.863565 | 8.282469 | 8.282469 | ... | 0.0 | 0.0 | 11.737682 | 0.000000 | 0.000000 | 0.000000 | 0.000000 | 0.0 | 0.0 | 0.0 |
| Lane1\_AAACCTGCAATCCAAC | 0.000000 | 14.568977 | 0.000000 | 10.200911 | 0.000000 | 0.000000 | 0.000000 | 10.200911 | 0.000000 | 0.000000 | ... | 0.0 | 0.0 | 10.615642 | 9.616560 | 0.000000 | 8.618397 | 0.000000 | 0.0 | 0.0 | 0.0 |
| Lane1\_AAACCTGGTTCCTCCA | 12.822128 | 16.422850 | 11.681013 | 0.000000 | 12.722607 | 0.000000 | 12.615708 | 0.000000 | 0.000000 | 11.237565 | ... | 0.0 | 0.0 | 9.402599 | 6.607977 | 0.000000 | 6.607977 | 6.607977 | 0.0 | 0.0 | 0.0 |
| Lane1\_AAACCTGTCCAATGGT | 12.601411 | 16.106194 | 10.514703 | 0.000000 | 8.517658 | 0.000000 | 8.517658 | 10.099994 | 0.000000 | 0.000000 | ... | 0.0 | 0.0 | 10.099994 | 0.000000 | 8.517658 | 0.000000 | 0.000000 | 0.0 | 0.0 | 0.0 |
| Lane1\_AAACGGGAGGCAATTA | 0.000000 | 14.858160 | 0.000000 | 12.375060 | 7.766719 | 11.082672 | 6.773328 | 11.889742 | 11.461031 | 7.766719 | ... | 0.0 | 0.0 | 11.284220 | 6.773328 | 0.000000 | 8.349471 | 6.773328 | 0.0 | 0.0 | 0.0 |

5 rows × 1650 columns

In [5]:

```
%%time
scbool = scBoolSeq(
    dor_threshold=0.995, # To retain one of the markers, otherwise it will be marked as discarded due to its high dropout rate
    confidence=.75 # To maximize binarization
)
scbool.fit(data)
```

```
Computing bimodality index for 853/1650 genes
Computing bimodality index for 43/1650 genes
CPU times: user 59 s, sys: 1.59 s, total: 1min
Wall time: 12.1 s
```

Out[5]:

```
scBoolSeqBinarizer(confidence=0.75, dor_threshold=0.995)
```

**In a Jupyter environment, please rerun this cell to show the HTML representation or trust the notebook.   
On GitHub, the HTML representation is unable to render, please try loading this page with nbviewer.org.**

scBoolSeqBinarizer

```
scBoolSeqBinarizer(confidence=0.75, dor_threshold=0.995)
```

In [6]:

```
scbool.criteria_.Category.value_counts()
```

Out[6]:

```
Category
Bimodal     853
ZeroInf     723
Unimodal     74
Name: count, dtype: int64
```

These are markers reported in the original article (used for cluster labelling).

In [7]:

```
from markers import (
    positive_markers, # function to generate a configuration dictionnary from a set of marker genes
    states_and_markers, # Dictionnary containing phenotypes and their marker genes
)

marker_genes = pd.Series(list(set(it.chain.from_iterable(states_and_markers.values()))))
marker_genes.shape[0]
```

Out[7]:

```
22
```

In [8]:

```
scbool.criteria_.loc[marker_genes, :].Category.value_counts()
```

Out[8]:

```
Category
ZeroInf    12
Bimodal    10
Name: count, dtype: int64
```

There are no unimodal marker genes.

In [9]:

```
%time bin_data = scbool.binarize(data)
```

```
CPU times: user 1.2 s, sys: 7.98 ms, total: 1.21 s
Wall time: 1.21 s
```

Get a partial view of how marker genes were binarized:

In [10]:

```
partial_bin_configs = bin_data[marker_genes]
partial_bin_configs.fillna('').head()
```

Out[10]:

|  | Rbp4 | Pax6 | Otx2 | Sox2 | Btg2 | Hes1 | Sstr2 | Elavl4 | Onecut1 | Prox1 | ... | Pou6f2 | Pcdh17 | Prc1 | Top2a | Thrb | Onecut2 | Fos | Crx | Pou4f2 | Penk |
| --- | --- | --- | --- | --- | --- | --- | --- | --- | --- | --- | --- | --- | --- | --- | --- | --- | --- | --- | --- | --- | --- |
| Lane1\_AAACCTGAGATGTCGG |  | 1.0 |  | 1.0 | 0.0 | 1.0 |  | 0.0 |  |  | ... | 1.0 |  | 0.0 | 1.0 |  | 0.0 | 0.0 |  |  |  |
| Lane1\_AAACCTGCAATCCAAC |  | 1.0 |  |  | 1.0 | 1.0 |  | 0.0 |  | 1.0 | ... | 0.0 |  | 0.0 | 0.0 |  | 0.0 | 1.0 |  |  |  |
| Lane1\_AAACCTGGTTCCTCCA |  | 1.0 |  |  | 0.0 | 0.0 |  | 1.0 | 1.0 |  | ... | 1.0 | 1.0 | 0.0 | 0.0 | 1.0 | 0.0 | 0.0 |  |  |  |
| Lane1\_AAACCTGTCCAATGGT |  | 1.0 |  |  | 1.0 | 0.0 |  | 0.0 |  |  | ... | 0.0 |  | 0.0 | 0.0 |  | 0.0 | 0.0 |  |  |  |
| Lane1\_AAACGGGAGGCAATTA |  | 1.0 |  |  | 1.0 | 1.0 |  | 0.0 |  |  | ... | 0.0 | 1.0 | 1.0 | 1.0 |  | 0.0 | 1.0 |  |  |  |

5 rows × 22 columns

In [11]:

```
prior_markers = {
    obs: positive_markers(markers)
    for obs, markers
    in states_and_markers.items()
}
prior_markers
```

Out[11]:

```
{'RPC': {'Hes1': 1, 'Fos': 1, 'Sox2': 1},
 'NB1': {'Sstr2': 1, 'Top2a': 1, 'Btg2': 1, 'Prc1': 1, 'Penk': 1},
 'NB2': {'Pax6': 1, 'Neurod4': 1, 'Pcdh17': 1},
 'RGC': {'Pou4f2': 1, 'Elavl4': 1, 'Isl1': 1, 'Pou6f2': 1},
 'AC': {'Prox1': 1, 'Onecut2': 1},
 'HC': {'Prox1': 1, 'Onecut1': 1},
 'Cones': {'Thrb': 1, 'Otx2': 1, 'Crx': 1, 'Rbp4': 1}}
```

In [12]:

```
obs_by_markers = {}
for _conf, _marker_dict in prior_markers.items():
    # Build a query with the genes and their values, configuration by configuration
    _conf_query = " & ".join(f"{_gene} == {_value:.1f}" for _gene, _value in _marker_dict.items())
    _matching = partial_bin_configs.fillna(-1.0).query(_conf_query).index
    obs_by_markers.update({_conf: _matching})
```

In [13]:

```
number_of_matching_obs = {conf: idx.shape[0] for conf, idx in obs_by_markers.items()}
number_of_matching_obs
```

Out[13]:

```
{'RPC': 254, 'NB1': 27, 'NB2': 33, 'RGC': 202, 'AC': 120, 'HC': 28, 'Cones': 8}
```

In [14]:

```
percentage_matching = {conf: 100*idx.shape[0]/partial_bin_configs.shape[0] for conf, idx in obs_by_markers.items()}
{k: f"{v:.2f}" for k, v in percentage_matching.items()}
```

Out[14]:

```
{'RPC': '9.50',
 'NB1': '1.01',
 'NB2': '1.23',
 'RGC': '7.56',
 'AC': '4.49',
 'HC': '1.05',
 'Cones': '0.30'}
```

In [15]:

```
int(sum(percentage_matching.values()))
```

Out[15]:

```
25
```

25% of cells have boolean values matching the markers reported in the original article.

## Check for multi-labelled cells¶

In [16]:

```
intersections_of_all_combinations = list(map(
    lambda x: (
        x, 
        obs_by_markers[x[0]].intersection(obs_by_markers[x[1]]).shape[0]
    ), 
    it.combinations(obs_by_markers, 2) # All possible pairwise combinations, without replacement
)) # This will contain a list of nested tuples as follows: ((conf_1, conf_2), number_of_cells_in_both_groups)
```

In [17]:

```
non_null_intersections = list(filter(
    lambda p1_p2_shape: p1_p2_shape[-1].shape[0] > 0,
    map(
        lambda x: (x, obs_by_markers[x[0]].intersection(obs_by_markers[x[1]])), 
        it.combinations(obs_by_markers, 2)
    )
))
print(len(non_null_intersections))
len(non_null_intersections) / len(intersections_of_all_combinations)
```

```
10
```

Out[17]:

```
0.47619047619047616
```

In [18]:

```
{(c1, c2): len(idx) for ((c1, c2), idx) in non_null_intersections}
```

Out[18]:

```
{('RPC', 'AC'): 3,
 ('RPC', 'HC'): 2,
 ('NB1', 'NB2'): 1,
 ('NB1', 'RGC'): 1,
 ('NB1', 'AC'): 2,
 ('NB1', 'HC'): 1,
 ('NB2', 'AC'): 5,
 ('RGC', 'AC'): 10,
 ('RGC', 'HC'): 3,
 ('AC', 'HC'): 23}
```

Most intersections are small, only Horizontal cells and Amacrine cells are hard to distinguish given that they have only two marker genes each and
one of them is common to both of them.

In [19]:

```
unambiguous_observations = {}
for _phenotype, _idx in obs_by_markers.items():
    _unambiguous = fn.reduce(
        lambda x, y: x.difference(y),
        [idx for ((p1, p2), idx) in non_null_intersections if p1 == _phenotype or p2 == _phenotype],
        _idx
    )
    unambiguous_observations.update({_phenotype: _unambiguous})
    _n_unambiguous = _unambiguous.shape[0]
    print(f"{_phenotype} has {_n_unambiguous} matching unambiguous configurations, {100*_n_unambiguous/number_of_matching_obs[_phenotype]:.2f}% of matching configs")
    
unlabelled_idx = data.index.difference(fn.reduce(lambda x, y: x.union(y), unambiguous_observations.values()))
```

```
RPC has 249 matching unambiguous configurations, 98.03% of matching configs
NB1 has 23 matching unambiguous configurations, 85.19% of matching configs
NB2 has 27 matching unambiguous configurations, 81.82% of matching configs
RGC has 191 matching unambiguous configurations, 94.55% of matching configs
AC has 81 matching unambiguous configurations, 67.50% of matching configs
HC has 3 matching unambiguous configurations, 10.71% of matching configs
Cones has 8 matching unambiguous configurations, 100.00% of matching configs
```

In [20]:

```
color_map = {
    "Unknown": "#e6e6e6", # gray
    "RPC": "#eb34cf", # pink
    "RGC": "#c74930", # red
    "AC": "#c90c19", # reder ?
    "NB1": "#3060c7", # dark blue
    "NB2": "#30c7c4", # cyan
    "Cones": "#71c730", # green
    "HC": "#c79a30", # gold
}

unambiguous_metadata = pd.DataFrame({
    "label": "Unknown"
}, index=data.index)
for _conf, _idx in unambiguous_observations.items():
    unambiguous_metadata.loc[_idx, "label"] = _conf

color = unambiguous_metadata.label.map(color_map)
color.name = "label_color"
unambiguous_metadata = unambiguous_metadata.join(color)

print(100 * unambiguous_metadata.label.value_counts() / unambiguous_metadata.shape[0])
unambiguous_metadata.head()
```

```
label
Unknown    78.226712
RPC         9.315376
RGC         7.145529
AC          3.030303
NB2         1.010101
NB1         0.860456
Cones       0.299289
HC          0.112233
Name: count, dtype: float64
```

Out[20]:

|  | label | label\_color |
| --- | --- | --- |
| Lane1\_AAACCTGAGATGTCGG | Unknown | #e6e6e6 |
| Lane1\_AAACCTGCAATCCAAC | Unknown | #e6e6e6 |
| Lane1\_AAACCTGGTTCCTCCA | Unknown | #e6e6e6 |
| Lane1\_AAACCTGTCCAATGGT | Unknown | #e6e6e6 |
| Lane1\_AAACGGGAGGCAATTA | Unknown | #e6e6e6 |

In [25]:

```
unambiguous_metadata.query("label != 'Unknown'").shape[0] / unambiguous_metadata.shape[0]
```

Out[25]:

```
0.21773288439955107
```

In [33]:

```
EXPORT_METADATA = True

if EXPORT_METADATA:
    (
        unambiguous_metadata
            .query("label != 'Unknown'")
            .rename(columns={"label": "observation"})
            .observation
            .to_frame()
            .to_csv("scboolseq_inferred_observations.csv")
    )
    #unambiguous_metadata.to_csv("GSE122466_unambiguous_metadata_batch1.csv")
```

In [23]:

```
N_PCS = 25
```

In [24]:

```
%%time
projection_pipeline = Pipeline([
    ('pca', PCA()),
    ('subset_max_pcas', FunctionTransformer(lambda x: x.iloc[:, :N_PCS])), 
    ('umap', umap.UMAP())
])
reduced_umap = projection_pipeline.fit_transform(data)
projected_umap = pd.DataFrame(reduced_umap, index=data.index)
```

```
CPU times: user 1min 35s, sys: 24.7 s, total: 1min 59s
Wall time: 19 s
```

In [25]:

```
%%time
projection_pipeline2 = Pipeline([
    ('pca', PCA()),
    ('subset_max_pcas', FunctionTransformer(lambda x: x.iloc[:, :N_PCS])), 
    ('tsne', TSNE())
])
reduced_tsne = projection_pipeline2.fit_transform(data)
projected_tsne = pd.DataFrame(reduced_tsne, index=data.index)
```

```
CPU times: user 1min 34s, sys: 16.1 s, total: 1min 50s
Wall time: 10.9 s
```

To visualise the cummulative variance explained by top PCs:

```
(
    pd.Series(projection_pipeline['pca'].explained_variance_ratio_)
        .cumsum()
        .to_frame()
        .reset_index()
        .rename(columns={'index': 'n_components', 0: 'cummulative_explained_variance'})
        .plot.scatter(x='n_components', y='cummulative_explained_variance')
)
plt.grid()
plt.axvline(N_PCS)
```

In [26]:

```
fig, axs = plt.subplots(nrows=1, ncols=2, figsize=(16,5))

_tsne_i = 0
_umap_i = 1
_ = axs[_umap_i].scatter(projected_umap.loc[unlabelled_idx, 0], projected_umap.loc[unlabelled_idx, 1], label="Unknown", alpha=.5, c=color_map["Unknown"])
for name, idx in unambiguous_observations.items():
        _x, _y = projected_umap.loc[idx, 0], projected_umap.loc[idx, 1]
        axs[_umap_i].scatter(_x, _y, label=name, alpha=1 if name == 'HC' else .3)#, c=color_map[name])

axs[_umap_i].set_xlabel("UMAP 1")
axs[_umap_i].set_ylabel("UMAP 2")
#axs[0].legend()

_xname = projected_tsne.columns[0]
_yname = projected_tsne.columns[1]
_ = axs[_tsne_i].scatter(projected_tsne.loc[unlabelled_idx, _xname], projected_tsne.loc[unlabelled_idx, _yname], label="Unknown", alpha=.3, c=color_map["Unknown"])
for name, idx in unambiguous_observations.items():
        _x, _y = projected_tsne.loc[idx, _xname], projected_tsne.loc[idx, _yname]
        axs[_tsne_i].scatter(_x, _y, label=name, alpha=1 if name == 'HC' else .3)#, c=color_map[name])
axs[_tsne_i].set_xlabel("tSNE 1")
axs[_tsne_i].set_ylabel("tSNE 2")
#axs[1].legend()
handles, labels = plt.gca().get_legend_handles_labels()
by_label = dict(zip(labels, handles))
plt.legend(by_label.values(), by_label.keys())

plt.suptitle("Cell labels determined by Boolean marker gene signatures")
fig.tight_layout()
```

In [27]:

```
fig.savefig("cell_labels.pdf", format="pdf")
```

On the previous cell we can observe two distinct nonlinear dimensionality reduction techniques (t-SNE and UMAP) applied to the first
25 principal components. Cell labels are the unambiguous identities that we determined using `scBoolSeq`'s Boolean values.

These nonlinear and stochastic dimensionality reduction techniques always yield slightly different projections if no random state is given.
Nevertheless both the t-SNE and UMAP plots remain topologically coherent with those shown in the original article.

A small group of cells can be observed at the bottom center-right region of the UMAP plot (`UMAP_1 < 5` and `UMAP_2 < 0`.
This cluster was reported as well in the original article from which we cite the following description:

```
The smallest cluster (2.9% of the cells) was positive for mitochondrial genes and lacking Rps/Rpl genes (cluster 13) (Fig. 1I, Table S1); 
 this cluster was designated as unknown/RGC-like (U/RGC) as it is positive for RGC markers (Pou6f2, Pou4f1, Isl1, Islr2, Syt4, Ebf1/3 and L1cam) 
 but these cells may be RGCs with poor viability outcome, or originating from an alternative source such as the ciliary margin zone (Bélanger et al., 2017;
 Marcucci et al., 2016). These main neuronal clusters were validated with in situ hybridization (ISH) and immunohistochemistry (Fig. S3).
```

We can verify that our Boolean labels correspond to this description (after the figure).

In [32]:

```
isolated_cells = (
    projected_umap
        .rename(columns={c: f"umap_{c}" for c in projected_umap.columns})
        .query("umap_0 > umap_0.mean() & umap_1 < 0")
        .index
)
```

In [33]:

```
print(f"{100*isolated_cells.shape[0]/data.shape[0]:.1f}% of cells are in this region/cluster")
```

```
4.3% of cells are in this region/cluster
```

In [34]:

```
isolated_cells_labels: set = set()
for name, idx in unambiguous_observations.items():
    if isolated_cells.isin(idx).any():
        isolated_cells_labels.update({name})
        
isolated_cells_labels
```

Out[34]:

```
{'RGC'}
```

We obtain a similar fraction of cells and the same predicted label via our Boolean analysis as with the quantitative one presented in the original work.

## Clustering and label propagation¶

We will now look at unsupervised clustering (louvain) which is common practice in scRNA-Seq.
We will use a voting scheme to label the unsupervised clusters: Each cluster recieves the label of the majority of cells within (we only consider cells which are actually labelled).
In this part, we use the `scanpy` package for scRNA-Seq analysis.

In [35]:

```
sc.__version__
```

Out[35]:

```
'1.9.5'
```

In [36]:

```
adata = sc.AnnData(data, dtype=np.float64)
adata
```

Out[36]:

```
AnnData object with n_obs × n_vars = 2673 × 1650
```

In [37]:

```
# This is necessary because scBoolSeq sets the transform_output="pandas" which cannot be handled by scanpy==1.9.5
with sklearn.config_context(transform_output="default"):
    sc.pp.pca(adata, n_comps=N_PCS)
```

In [38]:

```
sc.pl.pca_variance_ratio(adata, log=True, n_pcs=N_PCS)
```

In [39]:

```
sc.pp.neighbors(adata, n_neighbors=15, n_pcs=N_PCS)
adata
```

Out[39]:

```
AnnData object with n_obs × n_vars = 2673 × 1650
    uns: 'pca', 'neighbors'
    obsm: 'X_pca'
    varm: 'PCs'
    obsp: 'distances', 'connectivities'
```

In [40]:

```
sc.tl.louvain(adata)
```

In [41]:

```
100 * adata.obs.louvain.value_counts() / data.shape[0]
```

Out[41]:

```
louvain
0     16.573139
1     12.832024
2     12.383090
3     10.699588
4      9.988777
5      7.369996
6      7.033296
7      6.023195
8      5.013094
9      4.826038
10     4.264871
11     2.992892
Name: count, dtype: float64
```

In [42]:

```
gg_umap = projected_umap.join(adata.obs.louvain).copy(deep=True).rename(columns={0: 'umap_1', 1: 'umap_2'})
gg_tsne = projected_tsne.join(adata.obs.louvain).copy(deep=True).rename(columns={0: 't-SNE_1', 1: 't-SNE_2'})
```

In [43]:

```
_plot_frame = gg_umap
first_plot = (
    ggplot(_plot_frame, aes(x=_plot_frame.columns[0], y=_plot_frame.columns[1]))
        + geom_point(aes(color='louvain'), alpha=.5)
)
first_plot
```

Out[43]:

```
<Figure Size: (640 x 480)>
```

In [44]:

```
_to_discard = _plot_frame.query("umap_1 > umap_1.mean() & umap_2 < 0").louvain.unique()
_to_discard
```

Out[44]:

```
['10']
Categories (12, object): ['0', '1', '2', '3', ..., '8', '9', '10', '11']
```

In [45]:

```
discarded_louvain = _to_discard[0]
discarded_louvain
```

Out[45]:

```
'10'
```

This cluster is to be discarded, as it does not sufficiently map to any known phenotype.
It was also discarded in the original paper (as detailed above).

In [46]:

```
pheno_by_cluster = {}
for clus, frame in _plot_frame.groupby('louvain'):
    louv_idx = frame.index
    pheno_by_cluster.update({
        clus: {
            pheno: louv_idx.isin(pheno_idx).sum()
            for pheno, pheno_idx
            in unambiguous_observations.items()
        }
    })

pheno_by_cluster
```

Out[46]:

```
{'0': {'RPC': 88, 'NB1': 0, 'NB2': 0, 'RGC': 0, 'AC': 0, 'HC': 1, 'Cones': 0},
 '1': {'RPC': 127, 'NB1': 0, 'NB2': 0, 'RGC': 0, 'AC': 2, 'HC': 0, 'Cones': 0},
 '2': {'RPC': 1, 'NB1': 0, 'NB2': 6, 'RGC': 0, 'AC': 49, 'HC': 1, 'Cones': 0},
 '3': {'RPC': 1, 'NB1': 0, 'NB2': 14, 'RGC': 0, 'AC': 9, 'HC': 1, 'Cones': 1},
 '4': {'RPC': 5, 'NB1': 0, 'NB2': 0, 'RGC': 66, 'AC': 9, 'HC': 0, 'Cones': 0},
 '5': {'RPC': 24, 'NB1': 0, 'NB2': 0, 'RGC': 0, 'AC': 0, 'HC': 0, 'Cones': 0},
 '6': {'RPC': 0, 'NB1': 0, 'NB2': 1, 'RGC': 77, 'AC': 3, 'HC': 0, 'Cones': 0},
 '7': {'RPC': 3, 'NB1': 23, 'NB2': 5, 'RGC': 0, 'AC': 4, 'HC': 0, 'Cones': 0},
 '8': {'RPC': 0, 'NB1': 0, 'NB2': 0, 'RGC': 26, 'AC': 3, 'HC': 0, 'Cones': 0},
 '9': {'RPC': 0, 'NB1': 0, 'NB2': 1, 'RGC': 0, 'AC': 0, 'HC': 0, 'Cones': 7},
 '10': {'RPC': 0, 'NB1': 0, 'NB2': 0, 'RGC': 2, 'AC': 0, 'HC': 0, 'Cones': 0},
 '11': {'RPC': 0, 'NB1': 0, 'NB2': 0, 'RGC': 20, 'AC': 2, 'HC': 0, 'Cones': 0}}
```

These are the number of observations matching each one of the labels, for every louvain cluster.

In [47]:

```
majority_pheno_by_cluster = {}
for clus, votes in pheno_by_cluster.items():
    votes = sorted(votes.items(), key=lambda item: item[1], reverse=True)
    _total = sum(v for _, v in votes)
    relative_votes = [(name, v/_total) for name, v in votes] 
    print(clus, relative_votes)
    majority_pheno_by_cluster.update({clus: relative_votes[0][0]})
```

```
0 [('RPC', 0.9887640449438202), ('HC', 0.011235955056179775), ('NB1', 0.0), ('NB2', 0.0), ('RGC', 0.0), ('AC', 0.0), ('Cones', 0.0)]
1 [('RPC', 0.9844961240310077), ('AC', 0.015503875968992248), ('NB1', 0.0), ('NB2', 0.0), ('RGC', 0.0), ('HC', 0.0), ('Cones', 0.0)]
2 [('AC', 0.8596491228070176), ('NB2', 0.10526315789473684), ('RPC', 0.017543859649122806), ('HC', 0.017543859649122806), ('NB1', 0.0), ('RGC', 0.0), ('Cones', 0.0)]
3 [('NB2', 0.5384615384615384), ('AC', 0.34615384615384615), ('RPC', 0.038461538461538464), ('HC', 0.038461538461538464), ('Cones', 0.038461538461538464), ('NB1', 0.0), ('RGC', 0.0)]
4 [('RGC', 0.825), ('AC', 0.1125), ('RPC', 0.0625), ('NB1', 0.0), ('NB2', 0.0), ('HC', 0.0), ('Cones', 0.0)]
5 [('RPC', 1.0), ('NB1', 0.0), ('NB2', 0.0), ('RGC', 0.0), ('AC', 0.0), ('HC', 0.0), ('Cones', 0.0)]
6 [('RGC', 0.9506172839506173), ('AC', 0.037037037037037035), ('NB2', 0.012345679012345678), ('RPC', 0.0), ('NB1', 0.0), ('HC', 0.0), ('Cones', 0.0)]
7 [('NB1', 0.6571428571428571), ('NB2', 0.14285714285714285), ('AC', 0.11428571428571428), ('RPC', 0.08571428571428572), ('RGC', 0.0), ('HC', 0.0), ('Cones', 0.0)]
8 [('RGC', 0.896551724137931), ('AC', 0.10344827586206896), ('RPC', 0.0), ('NB1', 0.0), ('NB2', 0.0), ('HC', 0.0), ('Cones', 0.0)]
9 [('Cones', 0.875), ('NB2', 0.125), ('RPC', 0.0), ('NB1', 0.0), ('RGC', 0.0), ('AC', 0.0), ('HC', 0.0)]
10 [('RGC', 1.0), ('RPC', 0.0), ('NB1', 0.0), ('NB2', 0.0), ('AC', 0.0), ('HC', 0.0), ('Cones', 0.0)]
11 [('RGC', 0.9090909090909091), ('AC', 0.09090909090909091), ('RPC', 0.0), ('NB1', 0.0), ('NB2', 0.0), ('HC', 0.0), ('Cones', 0.0)]
```

Here, we select the majority label for each one of the clusters.
It is to be noted that most clusters have clear consensus on labels, with the sole exception of `cluster 3` which has higher impurity:

`3 [('NB2', 0.5384615384615384), ('AC', 0.34615384615384615), ('RPC', 0.038461538461538464), ('HC', 0.038461538461538464), ('Cones', 0.038461538461538464), ('NB1', 0.0), ('RGC', 0.0)]`

In [48]:

```
represented_by_louvain = set(majority_pheno_by_cluster.values())
phenotypes = set(unambiguous_observations)
```

In [49]:

```
100 * len(represented_by_louvain.intersection(phenotypes)) / len(phenotypes)
```

Out[49]:

```
85.71428571428571
```

In [50]:

```
phenotypes.difference(represented_by_louvain)
```

Out[50]:

```
{'HC'}
```

All phenotypes were assigned to at least one of the clusters with the sole exception of `Horizontal Cells`

In [51]:

```
_new_plot = _plot_frame.join(_plot_frame.louvain.map(majority_pheno_by_cluster).to_frame().rename(columns={"louvain": "phenotype"}))
_new_plot.head()
```

Out[51]:

|  | umap\_1 | umap\_2 | louvain | phenotype |
| --- | --- | --- | --- | --- |
| Lane1\_AAACCTGAGATGTCGG | -4.445245 | 9.471989 | 0 | RPC |
| Lane1\_AAACCTGCAATCCAAC | -4.260791 | 9.667463 | 0 | RPC |
| Lane1\_AAACCTGGTTCCTCCA | 11.077205 | 11.888295 | 6 | RGC |
| Lane1\_AAACCTGTCCAATGGT | 5.158805 | 6.394330 | 2 | AC |
| Lane1\_AAACGGGAGGCAATTA | -2.875255 | 14.834937 | 1 | RPC |

In [52]:

```
second_plot = (
    ggplot(_new_plot, aes(x=_plot_frame.columns[0], y=_plot_frame.columns[1]))
        + geom_point(aes(color='phenotype'), alpha=.5)
)
second_plot
```

Out[52]:

```
<Figure Size: (640 x 480)>
```

In [53]:

```
palette = sns.color_palette('Set2', _plot_frame.louvain.unique().shape[0])
palette.as_hex()
```

Out[53]:

In [54]:

```
final_color_map = dict(zip(_new_plot.phenotype.unique(),palette.as_hex()))
final_color_map
```

Out[54]:

```
{'RPC': '#66c2a5',
 'RGC': '#fc8d62',
 'AC': '#8da0cb',
 'Cones': '#e78ac3',
 'NB2': '#a6d854',
 'NB1': '#ffd92f'}
```

In [55]:

```
metadata = pd.DataFrame({
    'label': _new_plot.phenotype,
    'label_color': _new_plot.phenotype.map(final_color_map),
    'louvain': _new_plot.louvain,
})
metadata.loc[:, 'discard'] = False
metadata.loc[metadata.louvain == discarded_louvain, 'discard'] = True
metadata.head()
```

Out[55]:

|  | label | label\_color | louvain | discard |
| --- | --- | --- | --- | --- |
| Lane1\_AAACCTGAGATGTCGG | RPC | #66c2a5 | 0 | False |
| Lane1\_AAACCTGCAATCCAAC | RPC | #66c2a5 | 0 | False |
| Lane1\_AAACCTGGTTCCTCCA | RGC | #fc8d62 | 6 | False |
| Lane1\_AAACCTGTCCAATGGT | AC | #8da0cb | 2 | False |
| Lane1\_AAACGGGAGGCAATTA | RPC | #66c2a5 | 1 | False |

In [56]:

```
metadata.discard.value_counts()
```

Out[56]:

```
discard
False    2559
True      114
Name: count, dtype: int64
```

## Exporting metadata¶

Here we export data obtained in order to perform trajectory inference using `STREAM`.

In [57]:

```
metadata.to_csv("GSE122466_metadata_batch1_v3.tsv", sep="\t")
```

In [ ]:

```

```
