# Supplementary material for: scBoolSeq: Linking scRNA-seq statistics and Boolean dynamics
Source: PLoS Comput Biol. 2024 Jul 8;20(7):e1011620. doi: 10.1371/journal.pcbi.1011620 (PMC11257695; doi:10.1371/journal.pcbi.1011620)
Supplement: S1 Notebooks — The notebooks are provided as static HTML files, and Boolean networks as textual files in BoolNet format. See the Data availability statement for links to executable notebooks and code. (ZIP) [file pcbi.1011620.s001.zip › Case_Study_Early-born_Retinal_Neurons/3.- Retinal Differentiation BN Inference-Bootstrap.html]

3.- Retinal Differentiation BN Inference-Bootstrap


In [1]:

```
from IPython.display import display, HTML
display(HTML("<style>.container { width:95% !important; }</style>"))
import warnings
warnings.filterwarnings("ignore") # umap deprecation warnings related to numba do not concern us.
```

In [2]:

```
from pathlib import Path as path
import functools as fn

import numpy as np
import pandas as pd
import matplotlib.pyplot as plt
from sklearn.utils import Bunch

from scboolseq.utils import parse_data_directory, parse_pickles
from scboolseq.meta import bootstrap
from scboolseq import scBoolSeq

from markers import (
    positive_markers, # function to generate a configuration dictionnary from a set of marker genes
    states_and_markers, # Dictionnary containing phenotypes and their marker genes
    marker_genes, # pandas.Series containing simply the name of markers
)
```

In [3]:

```
%ls *csv
```

```
dorothea_mouse_tfs.csv                pseudotime_inferred_observations.csv
GSE122466_metadata_batch1.csv         scboolseq_inferred_observations.csv
GSE122466_Retina_vargenes_batch1.csv
```

In [4]:

```
data = pd.read_csv("GSE122466_Retina_vargenes_batch1.csv", index_col=0)
print(data.shape)
data.head()
```

```
(2673, 1650)
```

Out[4]:

|  | Tubb3 | Malat1 | Stmn2 | Fgf15 | Gap43 | Xist | Sncg | Hmgb2 | Top2a | Meg3 | ... | Prdm13 | Kif14 | Rpl24 | Etfb | Cd320 | Fam98b | Odf2 | Fbxo36 | Rbp4 | Pou4f2 |
| --- | --- | --- | --- | --- | --- | --- | --- | --- | --- | --- | --- | --- | --- | --- | --- | --- | --- | --- | --- | --- | --- |
| Lane1\_AAACCTGAGATGTCGG | 0.000000 | 14.687273 | 9.280150 | 10.278990 | 0.000000 | 0.000000 | 8.282469 | 10.863565 | 8.282469 | 8.282469 | ... | 0.0 | 0.0 | 11.737682 | 0.000000 | 0.000000 | 0.000000 | 0.000000 | 0.0 | 0.0 | 0.0 |
| Lane1\_AAACCTGCAATCCAAC | 0.000000 | 14.568977 | 0.000000 | 10.200911 | 0.000000 | 0.000000 | 0.000000 | 10.200911 | 0.000000 | 0.000000 | ... | 0.0 | 0.0 | 10.615642 | 9.616560 | 0.000000 | 8.618397 | 0.000000 | 0.0 | 0.0 | 0.0 |
| Lane1\_AAACCTGGTTCCTCCA | 12.822128 | 16.422850 | 11.681013 | 0.000000 | 12.722607 | 0.000000 | 12.615708 | 0.000000 | 0.000000 | 11.237565 | ... | 0.0 | 0.0 | 9.402599 | 6.607977 | 0.000000 | 6.607977 | 6.607977 | 0.0 | 0.0 | 0.0 |
| Lane1\_AAACCTGTCCAATGGT | 12.601411 | 16.106194 | 10.514703 | 0.000000 | 8.517658 | 0.000000 | 8.517658 | 10.099994 | 0.000000 | 0.000000 | ... | 0.0 | 0.0 | 10.099994 | 0.000000 | 8.517658 | 0.000000 | 0.000000 | 0.0 | 0.0 | 0.0 |
| Lane1\_AAACGGGAGGCAATTA | 0.000000 | 14.858160 | 0.000000 | 12.375060 | 7.766719 | 11.082672 | 6.773328 | 11.889742 | 11.461031 | 7.766719 | ... | 0.0 | 0.0 | 11.284220 | 6.773328 | 0.000000 | 8.349471 | 6.773328 | 0.0 | 0.0 | 0.0 |

5 rows × 1650 columns

In [5]:

```
obs_tags = pd.read_csv("scboolseq_inferred_observations.csv", index_col=0)
obs_tags.observation.value_counts()
```

Out[5]:

```
observation
RPC      249
RGC      191
AC        81
NB2       27
NB1       23
Cones      8
HC         3
Name: count, dtype: int64
```

In [6]:

```
%%time
scbool = scBoolSeq(
    dor_threshold=0.995, # To retain one of the markers
    confidence=.75 # To maximize binarization
)
scbool.fit(data)
```

```
Computing bimodality index for 853/1650 genes
Computing bimodality index for 43/1650 genes
CPU times: user 52.2 s, sys: 1.61 s, total: 53.8 s
Wall time: 10.2 s
```

Out[6]:

```
scBoolSeqBinarizer(confidence=0.75, dor_threshold=0.995)
```

**In a Jupyter environment, please rerun this cell to show the HTML representation or trust the notebook.   
On GitHub, the HTML representation is unable to render, please try loading this page with nbviewer.org.**

scBoolSeqBinarizer

```
scBoolSeqBinarizer(confidence=0.75, dor_threshold=0.995)
```

In [7]:

```
scbool.criteria_.Category.value_counts()
```

Out[7]:

```
Category
Bimodal     853
ZeroInf     723
Unimodal     74
Name: count, dtype: int64
```

In [8]:

```
scbool.criteria_.loc[marker_genes, :].Category.value_counts()
```

Out[8]:

```
Category
ZeroInf    12
Bimodal    10
Name: count, dtype: int64
```

In [9]:

```
%time bin_data = scbool.binarize(data)
```

```
CPU times: user 1.03 s, sys: 7.85 ms, total: 1.03 s
Wall time: 1.03 s
```

In [10]:

```
partial_bin_configs = bin_data[marker_genes]
partial_bin_configs.fillna('').head()
```

Out[10]:

|  | Pou4f2 | Crx | Rbp4 | Thrb | Btg2 | Top2a | Pax6 | Fos | Otx2 | Hes1 | ... | Elavl4 | Sstr2 | Isl1 | Pcdh17 | Prox1 | Neurod4 | Prc1 | Onecut1 | Pou6f2 | Penk |
| --- | --- | --- | --- | --- | --- | --- | --- | --- | --- | --- | --- | --- | --- | --- | --- | --- | --- | --- | --- | --- | --- |
| Lane1\_AAACCTGAGATGTCGG |  |  |  |  | 0.0 | 1.0 | 1.0 | 0.0 |  | 1.0 | ... | 0.0 |  | 0.0 |  |  |  | 0.0 |  | 1.0 |  |
| Lane1\_AAACCTGCAATCCAAC |  |  |  |  | 1.0 | 0.0 | 1.0 | 1.0 |  | 1.0 | ... | 0.0 |  | 0.0 |  | 1.0 |  | 0.0 |  | 0.0 |  |
| Lane1\_AAACCTGGTTCCTCCA |  |  |  | 1.0 | 0.0 | 0.0 | 1.0 | 0.0 |  | 0.0 | ... | 1.0 |  | 1.0 | 1.0 |  |  | 0.0 | 1.0 | 1.0 |  |
| Lane1\_AAACCTGTCCAATGGT |  |  |  |  | 1.0 | 0.0 | 1.0 | 0.0 |  | 0.0 | ... | 0.0 |  | 1.0 |  |  |  | 0.0 |  | 0.0 |  |
| Lane1\_AAACGGGAGGCAATTA |  |  |  |  | 1.0 | 1.0 | 1.0 | 1.0 |  | 1.0 | ... | 0.0 |  | 0.0 | 1.0 |  |  | 1.0 |  | 0.0 |  |

5 rows × 22 columns

In [11]:

```
binarized_groups = {}
for obs, frame in obs_tags.groupby('observation'):
    binarized_groups.update({
        obs: bin_data.loc[frame.index, :]
    })
```

In [12]:

```
binarized_groups.keys()
```

Out[12]:

```
dict_keys(['AC', 'Cones', 'HC', 'NB1', 'NB2', 'RGC', 'RPC'])
```

### Aggregate groups¶

Here we perform the following encoding:

$
\begin{equation}\label{eq:bimodalbin}
f(x) =
\begin{cases}
-1 & \text{if}\;\; x = False\\
0 & \text{if}\;\; x = \text{?}\\
1 & \text{if}\;\; x = True
\end{cases}
\end{equation}
$

And sum accross all observations with the same tag.

We then use a Bootstrap estimation of the mean value in order to binarise each group.

In [13]:

```
from scboolseq.meta import bootstrap
import multiprocessing as mp
```

In [20]:

```
%%time
mapped_binarized_groups = Bunch(**{
    group_name: binarized_group.replace(0.0, -1.0).fillna(0.0)
    for group_name, binarized_group 
    in binarized_groups.items()
})
```

```
CPU times: user 9.43 ms, sys: 0 ns, total: 9.43 ms
Wall time: 9.01 ms
```

In [59]:

```
def aggregate_bootstrap_frame(b_ci_frame: pd.DataFrame, name: str):
    positive_mask = (b_ci_frame > 0).all(axis=0)
    negative_mask = (b_ci_frame < 0).all(axis=0)
    aggregated = pd.Series(np.nan, index=b_ci_frame.columns, name=name)
    aggregated[positive_mask] = 1.0
    aggregated[negative_mask] = 0.0
    return aggregated
```

In [60]:

```
%%time

bootstrap_frames = {}
meta_bin_ls = []
for name, frame in mapped_binarized_groups.items():
    print(name)
    _b = frame.apply(bootstrap)
    bootstrap_frames.update({name: _b})
    meta_bin_ls.append(aggregate_bootstrap_frame(_b, name))
```

```
AC
Cones
HC
NB1
NB2
RGC
RPC
CPU times: user 3min 56s, sys: 54.4 ms, total: 3min 56s
Wall time: 3min 56s
```

In [73]:

```
meta_bootstrap_bin  = pd.concat(meta_bin_ls, axis=1).T
meta_bootstrap_bin
```

Out[73]:

|  | Tubb3 | Malat1 | Stmn2 | Fgf15 | Gap43 | Xist | Sncg | Hmgb2 | Top2a | Meg3 | ... | Prdm13 | Kif14 | Rpl24 | Etfb | Cd320 | Fam98b | Odf2 | Fbxo36 | Rbp4 | Pou4f2 |
| --- | --- | --- | --- | --- | --- | --- | --- | --- | --- | --- | --- | --- | --- | --- | --- | --- | --- | --- | --- | --- | --- |
| AC | 1.0 | NaN | 1.0 | 0.0 | 1.0 | NaN | NaN | NaN | 0.0 | 1.0 | ... | 1.0 | NaN | NaN | 0.0 | 1.0 | NaN | 1.0 | NaN | NaN | NaN |
| Cones | NaN | 1.0 | 0.0 | NaN | NaN | NaN | NaN | NaN | NaN | NaN | ... | NaN | NaN | NaN | NaN | NaN | NaN | NaN | NaN | 1.0 | NaN |
| HC | 1.0 | NaN | 1.0 | NaN | NaN | NaN | 0.0 | 1.0 | NaN | NaN | ... | NaN | NaN | NaN | 0.0 | NaN | NaN | NaN | NaN | NaN | NaN |
| NB1 | 1.0 | NaN | NaN | NaN | NaN | NaN | 0.0 | 1.0 | 1.0 | 0.0 | ... | NaN | NaN | NaN | NaN | NaN | 1.0 | 1.0 | NaN | NaN | NaN |
| NB2 | 1.0 | NaN | 1.0 | NaN | NaN | 0.0 | 0.0 | 1.0 | NaN | 1.0 | ... | NaN | NaN | NaN | 0.0 | NaN | NaN | NaN | NaN | NaN | NaN |
| RGC | 1.0 | NaN | 1.0 | 0.0 | 1.0 | 0.0 | 1.0 | 1.0 | 0.0 | 1.0 | ... | NaN | NaN | NaN | NaN | 1.0 | 0.0 | 1.0 | NaN | NaN | 1.0 |
| RPC | NaN | 0.0 | 0.0 | 1.0 | 0.0 | 0.0 | 0.0 | 1.0 | 1.0 | 0.0 | ... | NaN | 1.0 | 1.0 | 0.0 | 1.0 | 0.0 | 1.0 | 1.0 | NaN | NaN |

7 rows × 1650 columns

In [74]:

```
for pheno, genes in states_and_markers.items():
    if pheno in meta_bootstrap_bin.index:
        print(pheno, "\t:\t", meta_bootstrap_bin.loc[pheno, list(genes)].to_dict())
```

```
RPC 	:	 {'Fos': 1.0, 'Hes1': 1.0, 'Sox2': 1.0}
NB1 	:	 {'Penk': 1.0, 'Prc1': 1.0, 'Sstr2': 1.0, 'Top2a': 1.0, 'Btg2': 1.0}
NB2 	:	 {'Pax6': 1.0, 'Pcdh17': 1.0, 'Neurod4': 1.0}
RGC 	:	 {'Isl1': 1.0, 'Elavl4': 1.0, 'Pou6f2': 1.0, 'Pou4f2': 1.0}
AC 	:	 {'Prox1': 1.0, 'Onecut2': 1.0}
HC 	:	 {'Prox1': 1.0, 'Onecut1': 1.0}
Cones 	:	 {'Thrb': 1.0, 'Crx': 1.0, 'Otx2': 1.0, 'Rbp4': 1.0}
```

Bootstrapped estimations preserves all means.

In [75]:

```
meta_bootstrap_bin[marker_genes].T
```

Out[75]:

|  | AC | Cones | HC | NB1 | NB2 | RGC | RPC |
| --- | --- | --- | --- | --- | --- | --- | --- |
| Penk | 1.0 | NaN | NaN | 1.0 | 1.0 | NaN | NaN |
| Isl1 | 0.0 | 0.0 | 0.0 | 0.0 | 0.0 | 1.0 | 0.0 |
| Elavl4 | NaN | 0.0 | NaN | 0.0 | 0.0 | 1.0 | 0.0 |
| Fos | 0.0 | NaN | NaN | NaN | 0.0 | 0.0 | 1.0 |
| Otx2 | 1.0 | 1.0 | 1.0 | 1.0 | 1.0 | NaN | 1.0 |
| Pcdh17 | 1.0 | NaN | NaN | 1.0 | 1.0 | 1.0 | 1.0 |
| Btg2 | 0.0 | 1.0 | NaN | 1.0 | 1.0 | 0.0 | 0.0 |
| Pax6 | 1.0 | NaN | 1.0 | NaN | 1.0 | 1.0 | 1.0 |
| Prox1 | 1.0 | NaN | 1.0 | NaN | NaN | NaN | 1.0 |
| Rbp4 | NaN | 1.0 | NaN | NaN | NaN | NaN | NaN |
| Pou6f2 | 0.0 | 0.0 | 0.0 | 0.0 | 0.0 | 1.0 | 0.0 |
| Top2a | 0.0 | NaN | NaN | 1.0 | NaN | 0.0 | 1.0 |
| Hes1 | 0.0 | 0.0 | 0.0 | 0.0 | 0.0 | 0.0 | 1.0 |
| Sstr2 | NaN | NaN | NaN | 1.0 | NaN | NaN | NaN |
| Crx | NaN | 1.0 | NaN | NaN | 1.0 | NaN | NaN |
| Onecut2 | 1.0 | NaN | 0.0 | 0.0 | NaN | 1.0 | 0.0 |
| Thrb | NaN | 1.0 | NaN | NaN | NaN | NaN | 1.0 |
| Sox2 | NaN | NaN | NaN | 1.0 | NaN | NaN | 1.0 |
| Prc1 | 0.0 | NaN | NaN | 1.0 | 0.0 | 0.0 | 1.0 |
| Pou4f2 | NaN | NaN | NaN | NaN | NaN | 1.0 | NaN |
| Onecut1 | NaN | NaN | 1.0 | 1.0 | NaN | 1.0 | NaN |
| Neurod4 | NaN | 1.0 | NaN | NaN | 1.0 | NaN | NaN |

In [76]:

```
null_var_mask = meta_bootstrap_bin.var(skipna=False) == 0
null_var_genes = meta_bootstrap_bin.columns[null_var_mask]
null_var_genes.shape
```

Out[76]:

```
(39,)
```

In [77]:

```
with open("bootstrap_active_invariant_genes.txt", "w") as _active_genes, open("bootstrap_inactive_invariant_genes.txt", "w") as _inactive_genes:
    _active_genes.write(f"Gene\n")
    _inactive_genes.write(f"Gene\n")
    for _gene, _val in meta_bootstrap_bin[null_var_genes].mode().T.itertuples(index=True):
        if _val:
            _active_genes.write(f"{_gene}\n")
        else:
            _inactive_genes.write(f"{_gene}\n")
```

In [78]:

```
print(meta_bootstrap_bin.shape)
meta_boot = meta_bootstrap_bin
meta_boot.iloc[:, :10]
```

```
(7, 1650)
```

Out[78]:

|  | Tubb3 | Malat1 | Stmn2 | Fgf15 | Gap43 | Xist | Sncg | Hmgb2 | Top2a | Meg3 |
| --- | --- | --- | --- | --- | --- | --- | --- | --- | --- | --- |
| AC | 1.0 | NaN | 1.0 | 0.0 | 1.0 | NaN | NaN | NaN | 0.0 | 1.0 |
| Cones | NaN | 1.0 | 0.0 | NaN | NaN | NaN | NaN | NaN | NaN | NaN |
| HC | 1.0 | NaN | 1.0 | NaN | NaN | NaN | 0.0 | 1.0 | NaN | NaN |
| NB1 | 1.0 | NaN | NaN | NaN | NaN | NaN | 0.0 | 1.0 | 1.0 | 0.0 |
| NB2 | 1.0 | NaN | 1.0 | NaN | NaN | 0.0 | 0.0 | 1.0 | NaN | 1.0 |
| RGC | 1.0 | NaN | 1.0 | 0.0 | 1.0 | 0.0 | 1.0 | 1.0 | 0.0 | 1.0 |
| RPC | NaN | 0.0 | 0.0 | 1.0 | 0.0 | 0.0 | 0.0 | 1.0 | 1.0 | 0.0 |

In [79]:

```
dorothea_db = pd.read_csv("dorothea_mouse_tfs.csv")
dorothea_db.columns = dorothea_db.columns[:-1].to_list() + ['sign']
dorothea_db.head()
```

Out[79]:

|  | tf | confidence | target | sign |
| --- | --- | --- | --- | --- |
| 0 | 4932411N23Rik | E | Smad4 | 1 |
| 1 | 4932411N23Rik | E | 0610030E20Rik | 1 |
| 2 | 4932411N23Rik | E | 1700017N19Rik | 1 |
| 3 | 4932411N23Rik | E | 4931428F04Rik | 1 |
| 4 | 4932411N23Rik | E | 4932438A13Rik | 1 |

In [80]:

```
def in_db(frame: pd.DataFrame, db: pd.DataFrame):
    is_tf = frame.columns.isin(db.tf)
    is_target = frame.columns.isin(db.target)
    in_db = pd.Series(
        fn.reduce(np.logical_or, [is_tf, is_target]), 
        index=frame.columns
    )
    return in_db
```

In [81]:

```
in_db(meta_boot, dorothea_db).mean()
```

Out[81]:

```
0.8987878787878788
```

In [82]:

```
in_db(meta_boot[marker_genes], dorothea_db).mean()
```

Out[82]:

```
1.0
```

In [83]:

```
meta_boot[null_var_genes].columns[~in_db(meta_boot[null_var_genes], dorothea_db)]
```

Out[83]:

```
Index(['AY036118', 'Gm42418', 'Mum1'], dtype='object')
```

In [87]:

```
print(meta_boot.shape)
meta_boot_db = meta_boot[meta_boot.columns[in_db(meta_boot, dorothea_db)]]
print(meta_boot_db.shape)
meta_boot_db.iloc[:, :10]
```

```
(7, 1650)
(7, 1483)
```

Out[87]:

|  | Tubb3 | Stmn2 | Fgf15 | Gap43 | Sncg | Hmgb2 | Top2a | Ccnd1 | Tubb2b | Ebf1 |
| --- | --- | --- | --- | --- | --- | --- | --- | --- | --- | --- |
| AC | 1.0 | 1.0 | 0.0 | 1.0 | NaN | NaN | 0.0 | 0.0 | 1.0 | 0.0 |
| Cones | NaN | 0.0 | NaN | NaN | NaN | NaN | NaN | NaN | NaN | NaN |
| HC | 1.0 | 1.0 | NaN | NaN | 0.0 | 1.0 | NaN | NaN | 1.0 | NaN |
| NB1 | 1.0 | NaN | NaN | NaN | 0.0 | 1.0 | 1.0 | NaN | 1.0 | 0.0 |
| NB2 | 1.0 | 1.0 | NaN | NaN | 0.0 | 1.0 | NaN | 0.0 | 1.0 | 0.0 |
| RGC | 1.0 | 1.0 | 0.0 | 1.0 | 1.0 | 1.0 | 0.0 | 0.0 | 1.0 | 1.0 |
| RPC | NaN | 0.0 | 1.0 | 0.0 | 0.0 | 1.0 | 1.0 | 1.0 | 1.0 | 0.0 |

In [88]:

```
meta_boot_db.T.isna().mean()
```

Out[88]:

```
AC       0.348618
Cones    0.734322
HC       0.772084
NB1      0.526635
NB2      0.554956
RGC      0.234659
RPC      0.127444
dtype: float64
```

In [89]:

```
meta_boot_db.isna().mean().mean()
```

Out[89]:

```
0.47124554474520763
```

In [90]:

```
import networkx as nx
from grn import *
```

In [91]:

```
dorothea_db.confidence.unique()
```

Out[91]:

```
array(['E', 'D', 'B', 'A', 'C'], dtype=object)
```

In [93]:

```
# The whole grn is weakly connected if we consider all confidence levels
whole_grn = df_to_graph(dorothea_db.query("confidence != 'E' & confidence != 'D'"))
induced_grn = nx.induced_subgraph(whole_grn, meta_boot_db)
len(whole_grn), len(induced_grn)
```

Out[93]:

```
(5201, 622)
```

In [94]:

```
core_tf_nw = extract_largest_scc(whole_grn)
len(core_tf_nw)
```

Out[94]:

```
157
```

In [95]:

```
prior_grn = (
    nx.induced_subgraph(
        whole_grn, 
        set(core_tf_nw).union(
            meta_boot_db.columns
        )
    )
)
len(prior_grn)
```

Out[95]:

```
749
```

In [96]:

```
nx.is_weakly_connected(prior_grn)
```

Out[96]:

```
False
```

In [97]:

```
pkn_biggest_wcc = nx.subgraph(prior_grn, max(nx.weakly_connected_components(prior_grn), key=len))
len(pkn_biggest_wcc)
```

Out[97]:

```
644
```

In [98]:

```
all(i in pkn_biggest_wcc for i in core_tf_nw)
```

Out[98]:

```
True
```

In [100]:

```
marker_genes.isin(prior_grn).mean()
```

Out[100]:

```
0.7272727272727273
```

In [99]:

```
marker_genes.isin(pkn_biggest_wcc).mean()
```

Out[99]:

```
0.6363636363636364
```

In [101]:

```
print("\t".join(["Cell", "pTotal", "pBiggestWCC"]))
for pheno, genes in states_and_markers.items():
    if pheno in meta_boot_db.index:
        print("\t".join(
            (lambda __: f"{100*__:.2f}" if isinstance(__, float) else __)(_)
            for _ in
            [pheno, pd.Index(genes).isin(whole_grn).mean(), pd.Index(genes).isin(pkn_biggest_wcc).mean()]
        ))
```

```
Cell	pTotal	pBiggestWCC
RPC	100.00	100.00
NB1	100.00	80.00
NB2	33.33	33.33
RGC	50.00	50.00
AC	50.00	50.00
HC	100.00	100.00
Cones	75.00	50.00
```

In [102]:

```
import bonesis
```

In [103]:

```
def df_to_bonesis_data(df: pd.DataFrame):
    data = {}
    for config, genes in df.iterrows():
        data.update({config: genes.dropna().to_dict()})
    return data
```

In [105]:

```
data_meta_sum = df_to_bonesis_data(meta_boot_db) 
data_meta_sum.keys()
```

Out[105]:

```
dict_keys(['AC', 'Cones', 'HC', 'NB1', 'NB2', 'RGC', 'RPC'])
```

In [106]:

```
pkn = bonesis.domains.InfluenceGraph(
    prior_grn, maxclause=8, allow_skipping_nodes=True, canonic=False
)
```

In [107]:

```
bo = bonesis.BoNesis(pkn, data_meta_sum)
bo
```

Out[107]:

```
<bonesis.BoNesis at 0x7efd9f454d90>
```

In [108]:

```
~bo.obs("RPC") >= ~bo.obs("NB1") >= ~bo.obs("NB2")
~bo.obs("NB2") >= bo.fixed(~bo.obs("Cones"))
~bo.obs("NB2") >= bo.fixed(~bo.obs("RGC"))
~bo.obs("NB2") >= bo.fixed(~bo.obs("AC"))
~bo.obs("NB2") >= bo.fixed(~bo.obs("HC"))
None
```

In [109]:

```
import datetime
from pathlib import Path as path
from scboolseq.utils import Timer
```

In [110]:

```
date = datetime.datetime.now()
bo.maximize_nodes()
bo.maximize_strong_constants()
view = bonesis.NonStrongConstantNodesView(bo, mode="optN")
view.standalone(
    output_filename=f"optim_dorothea_v2_bootstrap:_{date.strftime('%Y-%m-%d')}.sh"
)
print("Standalone shell file written", flush=True)

kept_nodes = {}
with Timer("Optimizing NonStrongConstantNodesView(bo, mode='optN')"):
    for kept_nodes in view:
        break

_replica_grn = nx.subgraph(prior_grn, kept_nodes)
```

```
Standalone shell file written
Grounding...done in 1.4s
<class 'bonesis0.gil_utils.BGIteratorPersistent'>
Optimizing NonStrongConstantNodesView(bo, mode='optN'): 1360.76223
```

In [111]:

```
len(kept_nodes)
```

Out[111]:

```
177
```

In [112]:

```
marker_genes.isin(_replica_grn).mean()
```

Out[112]:

```
0.3181818181818182
```

In [62]:

```
bonesis.InfluenceGraph(_replica_grn)
```

```
# computing graph layout...
```

Out[62]:

In [113]:

```
EXPORT = True

if EXPORT:
    nx.nx_pydot.write_dot(extract_largest_scc(bonesis.InfluenceGraph(_replica_grn).as_nx), "bootrstrap_grn_scc_v1.dot")
    import json
    with open("bootstrap_grn_optimized_v1.json", "w") as f:
        json.dump(nx.node_link_data(_replica_grn), f)
    
    with open("bootstrap_pkn_bonesis_obj_optimized_v1.json", "w") as f:
        json.dump(nx.node_link_data(bonesis.InfluenceGraph(_replica_grn).as_nx), f)
    print("Exported")
else:
    print("Not exported")
```

```
Exported
```

In [ ]:

```

```
