# Supplementary material for: scBoolSeq: Linking scRNA-seq statistics and Boolean dynamics
Source: PLoS Comput Biol. 2024 Jul 8;20(7):e1011620. doi: 10.1371/journal.pcbi.1011620 (PMC11257695; doi:10.1371/journal.pcbi.1011620)
Supplement: S1 Notebooks — The notebooks are provided as static HTML files, and Boolean networks as textual files in BoolNet format. See the Data availability statement for links to executable notebooks and code. (ZIP) [file pcbi.1011620.s001.zip › Basic scBoolSeq usage.html]

Basic scBoolSeq usage


# scBoolSeq API demonstration¶

This notebook demonstrates the basic features of scBoolSeq: scRNA-Seq data binarization and synthetic generation.

Note that scBoolSeq also comes with a command line interface, see https://github.com/bnediction/scBoolSeq.

In [1]:

```
import pandas as pd
from scboolseq import scBoolSeq
```

### Retrieve an example dataset¶

The demonstration will be performed on the scRNA-Seq datasets from XXX.

In [2]:

```
!test -f data_Nestorowa.tsv.gz || curl -fOL \
    https://github.com/pinellolab/STREAM/raw/master/stream/tests/datasets/Nestorowa_2016/data_Nestorowa.tsv.gz
```

**Important**: `scBoolSeq` expects the data to be formatted as follows: columns representing genes and rows representing cells/samples (sc/bulk RNA-Seq).

In [3]:

```
nestorowa = pd.read_csv("data_Nestorowa.tsv.gz", compression="gzip", sep="\t", index_col=0).T
nestorowa.head()
```

Out[3]:

|  | Clec1b | Kdm3a | Coro2b | 8430408G22Rik | Clec9a | Phf6 | Usp14 | Tmem167b | Kbtbd7 | Rag2 | ... | Zfp438 | Rab18 | Mzb1 | B4galt6 | Rnf125 | Impact | Taf4b | Zfp521 | Hrh4 | Psma8 |
| --- | --- | --- | --- | --- | --- | --- | --- | --- | --- | --- | --- | --- | --- | --- | --- | --- | --- | --- | --- | --- | --- |
| HSPC\_025 | 0.0 | 4.891604 | 1.426148 | 0.0 | 0.0 | 2.599758 | 2.954035 | 6.357369 | 2.129140 | 1.426148 | ... | 1.426148 | 9.660368 | 1.426148 | 1.426148 | 2.12914 | 8.177546 | 1.426148 | 1.426148 | 0.0 | 7.869409 |
| HSPC\_031 | 0.0 | 6.877725 | 0.000000 | 0.0 | 0.0 | 2.423483 | 1.804914 | 0.000000 | 0.000000 | 0.000000 | ... | 0.000000 | 0.699126 | 0.000000 | 6.562672 | 0.00000 | 5.439604 | 0.699126 | 0.000000 | 0.0 | 0.000000 |
| HSPC\_037 | 0.0 | 0.000000 | 6.913384 | 0.0 | 0.0 | 2.051659 | 8.265465 | 0.000000 | 1.363402 | 0.000000 | ... | 1.363402 | 8.885311 | 0.000000 | 1.363402 | 0.00000 | 8.068215 | 0.000000 | 2.051659 | 0.0 | 1.363402 |
| LT-HSC\_001 | 0.0 | 0.000000 | 8.178374 | 0.0 | 0.0 | 6.419817 | 3.453502 | 2.579528 | 2.579528 | 0.000000 | ... | 2.579528 | 6.501342 | 4.947883 | 0.000000 | 0.00000 | 0.000000 | 2.579528 | 8.178374 | 0.0 | 2.579528 |
| HSPC\_001 | 0.0 | 0.000000 | 9.475577 | 0.0 | 0.0 | 7.733370 | 1.478900 | 0.000000 | 10.045601 | 0.532906 | ... | 0.000000 | 1.693409 | 7.975432 | 8.561045 | 0.00000 | 6.539920 | 0.532906 | 0.000000 | 0.0 | 0.532906 |

5 rows × 4768 columns

## Instantiation¶

In [4]:

```
scbool = scBoolSeq()
scbool
```

Out[4]:

```
scBoolSeqBinarizer()
```

**In a Jupyter environment, please rerun this cell to show the HTML representation or trust the notebook.   
On GitHub, the HTML representation is unable to render, please try loading this page with nbviewer.org.**

scBoolSeqBinarizer

```
scBoolSeqBinarizer()
```

### Binarization¶

The binarization requires learning the distribution of RNA pseudocounts for each gene, which is performed by the `fit()` method:

In [5]:

```
%time scbool.fit(nestorowa)
```

```
Computing bimodality index for 2303/4768 genes
Computing bimodality index for 2934/4768 genes
```

```
/opt/conda/lib/python3.10/site-packages/numpy/core/fromnumeric.py:57: FutureWarning: 'DataFrame.swapaxes' is deprecated and will be removed in a future version. Please use 'DataFrame.transpose' instead.
  return bound(*args, **kwds)
```

```
CPU times: user 3min 15s, sys: 2.06 s, total: 3min 18s
Wall time: 58.9 s
```

Out[5]:

```
scBoolSeqBinarizer()
```

**In a Jupyter environment, please rerun this cell to show the HTML representation or trust the notebook.   
On GitHub, the HTML representation is unable to render, please try loading this page with nbviewer.org.**

scBoolSeqBinarizer

```
scBoolSeqBinarizer()
```

Internally, the learned features are stored in a `criteria` table, which can be accessed like this:

In [6]:

```
scbool.criteria_[['Category', *scbool.criteria_]].head(10)
```

Out[6]:

|  | Category | Mean | MeanNZ | Median | MedianNZ | GeometricMean | HarmonicMean | Variance | VarianceNZ | DropOutRate | Amplitude | Dip | Kurtosis | Skewness | DenPeak | BI | Category |
| --- | --- | --- | --- | --- | --- | --- | --- | --- | --- | --- | --- | --- | --- | --- | --- | --- | --- |
| Clec1b | ZeroInf | 0.188285 | 1.520978 | 0.000000 | 0.968776 | 1.077120 | 0.843836 | 0.579440 | 2.653752 | 0.876208 | 8.852181 | 0.358107 | 54.017736 | 6.716474 | -0.000128 | 0.000000 | ZeroInf |
| Kdm3a | Bimodal | 2.593177 | 3.847940 | 1.268040 | 2.737412 | 2.682239 | 1.747482 | 8.687337 | 8.062633 | 0.326087 | 10.126676 | 0.000000 | -0.784019 | 0.863438 | 0.303398 | 2.401623 | Bimodal |
| Coro2b | ZeroInf | 0.814759 | 2.383819 | 0.000000 | 1.290666 | 1.586378 | 1.149780 | 3.110739 | 5.361032 | 0.658213 | 9.475577 | 0.000000 | 7.061604 | 2.771571 | 0.003072 | 0.000000 | ZeroInf |
| 8430408G22Rik | ZeroInf | 0.345910 | 2.983472 | 0.000000 | 1.449779 | 1.845045 | 1.214593 | 1.852900 | 8.112175 | 0.884058 | 9.067857 | 0.684454 | 21.729044 | 4.708367 | 0.003788 | 0.000000 | ZeroInf |
| Clec9a | ZeroInf | 0.078488 | 2.280293 | 0.000000 | 1.229896 | 1.525787 | 1.148000 | 0.372653 | 5.805785 | 0.965580 | 9.614233 | 1.000000 | 140.089285 | 11.195517 | 0.000308 | 0.000000 | ZeroInf |
| Phf6 | Bimodal | 4.846453 | 5.025501 | 4.778527 | 5.051362 | 4.104554 | 3.106348 | 8.034313 | 7.431326 | 0.035628 | 10.135226 | 0.000000 | -1.389024 | -0.002268 | 2.033821 | 1.989131 | Bimodal |
| Usp14 | Bimodal | 6.061999 | 6.109964 | 7.140887 | 7.170392 | 5.283258 | 4.241799 | 7.524603 | 7.291078 | 0.007850 | 11.088750 | 0.000000 | -1.224987 | -0.450551 | 8.231397 | 2.208317 | Bimodal |
| Tmem167b | Bimodal | 2.090655 | 3.448331 | 0.924808 | 2.027150 | 2.356898 | 1.589141 | 7.572099 | 7.807721 | 0.393720 | 9.486826 | 0.000000 | 0.093023 | 1.246701 | 0.115315 | 2.426544 | Bimodal |
| Kbtbd7 | ZeroInf | 1.255786 | 2.928988 | 0.000000 | 1.671472 | 1.961379 | 1.349735 | 5.089084 | 6.968960 | 0.571256 | 10.910051 | 0.000000 | 3.577214 | 2.131193 | 0.004064 | 0.000000 | ZeroInf |
| Rag2 | ZeroInf | 1.041198 | 1.928663 | 0.548416 | 1.274551 | 1.390515 | 1.097732 | 3.036219 | 3.912518 | 0.460145 | 10.348297 | 0.000000 | 9.080962 | 2.910103 | 0.007790 | 0.000000 | ZeroInf |

The actual binarization is performed using the `.binarize` method, and takes as argument the dataset to binarize, which can be identifical to the reference dataset used for learning criteria:

In [7]:

```
%time nestorowa_binarized = scbool.binarize(nestorowa)
```

```
/opt/conda/lib/python3.10/site-packages/numpy/core/fromnumeric.py:57: FutureWarning: 'DataFrame.swapaxes' is deprecated and will be removed in a future version. Please use 'DataFrame.transpose' instead.
  return bound(*args, **kwds)
```

```
CPU times: user 3.22 s, sys: 18.1 ms, total: 3.24 s
Wall time: 3.24 s
```

The method returns a Pandas dataframe replacing the RNA log pseudocounts with `0`, `1`, or `NaN`:

In [8]:

```
nestorowa_binarized.head()
```

Out[8]:

|  | Clec1b | Kdm3a | Coro2b | 8430408G22Rik | Clec9a | Phf6 | Usp14 | Tmem167b | Kbtbd7 | Rag2 | ... | Zfp438 | Rab18 | Mzb1 | B4galt6 | Rnf125 | Impact | Taf4b | Zfp521 | Hrh4 | Psma8 |
| --- | --- | --- | --- | --- | --- | --- | --- | --- | --- | --- | --- | --- | --- | --- | --- | --- | --- | --- | --- | --- | --- |
| HSPC\_025 | NaN | 1.0 | 1.0 | NaN | NaN | 0.0 | 0.0 | 1.0 | 1.0 | 1.0 | ... | 1.0 | 1.0 | 1.0 | 0.0 | 0.0 | 1.0 | NaN | 1.0 | NaN | 1.0 |
| HSPC\_031 | NaN | 1.0 | NaN | NaN | NaN | 0.0 | 0.0 | 0.0 | NaN | NaN | ... | NaN | 0.0 | NaN | 1.0 | 0.0 | NaN | NaN | NaN | NaN | NaN |
| HSPC\_037 | NaN | 0.0 | 1.0 | NaN | NaN | 0.0 | 1.0 | 0.0 | 1.0 | NaN | ... | 1.0 | 1.0 | NaN | 0.0 | 0.0 | 1.0 | NaN | 1.0 | NaN | 1.0 |
| LT-HSC\_001 | NaN | 0.0 | 1.0 | NaN | NaN | 1.0 | 0.0 | NaN | 1.0 | NaN | ... | 1.0 | 1.0 | 1.0 | 0.0 | 0.0 | 0.0 | NaN | 1.0 | NaN | 1.0 |
| HSPC\_001 | NaN | 0.0 | 1.0 | NaN | NaN | 1.0 | 0.0 | 0.0 | 1.0 | 1.0 | ... | NaN | 0.0 | 1.0 | 1.0 | 0.0 | 1.0 | NaN | NaN | NaN | 1.0 |

5 rows × 4768 columns

### Synthetic generation¶

The synthetic generation is performed from fully determined Boolean states, given as a Pandas dataframe.

For this example, we simply reuse the binarized data, where we replace the `NaN` entries with random Boolean values:

In [9]:

```
from scboolseq.simulation import random_nan_binariser
fully_bin = random_nan_binariser(nestorowa_binarized)
fully_bin.head()
```

Out[9]:

|  | Clec1b | Kdm3a | Coro2b | 8430408G22Rik | Clec9a | Phf6 | Usp14 | Tmem167b | Kbtbd7 | Rag2 | ... | Zfp438 | Rab18 | Mzb1 | B4galt6 | Rnf125 | Impact | Taf4b | Zfp521 | Hrh4 | Psma8 |
| --- | --- | --- | --- | --- | --- | --- | --- | --- | --- | --- | --- | --- | --- | --- | --- | --- | --- | --- | --- | --- | --- |
| HSPC\_025 | 1.0 | 1.0 | 1.0 | 0.0 | 0.0 | 0.0 | 0.0 | 1.0 | 1.0 | 1.0 | ... | 1.0 | 1.0 | 1.0 | 0.0 | 0.0 | 1.0 | 0.0 | 1.0 | 1.0 | 1.0 |
| HSPC\_031 | 0.0 | 1.0 | 0.0 | 1.0 | 1.0 | 0.0 | 0.0 | 0.0 | 0.0 | 0.0 | ... | 1.0 | 0.0 | 0.0 | 1.0 | 0.0 | 0.0 | 0.0 | 1.0 | 0.0 | 0.0 |
| HSPC\_037 | 0.0 | 0.0 | 1.0 | 1.0 | 0.0 | 0.0 | 1.0 | 0.0 | 1.0 | 1.0 | ... | 1.0 | 1.0 | 0.0 | 0.0 | 0.0 | 1.0 | 0.0 | 1.0 | 0.0 | 1.0 |
| LT-HSC\_001 | 1.0 | 0.0 | 1.0 | 1.0 | 0.0 | 1.0 | 0.0 | 1.0 | 1.0 | 1.0 | ... | 1.0 | 1.0 | 1.0 | 0.0 | 0.0 | 0.0 | 0.0 | 1.0 | 0.0 | 1.0 |
| HSPC\_001 | 0.0 | 0.0 | 1.0 | 1.0 | 1.0 | 1.0 | 0.0 | 0.0 | 1.0 | 1.0 | ... | 0.0 | 0.0 | 1.0 | 1.0 | 0.0 | 1.0 | 1.0 | 1.0 | 1.0 | 1.0 |

5 rows × 4768 columns

For this example, we generate synthetic RNA-Seq only for a subset of genes and cells:

In [10]:

```
to_simulate = fully_bin.iloc[:100, :10]
to_simulate
```

Out[10]:

|  | Clec1b | Kdm3a | Coro2b | 8430408G22Rik | Clec9a | Phf6 | Usp14 | Tmem167b | Kbtbd7 | Rag2 |
| --- | --- | --- | --- | --- | --- | --- | --- | --- | --- | --- |
| HSPC\_025 | 1.0 | 1.0 | 1.0 | 0.0 | 0.0 | 0.0 | 0.0 | 1.0 | 1.0 | 1.0 |
| HSPC\_031 | 0.0 | 1.0 | 0.0 | 1.0 | 1.0 | 0.0 | 0.0 | 0.0 | 0.0 | 0.0 |
| HSPC\_037 | 0.0 | 0.0 | 1.0 | 1.0 | 0.0 | 0.0 | 1.0 | 0.0 | 1.0 | 1.0 |
| LT-HSC\_001 | 1.0 | 0.0 | 1.0 | 1.0 | 0.0 | 1.0 | 0.0 | 1.0 | 1.0 | 1.0 |
| HSPC\_001 | 0.0 | 0.0 | 1.0 | 1.0 | 1.0 | 1.0 | 0.0 | 0.0 | 1.0 | 1.0 |
| ... | ... | ... | ... | ... | ... | ... | ... | ... | ... | ... |
| LT-HSC\_014 | 1.0 | 0.0 | 1.0 | 0.0 | 0.0 | 1.0 | 1.0 | 1.0 | 1.0 | 1.0 |
| HSPC\_044 | 1.0 | 1.0 | 1.0 | 1.0 | 1.0 | 1.0 | 0.0 | 0.0 | 1.0 | 1.0 |
| HSPC\_051 | 0.0 | 1.0 | 1.0 | 1.0 | 1.0 | 1.0 | 0.0 | 0.0 | 1.0 | 0.0 |
| HSPC\_057 | 1.0 | 1.0 | 0.0 | 0.0 | 1.0 | 0.0 | 0.0 | 0.0 | 1.0 | 1.0 |
| HSPC\_063 | 0.0 | 0.0 | 0.0 | 1.0 | 0.0 | 0.0 | 1.0 | 1.0 | 1.0 | 0.0 |

100 rows × 10 columns

In [11]:

```
synthetic_rna = scbool.sample_counts(to_simulate, n_samples_per_state=3, random_state=1234)
synthetic_rna.head()
```

```
/opt/conda/lib/python3.10/site-packages/numpy/core/fromnumeric.py:57: FutureWarning: 'DataFrame.swapaxes' is deprecated and will be removed in a future version. Please use 'DataFrame.transpose' instead.
  return bound(*args, **kwds)
```

Out[11]:

|  | Clec1b | Kdm3a | Coro2b | 8430408G22Rik | Clec9a | Phf6 | Usp14 | Tmem167b | Kbtbd7 | Rag2 |
| --- | --- | --- | --- | --- | --- | --- | --- | --- | --- | --- |
| HSPC\_025 | 13.277362 | 10.762286 | 11.311510 | 4.444016 | 0.774097 | 1.913995 | 2.729077 | 7.014069 | 8.220823 | 8.613674 |
| HSPC\_031 | 4.438015 | 10.722209 | 5.281329 | 10.013828 | 11.953065 | 3.310323 | 2.124285 | 2.668958 | 5.384847 | 3.979046 |
| HSPC\_037 | 3.336958 | 3.263866 | 7.564283 | 8.485172 | 3.184775 | 3.831573 | 7.521112 | 3.066981 | 9.267410 | 8.522187 |
| LT-HSC\_001 | 15.005001 | 4.767465 | 10.358621 | 9.710105 | 1.888307 | 6.822719 | 3.484982 | 9.760267 | 9.706970 | 7.911266 |
| HSPC\_001 | 5.586974 | 4.393809 | 7.942651 | 9.405537 | 8.618470 | 6.227695 | 0.000000 | 0.000000 | 9.664364 | 8.204868 |

In [12]:

```
synthetic_rna.shape
```

Out[12]:

```
(300, 10)
```

In [ ]:

```

```
