# Supplementary material for: scBoolSeq: Linking scRNA-seq statistics and Boolean dynamics
Source: PLoS Comput Biol. 2024 Jul 8;20(7):e1011620. doi: 10.1371/journal.pcbi.1011620 (PMC11257695; doi:10.1371/journal.pcbi.1011620)
Supplement: S1 Notebooks — The notebooks are provided as static HTML files, and Boolean networks as textual files in BoolNet format. See the Data availability statement for links to executable notebooks and code. (ZIP) [file pcbi.1011620.s001.zip › BoolODE_comparison.html]

BoolODE\_comparison


# Comparing the statistics of synthetic scRNA-Seq data generated by BoolODE and scBoolSeq¶

IN this notebook we showcase how scBoolSeq produces synthetic scRNA-Seq data which better fits the mean ~ variance and mean ~ dropout rate relationships of real datasets.

In [1]:

```
from IPython.display import display, HTML
display(HTML("<style>.container { width:100% !important; }</style>"))
import warnings
warnings.filterwarnings("ignore")
```

In [2]:

```
import matplotlib.pyplot as plt
import numpy as np
import pandas as pd
from sklearn.decomposition import PCA
import statsmodels.api as sm
from plotnine import *

import scboolseq
from scboolseq import scBoolSeq
```

In [3]:

```
%matplotlib inline
```

In [4]:

```
%ls data_filtered_vargenes/*csv
```

```
data_filtered_vargenes/GSE122466_Retina.csv
data_filtered_vargenes/GSE122466_Retina_vargenes_batch1.csv
data_filtered_vargenes/GSE130973_human_skin.csv
data_filtered_vargenes/GSE156234_Macrophages.csv
data_filtered_vargenes/GSE60361_mouse_brain.csv
data_filtered_vargenes/GSE81682_Hematopoiesis.csv
```

In [5]:

```
reference = raw_reference = pd.read_csv("data_filtered_vargenes/GSE122466_Retina_vargenes_batch1.csv", index_col=0)
raw_reference.head()
```

Out[5]:

|  | Tubb3 | Malat1 | Stmn2 | Fgf15 | Gap43 | Xist | Sncg | Hmgb2 | Top2a | Meg3 | ... | Prdm13 | Kif14 | Rpl24 | Etfb | Cd320 | Fam98b | Odf2 | Fbxo36 | Rbp4 | Pou4f2 |
| --- | --- | --- | --- | --- | --- | --- | --- | --- | --- | --- | --- | --- | --- | --- | --- | --- | --- | --- | --- | --- | --- |
| Lane1\_AAACCTGAGATGTCGG | 0.000000 | 14.687273 | 9.280150 | 10.278990 | 0.000000 | 0.000000 | 8.282469 | 10.863565 | 8.282469 | 8.282469 | ... | 0.0 | 0.0 | 11.737682 | 0.000000 | 0.000000 | 0.000000 | 0.000000 | 0.0 | 0.0 | 0.0 |
| Lane1\_AAACCTGCAATCCAAC | 0.000000 | 14.568977 | 0.000000 | 10.200911 | 0.000000 | 0.000000 | 0.000000 | 10.200911 | 0.000000 | 0.000000 | ... | 0.0 | 0.0 | 10.615642 | 9.616560 | 0.000000 | 8.618397 | 0.000000 | 0.0 | 0.0 | 0.0 |
| Lane1\_AAACCTGGTTCCTCCA | 12.822128 | 16.422850 | 11.681013 | 0.000000 | 12.722607 | 0.000000 | 12.615708 | 0.000000 | 0.000000 | 11.237565 | ... | 0.0 | 0.0 | 9.402599 | 6.607977 | 0.000000 | 6.607977 | 6.607977 | 0.0 | 0.0 | 0.0 |
| Lane1\_AAACCTGTCCAATGGT | 12.601411 | 16.106194 | 10.514703 | 0.000000 | 8.517658 | 0.000000 | 8.517658 | 10.099994 | 0.000000 | 0.000000 | ... | 0.0 | 0.0 | 10.099994 | 0.000000 | 8.517658 | 0.000000 | 0.000000 | 0.0 | 0.0 | 0.0 |
| Lane1\_AAACGGGAGGCAATTA | 0.000000 | 14.858160 | 0.000000 | 12.375060 | 7.766719 | 11.082672 | 6.773328 | 11.889742 | 11.461031 | 7.766719 | ... | 0.0 | 0.0 | 11.284220 | 6.773328 | 0.000000 | 8.349471 | 6.773328 | 0.0 | 0.0 | 0.0 |

5 rows × 1650 columns

In [6]:

```
#"boolean_trace.csv"
trace = pd.read_csv("boolode_gsd_trace.csv", index_col=0)
print(trace.shape)
configurations = trace.groupby(trace.index).agg(lambda x: x.mode())
configurations
```

```
(700, 19)
```

Out[6]:

|  | AMH | CBX2 | CTNNB1 | DHH | DKK1 | DMRT1 | FGF9 | FOXL2 | GATA4 | NR0B1 | NR5A1 | PGD2 | RSPO1 | SOX9 | SRY | UGR | WNT4 | WT1mKTS | WT1pKTS |
| --- | --- | --- | --- | --- | --- | --- | --- | --- | --- | --- | --- | --- | --- | --- | --- | --- | --- | --- | --- |
| Sertoli | 1 | 0 | 0 | 1 | 1 | 1 | 1 | 0 | 1 | 0 | 1 | 1 | 0 | 1 | 1 | 0 | 0 | 1 | 1 |
| granulosa | 0 | 0 | 1 | 0 | 0 | 0 | 0 | 1 | 1 | 1 | 0 | 0 | 1 | 0 | 0 | 0 | 1 | 1 | 0 |
| start | 1 | 0 | 1 | 1 | 0 | 1 | 0 | 0 | 1 | 1 | 1 | 1 | 1 | 1 | 0 | 1 | 1 | 1 | 1 |
| t\_Sertoli\_1 | 1 | 0 | 0 | 1 | 1 | 1 | 0 | 0 | 1 | 0 | 1 | 1 | 1 | 1 | 0 | 1 | 1 | 1 | 1 |
| t\_Sertoli\_2 | 1 | 0 | 0 | 1 | 1 | 1 | 1 | 0 | 1 | 0 | 1 | 1 | 1 | 1 | 1 | 1 | 0 | 1 | 1 |
| t\_granulosa\_1 | 1 | 0 | 1 | 0 | 0 | 0 | 0 | 0 | 1 | 1 | 1 | 1 | 1 | 0 | 0 | 1 | 1 | 1 | 1 |
| t\_granulosa\_2 | 1 | 0 | 1 | 0 | 0 | 0 | 0 | 1 | 1 | 1 | 0 | 1 | 1 | 0 | 0 | 1 | 1 | 1 | 0 |

In [7]:

```
configurations.loc[configurations.UGR == 0, :].index.unique()
```

Out[7]:

```
Index(['Sertoli', 'granulosa'], dtype='object')
```

In [8]:

```
%time scbool = scBoolSeq().fit(reference)
```

```
Computing bimodality index for 853/1650 genes
Computing bimodality index for 43/1650 genes
CPU times: user 54.5 s, sys: 1.6 s, total: 56.1 s
Wall time: 11.3 s
```

In [9]:

```
%%time
a, b = .3, .7

SEED = 1234
rng = np.random.default_rng(SEED)

fig, ax = plt.subplots(nrows=1, ncols=2, figsize=(15,5))
ax[0].hist(scbool.criteria_.DropOutRate.values, density=True)
ax[1].hist(rng.beta(a, b, size=1_000), density=True)
new_data =  scbool.synthetic_rna_from_boolean_states(
    trace, 
    n_samples_per_state=10,
)
new_data_beta =  scbool.synthetic_rna_from_boolean_states(
    trace, 
    n_samples_per_state=10, 
    dropout_rates=rng.beta(a, b, size=trace.shape[1])
)
new_data_constant = scbool.synthetic_rna_from_boolean_states(
    trace, 
    n_samples_per_state=10, 
    dropout_rates=np.array([0.5 for _ in range(trace.shape[1])]),
    dropout_mode = "uniform"
)
new_data_no_dor= scbool.synthetic_rna_from_boolean_states(
    trace, 
    n_samples_per_state=10, 
    dropout_mode = None
)
```

```
CPU times: user 367 ms, sys: 352 ms, total: 719 ms
Wall time: 1.49 s
```

In [10]:

```
%%time
our_scbool_natural_dor = scBoolSeq().fit(new_data)
our_scbool_beta_dropout = scBoolSeq().fit(new_data_beta)
our_scbool_constant_dropout = scBoolSeq().fit(new_data_constant)
our_scbool_no_dor = scBoolSeq().fit(new_data_no_dor)
```

```
Computing bimodality index for 10/19 genes
Computing bimodality index for 4/19 genes
Computing bimodality index for 8/19 genes
Computing bimodality index for 4/19 genes
Computing bimodality index for 15/19 genes
Computing bimodality index for 4/19 genes
Computing bimodality index for 9/19 genes
Computing bimodality index for 4/19 genes
CPU times: user 1min 20s, sys: 1min 39s, total: 3min
Wall time: 11.9 s
```

In [16]:

```
!ls boolODE_data/
```

```
boolode1_dor_30_70.csv  boolode1_dor_90_30.csv  boolode1_no_dor.csv
boolode1_dor_50_50.csv  boolode1_dor_90_50.csv
```

In [18]:

```
from pathlib import Path as path
from sklearn.utils import Bunch
```

In [19]:

```
%%time
boolode_dataframes = Bunch(**{
    file.name.replace(file.suffix, ''): pd.read_csv(file, index_col=0).T
    for file in path("./boolODE_data/").resolve().glob("boolode*csv")
})
boolode_dataframes.keys()
```

```
CPU times: user 353 ms, sys: 12.3 ms, total: 365 ms
Wall time: 364 ms
```

Out[19]:

```
dict_keys(['boolode1_dor_50_50', 'boolode1_dor_30_70', 'boolode1_dor_90_50', 'boolode1_dor_90_30', 'boolode1_no_dor'])
```

In [20]:

```
%%time 

boolode_scbool = Bunch(**{
    name: scBoolSeq().fit(8*_data) # The 8 is needed to match the amplitude of mean values in real data
    for name, _data in boolode_dataframes.items()
})
```

```
Computing bimodality index for 17/19 genes
Computing bimodality index for 16/19 genes
Computing bimodality index for 17/19 genes
Computing bimodality index for 15/19 genes
Computing bimodality index for 15/19 genes
Computing bimodality index for 16/19 genes
Computing bimodality index for 17/19 genes
Computing bimodality index for 16/19 genes
Computing bimodality index for 15/19 genes
Computing bimodality index for 15/19 genes
CPU times: user 15.9 s, sys: 1.18 s, total: 17.1 s
Wall time: 2.46 s
```

In [21]:

```
to_consider = [
    'boolode1_dor_30_70',
    'boolode1_dor_90_50',
    'boolode1_no_dor',
]
```

In [22]:

```
_cols = "Mean|Variance|DropOutRate".split("|")
N = len(to_consider) + 4
fig, axs = plt.subplots(nrows=N, ncols=2, figsize=(16,16))

for i, name in enumerate(to_consider):
    print(name, i//N, i%2)
    _ax, _ax2 = axs[i + 1]
    _crit_ = boolode_scbool[name].criteria_
    _crit_ = _crit_[_crit_.columns[_crit_.columns != 'Category']]
    _x = _crit_.copy(deep=True)
    _x.plot.scatter(x="Mean", y="DropOutRate", alpha=.7, ax=_ax, s=40)
    _x.plot.scatter(x="Mean", y="Variance", alpha=.7, ax=_ax2, s=40)
    
    _title = (
        "(No dropout)"
        if 'no' in name
        else f"QC(.{name.split('_')[-2]})  DP(.{name.split('_')[-1]})"
    )
    _ax.set_title(f"boolODE {_title}")
    _ax2.set_title(f"boolODE {_title}")
```

```
boolode1_dor_30_70 0 0
boolode1_dor_90_50 0 1
boolode1_no_dor 0 0
```

In [23]:

```
_1, _2 = axs[0]
_t = "Reference Dataset"
_1.set_title(_t); _2.set_title(_t);
_x = scbool.criteria_
_x.plot.scatter(x="Mean", y="DropOutRate", alpha=.7, ax=_1, c="red")
_x.plot.scatter(x="Mean", y="Variance", alpha=.7, ax=_2, c="red")
```

Out[23]:

```
<Axes: title={'center': 'Reference Dataset'}, xlabel='Mean', ylabel='Variance'>
```

In [24]:

```
_1, _2 = axs[-3]
_t = "scBoolSeq reference Dropout"
_1.set_title(_t); _2.set_title(_t);
_x = our_scbool_natural_dor.criteria_
_x.plot.scatter(x="Mean", y="DropOutRate", alpha=.7, ax=_1, c="green", s=40)
_x.plot.scatter(x="Mean", y="Variance", alpha=.7, ax=_2, c="green", s=40)
```

Out[24]:

```
<Axes: title={'center': 'scBoolSeq reference Dropout'}, xlabel='Mean', ylabel='Variance'>
```

In [25]:

```
_1, _2 = axs[-2]
_t = f"scBoolSeq constant Dropout = 0.5"
_1.set_title(_t); _2.set_title(_t);
_x = our_scbool_constant_dropout.criteria_
_x.plot.scatter(x="Mean", y="DropOutRate", alpha=.7, ax=_1, c="green", s=40)
_x.plot.scatter(x="Mean", y="Variance", alpha=.7, ax=_2, c="green", s=40)
```

Out[25]:

```
<Axes: title={'center': 'scBoolSeq constant Dropout = 0.5'}, xlabel='Mean', ylabel='Variance'>
```

In [26]:

```
_1, _2 = axs[-1]
_t = f"scBoolSeq DropOutRate = 0.0"
_1.set_title(_t); _2.set_title(_t);
_x = our_scbool_no_dor.criteria_
_x.plot.scatter(x="Mean", y="DropOutRate", alpha=.7, ax=_1, c="green", s=40)
_x.plot.scatter(x="Mean", y="Variance", alpha=.7, ax=_2, c="green", s=40)
```

Out[26]:

```
<Axes: title={'center': 'scBoolSeq DropOutRate = 0.0'}, xlabel='Mean', ylabel='Variance'>
```

In [27]:

```
for i in range(len(axs)-1):
    for j in range(len(axs[0])):
        axs[i][j].tick_params('x', labelbottom=False)
        axs[i][j].get_xaxis().set_visible(False)
    
for i in range(len(axs)):
    axs[i][1].set_ylim(-5, 75)
    if i > 0:
        axs[i][0].sharey(axs[0][0])
```

In [28]:

```
fig.tight_layout()
```

In [29]:

```
fig
```

Out[29]:

In [ ]:

```
for _format in ['pdf', 'png', 'png', 'svg']:
    fig.savefig(f"comparison.{_format}", format=_format)
```

In [ ]:

```
_x[["Mean", "DropOutRate", "Variance"]].describe()
```

In [ ]:

```
fig.tight_layout()
```

In [ ]:

```
fig
```

In [ ]:

```

```
