# Supplementary material for: scBoolSeq: Linking scRNA-seq statistics and Boolean dynamics
Source: PLoS Comput Biol. 2024 Jul 8;20(7):e1011620. doi: 10.1371/journal.pcbi.1011620 (PMC11257695; doi:10.1371/journal.pcbi.1011620)
Supplement: S1 Notebooks — The notebooks are provided as static HTML files, and Boolean networks as textual files in BoolNet format. See the Data availability statement for links to executable notebooks and code. (ZIP) [file pcbi.1011620.s001.zip › Tutorial_scBoolSeq_scanpy_binarisation.html]

Tutorial\_scBoolSeq\_scanpy\_binarisation


# Demo Notebook: Using `scBoolSeq` with `scanpy` (Binarisation)¶

In this notebook we showcase:

- How to use scanpy, to pre-process scRNA-seq data in
  order to be used with `scBoolSeq`. `scanpy` is a popular package for scRNA-seq data analysis in Python (part of the scverse). This tutorial builds on `scanpy`'s preprocessing and clustering tutorial.
- The integration of `scBoolSeq` with `scanpy`.
- How obtain per-cell, coarse-grained gene activities using `scBoolSeq`.
- How to aggregate these coarse-grained cells to obtain Boolean signatures for cell clusters automatically detected
  with the Louvain algorithm (using scanpy).

This notebook can be seen as a complement to the presented Case-Study on *Early-born Retinal Neurons*.

The data can be obtained from its corresponding GEO accession GSE122466.

The reference of the original study which published the data:

```
Giudice, Q. Lo, Leleu, M., Manno, G. La, & Fabre, P. J. (2019).
Single-cell transcriptional logic of cell-fate specification and axon guidance in early-born retinal neurons. 
Development (Cambridge), 146(17). https://doi.org/10.1242/dev.178103
```

In [1]:

```
import pickle
import warnings
warnings.filterwarnings('ignore')

import sklearn
import pandas as pd
import scanpy as sc
sc.settings.verbosity = 0
sc.logging.print_header()
import anndata as ad

import scboolseq
from scboolseq import scBoolSeq
```

```
scanpy==1.10.1 anndata==0.10.7 umap==0.5.6 numpy==1.26.4 scipy==1.13.0 pandas==2.2.2 scikit-learn==1.4.2 statsmodels==0.14.2 igraph==0.11.4 louvain==0.8.2 pynndescent==0.5.12
```

## Read-in the raw dataset¶

In [2]:

```
!test -f 'GSE122466_Merged5347cells_RAW.csv.gz' || wget -O 'GSE122466_Merged5347cells_RAW.csv.gz' 'https://www.ncbi.nlm.nih.gov/geo/download/?acc=GSE122466&format=file&file=GSE122466%5FMerged5347cells%5FRAW%2Ecsv%2Egz'
```

```
--2024-05-03 11:52:27--  https://www.ncbi.nlm.nih.gov/geo/download/?acc=GSE122466&format=file&file=GSE122466%5FMerged5347cells%5FRAW%2Ecsv%2Egz
Resolving www.ncbi.nlm.nih.gov (www.ncbi.nlm.nih.gov)... 2607:f220:41e:4290::110, 130.14.29.110
Connecting to www.ncbi.nlm.nih.gov (www.ncbi.nlm.nih.gov)|2607:f220:41e:4290::110|:443... connected.
HTTP request sent, awaiting response... 200 OK
Length: 10143933 (9.7M) [application/octet-stream]
Saving to: ‘GSE122466_Merged5347cells_RAW.csv.gz’

GSE122466_Merged534 100%[===================>]   9.67M   218KB/s    in 29s     

2024-05-03 11:53:03 (342 KB/s) - ‘GSE122466_Merged5347cells_RAW.csv.gz’ saved [10143933/10143933]
```

In [3]:

```
%%time
in_data = pd.read_csv("GSE122466_Merged5347cells_RAW.csv.gz", index_col=0, sep=";")
if not (in_data.columns.is_unique and in_data.index.is_unique):
    print("Check for duplicated index/columns")
in_data.head()
```

```
CPU times: user 5.88 s, sys: 357 ms, total: 6.24 s
Wall time: 6.24 s
```

Out[3]:

|  | Lane1\_AAACCTGAGATGTCGG | Lane1\_AAACCTGCAATCCAAC | Lane1\_AAACCTGGTTCCTCCA | Lane1\_AAACCTGTCCAATGGT | Lane1\_AAACGGGAGGCAATTA | Lane1\_AAACGGGCACCAGGCT | Lane1\_AAACGGGCACGGCGTT | Lane1\_AAACGGGCACTCAGGC | Lane1\_AAACGGGCATCCAACA | Lane1\_AAACGGGGTCCAAGTT | ... | Lane2\_TTTGGTTCATAAAGGT | Lane2\_TTTGGTTGTGGCCCTA | Lane2\_TTTGGTTTCTAACTCT | Lane2\_TTTGTCAAGCGTGAAC | Lane2\_TTTGTCAAGGGCTCTC | Lane2\_TTTGTCAAGTACCGGA | Lane2\_TTTGTCACATCGATGT | Lane2\_TTTGTCAGTAGAGGAA | Lane2\_TTTGTCAGTCATCCCT | Lane2\_TTTGTCATCAGTTCGA |
| --- | --- | --- | --- | --- | --- | --- | --- | --- | --- | --- | --- | --- | --- | --- | --- | --- | --- | --- | --- | --- | --- |
| Xkr4 | 0 | 0 | 0 | 0 | 0 | 0 | 0 | 0 | 0 | 0 | ... | 0 | 0 | 0 | 0 | 0 | 0 | 0 | 0 | 0 | 0 |
| Mrpl15 | 3 | 0 | 0 | 0 | 1 | 0 | 0 | 0 | 0 | 0 | ... | 0 | 1 | 1 | 0 | 1 | 0 | 0 | 2 | 1 | 0 |
| Lypla1 | 0 | 0 | 2 | 0 | 0 | 0 | 0 | 0 | 0 | 0 | ... | 1 | 0 | 0 | 0 | 0 | 0 | 0 | 0 | 1 | 0 |
| Gm37988 | 0 | 0 | 0 | 0 | 0 | 0 | 0 | 0 | 0 | 0 | ... | 0 | 0 | 0 | 0 | 0 | 0 | 0 | 0 | 0 | 0 |
| Tcea1 | 2 | 0 | 5 | 1 | 2 | 4 | 1 | 0 | 0 | 0 | ... | 1 | 3 | 0 | 1 | 1 | 0 | 1 | 1 | 1 | 0 |

5 rows × 5347 columns

In [4]:

```
batch_info = pd.DataFrame(
    data={
        "batch": in_data.columns.map(lambda x: x.split("_")[0]),
        "tag": in_data.columns.map(lambda x: x.split("_")[-1])
    }, 
    index=in_data.columns
)
batch_info.batch.value_counts()
```

Out[4]:

```
batch
Lane2    2674
Lane1    2673
Name: count, dtype: int64
```

## Standard Single-Cell RNA-seq Quality Control and Preprocessig Pipeline using `scanpy`¶

`scanpy` provides numerous functions for scRNA-seq data analysis.
Here we perform a standard pipeline which consists of:

0. Preprocessing:
   1. Analysing the percentage of mitochondrial genes.
   2. Doublet detection.
   3. Normalisation and log-transformation.
   4. Selecting Highly Variable Genes.
1. Performing a Principal Component Analysis (PCA) on top of previously selected Highly Variable Genes.
2. Computing a nearest neighbour distance matrix plus a neighbourhood graph of observations.
3. Performing a UMAP embedding.
4. Unsupervised community detection using the Louvain algorithm.

And finally:

6. The binarisation of scRNA-seq data with `scBoolSeq`

In [5]:

```
adata = ad.AnnData(in_data.T, obs=batch_info)
adata
```

Out[5]:

```
AnnData object with n_obs × n_vars = 5347 × 15176
    obs: 'batch', 'tag'
```

### Basic QC: Counts and Percentage of Mitochondrial Genes¶

In [6]:

```
%%time
# mitochondrial genes
adata.var["mt"] = adata.var_names.str.startswith(("Mt-", "mt-"))
sc.pp.calculate_qc_metrics(adata, qc_vars=["mt"], inplace=True, log1p=True)
adata
```

```
CPU times: user 843 ms, sys: 211 ms, total: 1.05 s
Wall time: 1.05 s
```

Out[6]:

```
AnnData object with n_obs × n_vars = 5347 × 15176
    obs: 'batch', 'tag', 'n_genes_by_counts', 'log1p_n_genes_by_counts', 'total_counts', 'log1p_total_counts', 'pct_counts_in_top_50_genes', 'pct_counts_in_top_100_genes', 'pct_counts_in_top_200_genes', 'pct_counts_in_top_500_genes', 'total_counts_mt', 'log1p_total_counts_mt', 'pct_counts_mt'
    var: 'mt', 'n_cells_by_counts', 'mean_counts', 'log1p_mean_counts', 'pct_dropout_by_counts', 'total_counts', 'log1p_total_counts'
```

In [7]:

```
sc.pl.violin(
    adata,
    ["n_genes_by_counts", "total_counts", "pct_counts_mt"],
    jitter=0.7,
    multi_panel=True,
)
sc.pl.scatter(adata, "total_counts", "n_genes_by_counts", color="pct_counts_mt")
```

In [8]:

```
sc.pp.filter_cells(adata, min_genes=100)
sc.pp.filter_genes(adata, min_cells=3)
```

### Doublet detection¶

In [9]:

```
%%time
adata.layers["counts"] = adata.X.copy()
sc.pp.scrublet(adata, batch_key="batch")
```

```
CPU times: user 1min 34s, sys: 12.1 s, total: 1min 46s
Wall time: 38.8 s
```

### Normalisation¶

In [10]:

```
sc.pp.normalize_total(
    adata, 
    target_sum=1e4, 
    exclude_highly_expressed=True,
    inplace=True
)
sc.pp.log1p(adata)
```

In [11]:

```
sc.pp.highly_variable_genes(
        adata, n_top_genes=2000, 
        batch_key="batch",
        flavor="cell_ranger",
)
```

In [12]:

```
sc.pl.highly_variable_genes(adata)
```

In [13]:

```
sc.tl.pca(adata)
sc.pl.pca_variance_ratio(adata, n_pcs=50, log=True)
```

In [14]:

```
sc.pl.pca(
adata,
color=["batch", "batch", "pct_counts_mt", "pct_counts_mt"],
dimensions=[(0, 1), (2, 3), (0, 1), (2, 3)],
ncols=2,
)
```

In [15]:

```
%%time
sc.pp.neighbors(adata)
sc.tl.umap(adata)
sc.tl.louvain(adata)
```

```
CPU times: user 12.3 s, sys: 27.2 ms, total: 12.4 s
Wall time: 9.9 s
```

In [16]:

```
sc.pl.umap(adata, color=["batch", "louvain", "pct_counts_mt", "doublet_score"], ncols=2)
```

### Filter out low-quality cells¶

In [17]:

```
from scboolseq import binarization as scbin
exclusion_criteria = ['doublet_score', 'pct_counts_mt']
with sklearn.config_context(transform_output="pandas"):
    # The QuantileBinarizer with parameter "alpha=1.5" is the standard "Tukey's Fences for Outlier Detection" 
    exclusion = scbin.QuantileBinarizer(alpha=1.5).fit(adata.obs[exclusion_criteria])
    excluded = exclusion.binarize(adata.obs[exclusion_criteria]).fillna(0)

(
    excluded.sum().to_frame().T
        .join(pd.Series(excluded.all(axis=1).sum(), index=[0], name="both"))
        .join(pd.Series(excluded.any(axis=1).sum(), index=[0], name="any"))
)
```

Out[17]:

|  | doublet\_score | pct\_counts\_mt | both | any |
| --- | --- | --- | --- | --- |
| 0 | 361.0 | 331.0 | 62 | 630 |

In [18]:

```
clean_adata =  adata[~excluded.any(axis=1), :].copy()
clean_adata
```

Out[18]:

```
AnnData object with n_obs × n_vars = 4717 × 15176
    obs: 'batch', 'tag', 'n_genes_by_counts', 'log1p_n_genes_by_counts', 'total_counts', 'log1p_total_counts', 'pct_counts_in_top_50_genes', 'pct_counts_in_top_100_genes', 'pct_counts_in_top_200_genes', 'pct_counts_in_top_500_genes', 'total_counts_mt', 'log1p_total_counts_mt', 'pct_counts_mt', 'n_genes', 'doublet_score', 'predicted_doublet', 'louvain'
    var: 'mt', 'n_cells_by_counts', 'mean_counts', 'log1p_mean_counts', 'pct_dropout_by_counts', 'total_counts', 'log1p_total_counts', 'n_cells', 'highly_variable', 'means', 'dispersions', 'dispersions_norm', 'highly_variable_nbatches', 'highly_variable_intersection'
    uns: 'scrublet', 'log1p', 'hvg', 'pca', 'batch_colors', 'neighbors', 'umap', 'louvain', 'louvain_colors'
    obsm: 'X_pca', 'X_umap'
    varm: 'PCs'
    layers: 'counts'
    obsp: 'distances', 'connectivities'
```

In [19]:

```
sc.pl.violin(
    clean_adata,
    ["n_genes_by_counts", "total_counts", "pct_counts_mt"],
    jitter=0.7,
    multi_panel=True,
)
sc.pl.scatter(clean_adata, "total_counts", "n_genes_by_counts", color="pct_counts_mt")
```

In [20]:

```
sc.pp.filter_cells(clean_adata, min_genes=100)
sc.pp.filter_genes(clean_adata, min_cells=3)
```

In [21]:

```
sc.pl.umap(
    clean_adata, 
    color=[
        "batch", "louvain", 
        "total_counts", "pct_counts_mt",
        "n_genes_by_counts", "doublet_score"
    ], 
    ncols=2
)
```

We are happy with these results because the previous normalisation and quality control were effective, we can judge this because:

1. The two batches (Lanes) have similar forms and are superimposed on one another.
2. Common technical biases do not fully account for the observed topologies on the projected space:
   - There is no cluster determined by the `doublet_score` or by the percentage of mitochondrial counts.
   - Some clusters have a higher sequencing depth but this does not represent a major axis of variation.
3. The projection coincides with that of the original article (see section Inferring Boolean signatures

## Binarisation of Highly Variable Genes with scBoolSeq¶

As explained in the main text, `scBoolSeq` should not be applied on the whole dataset but rather on the subset of "Highly Variable Genes"
which are deemed to be explicative of the phenomenon of interest.

These can be accessed using the "highly variable" annotation of the `var` attribute of an `AnnData` object as follows:

In [22]:

```
_hvgs = adata.var.index[adata.var['highly_variable']]
ref =  adata.to_df()[_hvgs]
ref.shape
```

Out[22]:

```
(5347, 2000)
```

### Create a scBoolSeq instance and fit it.¶

By overriding the parameter `zeroes_are=0` of `scBoolSeq`, cells with zero counts for genes classified as "ZeroInflated"
will have their coarse-grained activity assigned to be **inactive** rather than **unknown**.
This is a modelling choice that can be changed depending on the application.

In [23]:

```
%time scbool = scBoolSeq(zeroes_are=0)
```

```
CPU times: user 19 µs, sys: 0 ns, total: 19 µs
Wall time: 23.1 µs
```

In [24]:

```
%%time
with sklearn.config_context(transform_output="pandas"):
    scbool.fit(ref)
```

```
CPU times: user 6min 56s, sys: 8min 12s, total: 15min 9s
Wall time: 1min 5s
```

As shown by the ipython magic command `%% time`, estimating all parameters needed by `scBoolSeq` to coarse-grain experimental data
and generate synthetic counts from Boolean dynamics is relatively fast.

Treating a reference dataset comprised of 4717 cells and 1980 genes took a little over a minute `Wall time: 1min 15s` on modern
laptop with an `Intel i7-10875H (16) @ 5.100GHz` processor.

**NOTE**: The context manager for scikit-learn `with sklearn.config_context(transform_output="pandas")` is needed when using `scBoolSeq` and `scanpy` in the same interactive session.
The reason for this is that both `scanpy` and `scBoolSeq` use `scikit-learn` estimators under the hood, but in different maners:

- `scanpy` uses `AnnData` as its main datastructure. `AnnData` objects can have different backends.
  `scanpy` functions expect scikit-learn's config to be `transform_output="default"`.
- `scBoolSeq` uses `pandas.DataFrame` as its main datastructure. This enables semantic consistency,
  ensuring that the column names (gene names) are the same at training and inference time.
  This requires the opposite configuration `transform_output="pandas"`.

See more about the `transform_output` API in scikit-learn's docs.

It is important to note that `scBoolSeq` objects are pickable.
Even though fitting the instance (computing the classification parameters) is relatively fast,
there is no reason to compute them more than once:

- Save a scBoolSeq instance:

```
with open("scbool_GSE122466.pkl", "wb") as _out_f:
    pickle.dump(scbool, _out_f)
```

- Reload a scBoolSeq instance for use in another session:

```
with open("scbool_GSE122466.pkl", "rb") as _in_f:
    scbool = pickle.load(scbool, _in_f)
```

- Furthermore, `scBoolSeq` instances require little disk space:

```
$ du -sh scbool_GSE122466.pkl
# 808K scbool_GSE122466.pkl
```

In [25]:

```
scbool.criteria_.Category.value_counts()
```

Out[25]:

```
Category
ZeroInf      1362
Discarded     403
Bimodal       235
Name: count, dtype: int64
```

Some genes selected as being Highly Variable by `scanpy` have been rejected for binarisation by our classification scheme.

### Coarse-grain/Binarise expression data¶

In [26]:

```
%%time

with sklearn.config_context(transform_output="pandas"):
    raw_bin_ref = scbool.binarize(ref)

raw_bin_ref.head()
```

```
CPU times: user 528 ms, sys: 24.1 ms, total: 552 ms
Wall time: 552 ms
```

Out[26]:

|  | St18 | 3110035E14Rik | Mybl1 | Mcmdc2 | Tram1 | Terf1 | Tfap2d | Tfap2b | Mcm3 | Col9a1 | ... | Hba-a2 | Gm12146 | Gm16075 | Abi3 | Slc4a1 | Tgfbi | F2rl1 | Adssl1 | Papss2 | Ffar4 |
| --- | --- | --- | --- | --- | --- | --- | --- | --- | --- | --- | --- | --- | --- | --- | --- | --- | --- | --- | --- | --- | --- |
| Lane1\_AAACCTGAGATGTCGG | 0 | 0 | 0 | 0 | 1 | 0 | 0 | 0 | 0.0 | 1.0 | ... | NaN | NaN | NaN | NaN | NaN | NaN | NaN | NaN | NaN | NaN |
| Lane1\_AAACCTGCAATCCAAC | 0 | 0 | 0 | 0 | 0 | 0 | 0 | 0 | 1.0 | 0.0 | ... | NaN | NaN | NaN | NaN | NaN | NaN | NaN | NaN | NaN | NaN |
| Lane1\_AAACCTGGTTCCTCCA | 0 | 1 | 0 | 0 | 0 | 1 | 0 | 0 | 0.0 | 0.0 | ... | NaN | NaN | NaN | NaN | NaN | NaN | NaN | NaN | NaN | NaN |
| Lane1\_AAACCTGTCCAATGGT | 1 | 0 | 0 | 0 | 0 | 0 | 0 | 0 | 0.0 | 0.0 | ... | NaN | NaN | NaN | NaN | NaN | NaN | NaN | NaN | NaN | NaN |
| Lane1\_AAACGGGAGGCAATTA | 0 | 0 | 0 | 0 | 0 | 0 | 0 | 0 | 1.0 | 1.0 | ... | NaN | NaN | NaN | NaN | NaN | NaN | NaN | NaN | NaN | NaN |

5 rows × 2000 columns

As seen here, coarse-graining expression data is almost instant.

One might notice that numerous genes have `NaN` everywhere. This is expected as 387 genes were classified as being Discarded.

In [27]:

```
bin_ref = raw_bin_ref[raw_bin_ref.columns[raw_bin_ref.var() > 0]]
print(raw_bin_ref.shape, bin_ref.shape)
bin_ref.head(10)[bin_ref.var().sort_values(ascending=False).index[:10]].style.background_gradient()
```

```
(5347, 2000) (5347, 1597)
```

Out[27]:

|  | Serpinh1 | Lman1 | Msmo1 | Dapl1 | Mab21l2 | Mgarp | Slc2a1 | Glo1 | Mllt11 | Hmgb2 |
| --- | --- | --- | --- | --- | --- | --- | --- | --- | --- | --- |
| Lane1\_AAACCTGAGATGTCGG | 1.000000 | 0.000000 | 1.000000 | 1.000000 | 0.000000 | 0.000000 | 1.000000 | 1.000000 | 0.000000 | 1.000000 |
| Lane1\_AAACCTGCAATCCAAC | 0.000000 | 1.000000 | 0.000000 | 1.000000 | 0.000000 | 0.000000 | 1.000000 | 1.000000 | 0.000000 | 1.000000 |
| Lane1\_AAACCTGGTTCCTCCA | 0.000000 | 0.000000 | 1.000000 | 0.000000 | 1.000000 | 0.000000 | 1.000000 | 1.000000 | 1.000000 | 0.000000 |
| Lane1\_AAACCTGTCCAATGGT | 0.000000 | 0.000000 | 1.000000 | 0.000000 | 1.000000 | 1.000000 | 1.000000 | 1.000000 | 1.000000 | nan |
| Lane1\_AAACGGGAGGCAATTA | 1.000000 | 1.000000 | 1.000000 | 1.000000 | 1.000000 | 1.000000 | 1.000000 | 1.000000 | 0.000000 | 1.000000 |
| Lane1\_AAACGGGCACCAGGCT | 1.000000 | 0.000000 | 1.000000 | 0.000000 | 1.000000 | 0.000000 | 1.000000 | 0.000000 | 1.000000 | 0.000000 |
| Lane1\_AAACGGGCACGGCGTT | 1.000000 | 1.000000 | 0.000000 | 1.000000 | 0.000000 | 0.000000 | 1.000000 | 0.000000 | 0.000000 | 1.000000 |
| Lane1\_AAACGGGCACTCAGGC | 0.000000 | 0.000000 | 1.000000 | 1.000000 | 1.000000 | 0.000000 | 0.000000 | 0.000000 | 0.000000 | 1.000000 |
| Lane1\_AAACGGGCATCCAACA | 1.000000 | 0.000000 | 0.000000 | 1.000000 | 0.000000 | 1.000000 | 1.000000 | 0.000000 | 0.000000 | 1.000000 |
| Lane1\_AAACGGGGTCCAAGTT | 0.000000 | 1.000000 | 1.000000 | 0.000000 | 0.000000 | 1.000000 | 0.000000 | 0.000000 | 1.000000 | 1.000000 |

## Inferring the Boolean signature of Louvain clusters¶

One may want to pool groups of cells together to perform analyses on some sort of consensus *Boolean signature*, rather than a single cells.
For this purpose, there is a module `scboolseq.meta` containing classes and functions facilitating the agreggation of Boolean cells.
By defining these *meta-observations* of Boolean values, one can reason in terms of the common/consensus activation state of cell clusters.

`scboolseq.meta.CellAggregator` has two main arguments:

- `binary_df`, the first argument with coarse-grained cells (the output of
  `scboolseq.scBoolSeq().fit_transform(log_expression)`).
- `groups`, the second argument, which should be either a `pandas GroupBy` object or a dictionnary whose keys are the group names
  and values are `pandas.Index`.

Combining these two, the aggregator has numerous properties representing different aggregation strategies:

In [28]:

```
%%time
aggregator = scboolseq.meta.CellAggregator(
    bin_ref, 
    clean_adata.obs.groupby("louvain", observed=True)
)
aggregator
```

```
CPU times: user 10.7 ms, sys: 2.8 ms, total: 13.5 ms
Wall time: 12.4 ms
```

Out[28]:

```
<scboolseq.meta.aggregations.CellAggregator at 0x72e8b2cde910>
```

Here, we demonstrate what we call *binary strategies*.
These use the following statistics to yield a consensus signature:

In [29]:

```
scboolseq.meta.CellAggregator._binary_strategies
```

Out[29]:

```
('mode', 'median', 'min', 'max')
```

We can asssess if these strategies permit identifying the groups (in this case cell clusters determined via the Louvain algorithm:
`scanpy.tl.louvain(adata)`

In [30]:

```
%%time
scores = {
    k: scboolseq.meta.meta_marker_counter(getattr(aggregator, k))
    for k in aggregator._binary_strategies
}
```

```
CPU times: user 4.39 s, sys: 4.65 ms, total: 4.4 s
Wall time: 4.39 s
```

In [31]:

```
{k: df.mean().astype(int).to_dict() for k, df in scores.items()}
```

Out[31]:

```
{'mode': {'n_positive_markers': 7, 'n_negative_markers': 0},
 'median': {'n_positive_markers': 7, 'n_negative_markers': 0},
 'min': {'n_positive_markers': 2, 'n_negative_markers': 0},
 'max': {'n_positive_markers': 0, 'n_negative_markers': 90}}
```

The **mode** strategy corresponds to taking the by-group most frequent coarse-grained value, for each gene.
It seems like this strategy allows yields a high average number of positive markers (genes being classified as active
within the group and **in no other group**).

In [32]:

```
_selected_mode = "mode"
metabin = scores[_selected_mode]
metabin
```

Out[32]:

|  | n\_positive\_markers | n\_negative\_markers |
| --- | --- | --- |
| 0 | 3 | 0 |
| 1 | 7 | 0 |
| 10 | 1 | 0 |
| 11 | 2 | 1 |
| 2 | 3 | 0 |
| 3 | 2 | 0 |
| 4 | 17 | 1 |
| 5 | 19 | 0 |
| 6 | 23 | 0 |
| 7 | 12 | 0 |
| 8 | 2 | 0 |
| 9 | 0 | 0 |

To better assess the quality of these markers, we can add them to the `adata.obs` DataFrame.
We can subsequently visualise the number of markers with the corresponding clusters on the UMAP projection.

In [33]:

```
for louv in clean_adata.obs.louvain.unique():
    _nm = metabin.loc[louv, :].to_frame().T
    for c in _nm.columns:
        clean_adata.obs.loc[clean_adata.obs.louvain == louv, f"{c}_{_selected_mode}"] = _nm.loc[louv, c]
```

In [34]:

```
sc.pl.umap(clean_adata, color=["louvain", f"n_positive_markers_{_selected_mode}"], ncols=2)
```

We observe that some clusters have few Boolean markers.
Extremes representing the starting point of the differentiation process (Early Retinogenesis)
and the final phenotypes have a higher number of markers, while transient clusters have less extreme expression values
and will therefore have less genes whose coarse-grained activity enables their identification.

These can be compared with the figure published in the original article:

```
Giudice, Q. Lo, Leleu, M., Manno, G. La, & Fabre, P. J. (2019).
Single-cell transcriptional logic of cell-fate specification and axon guidance in early-born retinal neurons. 
Development (Cambridge), 146(17). https://doi.org/10.1242/dev.178103
```

In [ ]:

```

```
