# Supplementary material for: scBoolSeq: Linking scRNA-seq statistics and Boolean dynamics
Source: PLoS Comput Biol. 2024 Jul 8;20(7):e1011620. doi: 10.1371/journal.pcbi.1011620 (PMC11257695; doi:10.1371/journal.pcbi.1011620)
Supplement: S1 Notebooks — The notebooks are provided as static HTML files, and Boolean networks as textual files in BoolNet format. See the Data availability statement for links to executable notebooks and code. (ZIP) [file pcbi.1011620.s001.zip › synthetic scRNA-Seq from star network.html]

synthetic scRNA-Seq from star network


# Synthetic expression data from asynchronous random walks on star network¶

In this series of notebooks, we demonstrate how scBoolSeq can be employed to generate synthetic scRNA-Seq datasets from Boolean states of trajectories of mechanistic Boolean models.

This notebook focuses on a toy model where a transcription factor progressively activates its target genes.

In [1]:

```
from IPython.display import display, HTML
display(HTML("<style>.container { width:100% !important; }</style>"))
import warnings
warnings.filterwarnings("ignore")
```

In [2]:

```
import pandas as pd
import numpy as np
import random
from colomoto.minibn import * # for Boolean network manipulation
from scboolseq import scBoolSeq

# set seed for reproducible results
_rng_seed = 19834650
# use a Generator instead of numpy's singleton
_rng = np.random.default_rng(_rng_seed)
random.seed(_rng_seed)
```

## Load Boolean network model¶

In [3]:

```
bn = BooleanNetwork.load("models/star.bnet")
bn
```

Out[3]:

```
gene1 <- tf
gene10 <- tf
gene2 <- tf
gene3 <- tf
gene4 <- tf
gene5 <- tf
gene6 <- tf
gene7 <- tf
gene8 <- tf
gene9 <- tf
tf <- 1
```

In [4]:

```
bn.influence_graph()
```

```
# computing graph layout...
```

Out[4]:

## Simulation with random walk¶

With the asynchronous update mode, the activation of the genes can be made in any order. Here, we randomly sample one trajectory of this model, which essentially boils down to selecting a random ordering of genes that get activated.

Let us first specify the initial state of the network:

In [5]:

```
initial_state = bn.zero()
initial_state["tf"] = 1
initial_state
```

Out[5]:

```
{'tf': 1,
 'gene1': 0,
 'gene2': 0,
 'gene3': 0,
 'gene4': 0,
 'gene5': 0,
 'gene6': 0,
 'gene7': 0,
 'gene8': 0,
 'gene9': 0,
 'gene10': 0}
```

Then, we use `minibn` to generate a random walk in the asynchronous dynamics of the Boolean network from the given initial state:

In [6]:

```
dynamics = FullyAsynchronousDynamics(bn)
random_walk_df = pd.DataFrame(dynamics.random_walk(initial_state, steps=10))
random_walk_df
```

Out[6]:

|  | tf | gene1 | gene2 | gene3 | gene4 | gene5 | gene6 | gene7 | gene8 | gene9 | gene10 |
| --- | --- | --- | --- | --- | --- | --- | --- | --- | --- | --- | --- |
| 0 | 1 | 0 | 0 | 0 | 0 | 0 | 0 | 0 | 0 | 0 | 0 |
| 1 | 1 | 0 | 0 | 0 | 0 | 0 | 0 | 0 | 1 | 0 | 0 |
| 2 | 1 | 1 | 0 | 0 | 0 | 0 | 0 | 0 | 1 | 0 | 0 |
| 3 | 1 | 1 | 0 | 1 | 0 | 0 | 0 | 0 | 1 | 0 | 0 |
| 4 | 1 | 1 | 0 | 1 | 0 | 0 | 1 | 0 | 1 | 0 | 0 |
| 5 | 1 | 1 | 0 | 1 | 0 | 0 | 1 | 0 | 1 | 1 | 0 |
| 6 | 1 | 1 | 0 | 1 | 0 | 0 | 1 | 0 | 1 | 1 | 1 |
| 7 | 1 | 1 | 0 | 1 | 1 | 0 | 1 | 0 | 1 | 1 | 1 |
| 8 | 1 | 1 | 1 | 1 | 1 | 0 | 1 | 0 | 1 | 1 | 1 |
| 9 | 1 | 1 | 1 | 1 | 1 | 0 | 1 | 1 | 1 | 1 | 1 |
| 10 | 1 | 1 | 1 | 1 | 1 | 1 | 1 | 1 | 1 | 1 | 1 |

## Retrieve reference dataset¶

In [7]:

```
%ls data_filtered_vargenes/*csv
```

```
data_filtered_vargenes/GSE122466_Retina.csv
data_filtered_vargenes/GSE122466_Retina_vargenes_batch1.csv
data_filtered_vargenes/GSE130973_human_skin.csv
data_filtered_vargenes/GSE156234_Macrophages.csv
data_filtered_vargenes/GSE60361_mouse_brain.csv
data_filtered_vargenes/GSE81682_Hematopoiesis.csv
```

In [8]:

```
ref_data = pd.read_csv("data_filtered_vargenes/GSE81682_Hematopoiesis.csv", index_col=0)
ref_data.head()
```

Out[8]:

|  | 8430408G22Rik | Plp1 | Zfp947 | Bhlhb9 | Vps35 | Slc18a1 | Fam107b | Gm14230 | Plekhn1 | Ankrd6 | 9030619P08Rik | Prtn3 | Lrrn4 | Mrgpre | Gm25926 | Plppr3 | Memo1 | Cdkn1c | Gm2a | Phxr4 | B2m | Tbxas1 | Glipr2 | Tcf15 | Trim58 | D930028M14Rik | 1700010I14Rik | Gm10384 | Sla2 | Trp53i13 | Gm17586 | Gypc | Clic5 | Tubb4b | AC149090.1 | Gm26580 | Clec1a | Ccl9 | Gimap3 | Zscan18 | Spi1 | Ccl6 | 2900041M22Rik | Klk8 | Gm37637 | Gp9 | Idh3a | Akr1c13 | 2810408A11Rik | Npr2 | Ephx1 | Pik3ip1 | ENSMUSG00000043858 | Gm26789 | Grina | Txnip | Gm26541 | Dnajb3 | Msn | Gm37124 | Srd5a1 | Bhlhe40 | Pomgnt2 | Fcer1g | Gm37298 | Lmo1 | Rab20 | Cd69 | Gm2788 | Gm14276 | Napa | Cd24a | Nsun2 | Marveld2 | Ms4a4b | Gm17590 | Cdk6 | Tada2b | Gm5113 | Mki67 | Btg2 | Gnat2 | Tcaf1 | Slc35e4 | Reep2 | 8430419K02Rik | Pdgfd | H2-K1 | Sesn1 | Bcap29 | Gm21814 | Eif5a | Stk25 | Maged1 | Zfp449 | 9130604C24Rik | 6430590A07Rik | Tcirg1 | Il4 | Slc35a4 | Cysltr2 | Ap3m2 | Dsel | Cmtm7 | Arl8b | Gbx2 | Smim10l2a | Cyth4 | S1pr4 | Gdi1 | Hdhd3 | Vill | Slc38a6 | Calu | Gm16565 | Smim3 | Sar1a | 4833407H14Rik | B230217O12Rik | Ccdc39 | Spata2l | Arxes2 | Dut | Ifngr1 | Rasa3 | Fbxl2 | Pde4b | Ptp4a3 | Cdt1 | Fxyd1 | Arl11 | Shisa8 | Dleu7 | Tmem173 | Atxn10 | Rpn2 | Ccl3 | Nudt16 | Kif3c | Hars | Ccl4 | Myo1g | Cpq | Cox6a2 | Sdc3 | Dok2 | Fcgrt | Elmo1 | Cyc1 | Calr | Gpr18 | Tbxa2r | Gpr183 | Naaa | Gm11110 | Alad | Taz | Echdc3 | Cd300a | Slc39a4 | Zfp128 | Gm26819 | Ispd | Itm2b | Zfp647 | Gm16861 | Nek3 | Gata2 | Prss16 | Lppos | Lhcgr | Fundc1 | Lat | Tnfrsf26 | Prr36 | Vldlr | Mfge8 | Enkur | Rcor2 | Ndn | Btla | Zyx | Cd33 | Dkc1 | Rab37 | Cdcp1 | Hoxa9 | Oat | Vegfc | Gas6 | Oasl2 | Tacc1 | Colgalt1 | Xdh | Rab32 | Dusp2 | Tmem176a | Gnai3 | Rnf13 | Elane | Cd48 | Mthfd1 | Igkc | Fnta | Bok | Ripply3 | Ptger2 | P2rx7 | Wdfy2 | Eif1a | Gstm7 | Tmem14c | 2900018N21Rik | Mpl | Sash3 | Tgm2 | Gm19569 | Ehd3 | Cish | Calml4 | Mesdc2 | Nfil3 | Tcn2 | Saraf | Meis1 | Serpina3g | Pcx | Gpr171 | Cks1b | Coa5 | Arhgap27os3 | Farsa | Ctsl | Iigp1 | P2ry14 | Cd82 | Slc18a2 | Cd302 | Parp12 | Isyna1 | S100a8 | B130034C11Rik | Arhgap27 | Klf1 | Gm26740 | Cd84 | Bag2 | Tmem123 | Emilin1 | 4930568A12Rik | Clec11a | Mat2a | Tespa1 | Wfdc17 | Tspan13 | Dyrk2 | S100a9 | F10 | Pde1b | Gcnt2 | Ppic | Zfp72 | Gm4841 | Ckb | C130013H08Rik | Spo11 | Ctss | Vwa5a | Serpinb1a | Trib3 | Slc28a2 | Xbp1 | Tsc22d3 | Cd74 | Nhlrc1 | Rac2 | Il15 | Ift43 | Tnfrsf13c | Gm10451 | Plod2 | Minpp1 | Cs | Gm21967 | Trim27 | Rrm1 | Krt18 | Rap1b | Anxa2 | D130040H23Rik | Tmsb4x | 1810034E14Rik | 2810021J22Rik | F630028O10Rik | Cyb5r3 | B3gnt3 | Laptm5 | Adam11 | Rab17 | Pkia | Hist3h2ba | Cyp27a1 | Zfp36 | Mcm5 | Ssc4d | Gm45837 | Lmcd1 | Fgf3 | Car1 | Adssl1 | Il21r | Slc50a1 | Serpinb9 | Pttg1ip | Fam83d | Tspan32 | Tnfrsf13b | Gdi2 | Nim1k | Mapk12 | Wfdc18 | Thbs1 | Chil5 | Mpst | Selenop | Gm43852 | Hk3 | Irgm1 | Nceh1 | Myl10 | Tmx1 | Zcchc24 | Acsl5 | Tnfaip8 | 4632427E13Rik | Hspa9 | Hid1 | Gstt1 | Il12rb1 | Ficd | Bbs10 | Esam | Unc93b1 | Casp4 | F2r | Dynlt3 | Adgrg1 | Tmem38a | Stap1 | Cep170b | Lpxn | Frmd8 | Doc2g | Ighv1-23 | Hrh2 | Bgn | Ifi206 | Fes | Apol7e | Srl | Ifi47 | Cdca7 | Srm | Smagp | Zfp418 | Zfp882 | Mmp2 | Fam32a | Flna | Lyz2 | Cd53 | Mapk11 | Sptbn1 | Mapre3 | Nr0b2 | Ptprc | Gulp1 | Cd63 | Nsg1 | Fam131a | Dntt | Ifi213 | Tcp1 | Stard8 | Clptm1l | Sigirr | Robo3 | Il17rb | Mcfd2 | Vpreb1 | Casp12 | Zbtb3 | Ppt1 | H1f0 | C730034F03Rik | Tpst2 | Pnrc1 | Sdhaf2 | Hnrnpdl | Cyp7b1 | Ptpre | Slc25a45 | Wdr35 | Capg | Sf3b3 | Gata1 | Spry1 | Abce1 | Rnf180 | Golph3l | P2ry10 | Adgrg3 | Fkbp4 | F2rl2 | Gbp2 | Plcg2 | Ccdc189 | 4930486L24Rik | E230001N04Rik | Itih5 | Chrnb1 | Gm16386 | Thnsl2 | Gm16712 | Top2a | Gm5577 | Nt5c3 | Nlrp10 | Ctf1 | Il18bp | Il1rl2 | Gm5148 | Car2 | Vim | A630033H20Rik | Cd81 | Zfp563 | Ctla2a | Mfsd2b | Idh2 | Tert | Pwwp2b | Atp6ap2 | Ces2g | Gba2 | 4931428F04Rik | Apoe | Sdha | Dlg3 | Igkv4-50 | Dhx58 | Zfp661 | Ccr9 | Rgs2 | Ctsc | Parp8 | Tspo2 | 5430420F09Rik | Pear1 | Ltb | Gm26512 | AA467197 | Unc5cl | Mtpn | Mcm6 | Atp2a3 | Tyms | Il12a | Celf2 | Axl | Tpm4 | Gm3739 | Cyp2j9 | Fxyd5 | B230216N24Rik | Trbc2 | Fam161b | Gm30948 | Apbb1 | Igfbp4 | Pcp4l1 | Rassf5 | Cyp2r1 | Smoc1 | Srgn | Pygm | Gm43200 | Gca | Arhgef6 | Fah | BC017643 | Ak3 | Icam1 | Gm10505 | C1qbp | Efna1 | AW112010 | Tnks1bp1 | Anxa1 | Fgf11 | Layn | Gm19331 | Rbpms2 | Tspan3 | Ighv9-1 | Gm43201 | Arrdc1 | Hjurp | 4921507P07Rik | Sla | Ms4a6b | Zfp551 | 2810468N07Rik | Tnf | Serp1 | Mfap2 | Arhgef18 | Slc25a29 | Hmox1 | Pkm | Arhgap15 | Ddx39 | Fv1 | Gnb5 | Tes | Lysmd2 | Mmp11 | Pgrmc1 | Gm42979 | Efna4 | Uba7 | Xist | Sumo3 | Flt3 | Mycn | 1300017J02Rik | Scn1b | Rab38 | ENSMUSG00000096970 | Tnfaip2 | Hdac2 | Ccr7 | Irf6 | Mndal | Fut4 | Casp1 | Slc14a1 | Rit1 | Ifi203 | Zfp93 | Calr3 | 2210010C04Rik | Gm38243 | Gm43313 | Slc25a5 | Bcam | Ighv1-74 | Traf3ip3 | Lgals9 | Gm26982 | Clec10a | Klhl8 | Islr | Ighv1-76 | Crlf3 | Ighv1-77 | Lcp2 | Rapsn | Cd38 | Gm37423 | Selplg | Clip3 | Ccdc157 | Uggt2 | Mpo | Eya1 | Ctso | Gm26917 | Rarb | Cmtm6 | Metrnl | Rgs1 | Ptpn6 | Cd1d1 | Lyrm1 | Prkaa2 | Gm4759 | Aplp2 | Gm8995 | Gng11 | Tmem150b | Abhd6 | Gm5111 | Ankrd50 | Neurl3 | Slc44a2 | Gm128 | Fam132a | 2810414N06Rik | M6pr | Ankrd33b | Hexa | Pdcd1lg2 | Dnajc9 | Lck | Ccnb2 | Wls | Gramd1a | Gm37642 | St8sia4 | Igkv12-44 | Sqstm1 | Slc25a35 | Tipin | 4930555A03Rik | Rn18s-rs5 | Samd12 | B9d1 | Ncf4 | Myc | Uaca | Irf9 | Paics | Gstm1 | Padi4 | Tor3a | Rgs18 | Slc26a6 | Ppm1g | Zfand5 | Smpd5 | Hdac9 | Gnl3 | Ecm1 | Rnase6 | Lgals3bp | Slc2a10 | Rdh14 | Icos | Dnah1 | Amt | Fut8 | Itpkc | Ap1s2 | Rgs11 | Hpcal1 | Gm43178 | Rnase4 | Adgrl4 | Nek6 | Clk1 | Eya2 | Csf3r | Fkbp1b | Pirb | Msmo1 | Gm15133 | Pim2 | Cldn15 | Gm28557 | Ric8a | Triqk | Spns2 | Cebpe | Tspan14 | Itm2a | Ddx1 | Sh3tc1 | Slc22a18 | Lcp1 | Casp2 | Epsti1 | Ifi44 | Phf11b | Ctsb | H2afy | Ctsg | Gimap9 | Hsd3b7 | Smpdl3a | Gm43162 | Trim47 | Cpa3 | Cat | Cers2 | Ifit3 | Ifit1bl1 | Slc9a3r2 | Mcm3 | Fscn1 | Thbd | Cd93 | Sept6 | Irf5 | Rab19 | Ctsz | Rhbdf1 | Wfs1 | Mef2c | Blvrb | Gimap1 | Gimap7 | Gimap4 | 4930515G01Rik | Hgfac | Gimap6 | Gm15201 | Gimap5 | BC035044 | Ly6e | Hs3st1 | Zfp62 | Vpreb3 | Kcnh2 | Hn1l | Hlf | 9930012K11Rik | Sgsm1 | Gmpr | Lancl1 | Slc16a1 | Plcb2 | Sdpr | Rhoh | Gm38250 | Gm26532 | Dock2 | Pdlim2 | Clk4 | Nubp2 | Treml2 | Sell | Polm | Pglyrp2 | Gm37829 | Tsc22d1 | 9130008F23Rik | Gm37558 | Gm37706 | Ptprcap | Hck | Adamts10 | Ly6a | Gm37598 | Anxa5 | Parvg | Acer2 | Cd79a | Tmem175 | Fcrl1 | Dusp22 | Ms4a4c | Bank1 | Gm43149 | Gatsl3 | BC026585 | Wfdc2 | Camk1 | Dgkq | Treml1 | Ebi3 | Gm27252 | Magee1 | Sdc2 | Ldhb | Kmo | Bpifb5 | Trim30a | Ifitm1 | Ddx58 | Ifitm3 | Rmnd5b | Gm26772 | Gm16151 | Gm16150 | Tagln2 | 3110083C13Rik | Cyp39a1 | Tspyl3 | Dennd1c | Slc2a1 | Large1 | AB124611 | Gm17096 | Stxbp4 | Muc3a | Awat2 | Plac8 | AI506816 | Canx | Alox5 | Prdx6 | F11r | Alg2 | Lurap1 | Ttc30b | Inpp5k | Dcdc2b | Tnfsf4 | Atp6ap1 | Dapp1 | 1500002C15Rik | Tuba4a | Gm43254 | ENSMUSG00000029333 | Gclm | Ruvbl2 | Apobr | Nap1l3 | Cct6a | Vav1 | Rfc2 | Gm16001 | Atp1b1 | Gm19708 | Nradd | Ypel3 | Tor4a | Gm19590 | Ivns1abp | Gm15991 | Upp1 | Gm6157 | Eif5a2 | C1qtnf6 | 4933424M12Rik | Mageh1 | Gm11696 | Cbfb | Zfp422 | Mfng | Klf8 | Stk17b | Met | Ufsp1 | Tmem150a | AI606181 | Ikzf2 | Slc34a2 | 1700066B19Rik | Mcm7 | Fbxo48 | Ecscr | Lyplal1 | Ankrd37 | Plek | Mapkbp1 | Ppp1r3d | Exoc3l2 | Tmem53 | Wsb1 | Rtkn | Tfr2 | Leprot | Arhgap25 | Nupr1 | Plpp7 | G730003C15Rik | Rsrp1 | Samsn1 | Prdx3 | Gm37663 | Itgb3 | Rtn4r | Nrros | Dennd2d | Por | Tspear | Hsd17b12 | Capza2 | Osgin1 | Spns3 | Cd34 | 4921531C22Rik | Trmt2a | Gm26692 | Col16a1 | Cd46 | Ptgds | Uqcrc1 | Gm28512 | Hacd4 | Ttc39c | Igkv1-135 | Fam129c | Rhof | Sdcbp | Rcsd1 | Mmrn1 | Ccser1 | Ccdc92b | Tnfaip8l1 | Tprgl | Gem | D630039A03Rik | Lrrc36 | Map10 | Gm38071 | Ccng2 | Ldha | Ramp1 | Ucp2 | Sdsl | Ttc4 | Afap1l1 | Ccnd2 | Sult1a1 | Ube2l6 | Clec12a | Gm43643 | Smtnl1 | Aqp1 | Large2 | Creg1 | Fam221a | Muc13 | Slc25a11 | Grap | Ncf1 | B4galt4 | Gm16758 | Nucb1 | Fam69b | Tmem35b | Mx1 | Ppp1r26 | Mx2 | Ppp1r15a | Pdzk1ip1 | Coro1a | Mob3c | Oas2 | Kctd12b | Mefv | Gm12250 | Sh2d5 | Igtp | Ermap | Smu1 | Maged2 | Mcm4 | Gm42576 | Anxa6 | 1700123M08Rik | Rtp4 | Sh3bgrl | Napsa | Kctd21 | Chpf | Gm7160 | Neurl1b | Cct3 | Nkx2-3 | Wdr78 | Dusp1 | Def8 | Rbm5 | BC016579 | Emb | Elovl1 | Myo1f | Lax1 | Cyp2j6 | Pstpip1 | Emp1 | Cd44 | Mtch2 | Pgm2 | Krt7 | Mir155hg | 1110028F11Rik | Acot7 | Rnd2 | Irf1 | H2-DMa | Zfp263 | Rxra | Klhl6 | A130014A01Rik | Ssbp2 | Rbp1 | Rsl1d1 | Gjb3 | Asah1 | Tmem37 | Spcs3 | Cenpa | Gfpt2 | Tmprss3 | Pqlc3 | Dhrs3 | Jak3 | Fam213b | Pafah2 | Jun | Lamp1 | Camsap3 | Grtp1 | Gm16794 | Cacybp | Slfn8 | Stom | H2-DMb1 | H2-D1 | Oas1c | Tas1r1 | Rab44 | 4930579K19Rik | Fos | Nedd4 | Cyb561 | Mapkapk3 | Inpp4b | Ccdc69 | Slfn2 | Tmem220 | 2610035D17Rik | Snap23 | Nprl2 | Rasal3 | Ighv1-82 | Batf | Tmem121 | Rrm2 | Surf4 | Zfp386 | Mamdc4 | B230217C12Rik | Zbtb42 | Sstr2 | Pld2 | Cers5 | Ighd | Ighm | Cyp4v3 | Limd2 | Nlrp1a | H2-Q6 | P4hb | Grn | H2-Q7 | Gm10863 | Hhex | Pcbp4 | Tubb5 | Itgb7 | Gpam | Cbx5 | Fam184a | Tmem106a | Adamtsl5 | Cd79b | Nfe2 | H2-Ob | Tcp11l2 | Shisa5 | Samd10 | Hspa12b | Tcea2 | Ctsd | Rgs19 | Procr | Tnni2 | Acap1 | Bin2 | Ropn1l | Lmbrd2 | Laptm4b | Il7r | Capsl | Angpt1 | Spag1 | H2-Ab1 | Gfi1b | H2-Aa | Ctsw | Hspa5 | Tbc1d10c | Rom1 | Smpd2 | Ctsf | Fgd2 | Cct8 | Mamdc2 | Stip1 | Gm28043 | Cd27 | Rdh5 | Aldoc | Prss57 | Arpc1b | Fut7 | Cd86 | Serpinf1 | Cd9 | Gm37274 | Wdr38 | Angptl6 | Smad6 | Chad | Arhgdib | Eogt | Llgl2 | Cst7 | E130215H24Rik | Gm27201 | Pcna | Rnf114 | Gchfr | Cacfd1 | Jam3 | Fkbp1a | Eps8 | Epb41l1 | Traf1 | Pdia3 | Mgst1 | Slc2a6 | Spint1 | Lmo2 | Eng | Skap2 | Vamp5 | Plxna1 | Klrb1f | Tmem176b | Mcm2 | Slc52a3 | Cct7 | Ptgs1 | Cnn2 | Prim1 | Rwdd2a | Matk | Atp5b | Cdk4 | Robo4 | Gm15915 | Gm10101 | Abhd4 | Rab36 | Metap2 | Fzr1 | Pa2g4 | Smim24 | Gm16104 | Perp | Fig4 | Myct1 | Lsp1 | Alpk3 | Zfp28 | Zfp78 | Zfp773 | Mill2 | Gpr4 | Nectin2 | Tyrobp | Plekhf1 | Nkg7 | Osbpl1a | Slc27a6 | Gm4951 | Zfp438 | Rab18 |
| --- | --- | --- | --- | --- | --- | --- | --- | --- | --- | --- | --- | --- | --- | --- | --- | --- | --- | --- | --- | --- | --- | --- | --- | --- | --- | --- | --- | --- | --- | --- | --- | --- | --- | --- | --- | --- | --- | --- | --- | --- | --- | --- | --- | --- | --- | --- | --- | --- | --- | --- | --- | --- | --- | --- | --- | --- | --- | --- | --- | --- | --- | --- | --- | --- | --- | --- | --- | --- | --- | --- | --- | --- | --- | --- | --- | --- | --- | --- | --- | --- | --- | --- | --- | --- | --- | --- | --- | --- | --- | --- | --- | --- | --- | --- | --- | --- | --- | --- | --- | --- | --- | --- | --- | --- | --- | --- | --- | --- | --- | --- | --- | --- | --- | --- | --- | --- | --- | --- | --- | --- | --- | --- | --- | --- | --- | --- | --- | --- | --- | --- | --- | --- | --- | --- | --- | --- | --- | --- | --- | --- | --- | --- | --- | --- | --- | --- | --- | --- | --- | --- | --- | --- | --- | --- | --- | --- | --- | --- | --- | --- | --- | --- | --- | --- | --- | --- | --- | --- | --- | --- | --- | --- | --- | --- | --- | --- | --- | --- | --- | --- | --- | --- | --- | --- | --- | --- | --- | --- | --- | --- | --- | --- | --- | --- | --- | --- | --- | --- | --- | --- | --- | --- | --- | --- | --- | --- | --- | --- | --- | --- | --- | --- | --- | --- | --- | --- | --- | --- | --- | --- | --- | --- | --- | --- | --- | --- | --- | --- | --- | --- | --- | --- | --- | --- | --- | --- | --- | --- | --- | --- | --- | --- | --- | --- | --- | --- | --- | --- | --- | --- | --- | --- | --- | --- | --- | --- | --- | --- | --- | --- | --- | --- | --- | --- | --- | --- | --- | --- | --- | --- | --- | --- | --- | --- | --- | --- | --- | --- | --- | --- | --- | --- | --- | --- | --- | --- | --- | --- | --- | --- | --- | --- | --- | --- | --- | --- | --- | --- | --- | --- | --- | --- | --- | --- | --- | --- | --- | --- | --- | --- | --- | --- | --- | --- | --- | --- | --- | --- | --- | --- | --- | --- | --- | --- | --- | --- | --- | --- | --- | --- | --- | --- | --- | --- | --- | --- | --- | --- | --- | --- | --- | --- | --- | --- | --- | --- | --- | --- | --- | --- | --- | --- | --- | --- | --- | --- | --- | --- | --- | --- | --- | --- | --- | --- | --- | --- | --- | --- | --- | --- | --- | --- | --- | --- | --- | --- | --- | --- | --- | --- | --- | --- | --- | --- | --- | --- | --- | --- | --- | --- | --- | --- | --- | --- | --- | --- | --- | --- | --- | --- | --- | --- | --- | --- | --- | --- | --- | --- | --- | --- | --- | --- | --- | --- | --- | --- | --- | --- | --- | --- | --- | --- | --- | --- | --- | --- | --- | --- | --- | --- | --- | --- | --- | --- | --- | --- | --- | --- | --- | --- | --- | --- | --- | --- | --- | --- | --- | --- | --- | --- | --- | --- | --- | --- | --- | --- | --- | --- | --- | --- | --- | --- | --- | --- | --- | --- | --- | --- | --- | --- | --- | --- | --- | --- | --- | --- | --- | --- | --- | --- | --- | --- | --- | --- | --- | --- | --- | --- | --- | --- | --- | --- | --- | --- | --- | --- | --- | --- | --- | --- | --- | --- | --- | --- | --- | --- | --- | --- | --- | --- | --- | --- | --- | --- | --- | --- | --- | --- | --- | --- | --- | --- | --- | --- | --- | --- | --- | --- | --- | --- | --- | --- | --- | --- | --- | --- | --- | --- | --- | --- | --- | --- | --- | --- | --- | --- | --- | --- | --- | --- | --- | --- | --- | --- | --- | --- | --- | --- | --- | --- | --- | --- | --- | --- | --- | --- | --- | --- | --- | --- | --- | --- | --- | --- | --- | --- | --- | --- | --- | --- | --- | --- | --- | --- | --- | --- | --- | --- | --- | --- | --- | --- | --- | --- | --- | --- | --- | --- | --- | --- | --- | --- | --- | --- | --- | --- | --- | --- | --- | --- | --- | --- | --- | --- | --- | --- | --- | --- | --- | --- | --- | --- | --- | --- | --- | --- | --- | --- | --- | --- | --- | --- | --- | --- | --- | --- | --- | --- | --- | --- | --- | --- | --- | --- | --- | --- | --- | --- | --- | --- | --- | --- | --- | --- | --- | --- | --- | --- | --- | --- | --- | --- | --- | --- | --- | --- | --- | --- | --- | --- | --- | --- | --- | --- | --- | --- | --- | --- | --- | --- | --- | --- | --- | --- | --- | --- | --- | --- | --- | --- | --- | --- | --- | --- | --- | --- | --- | --- | --- | --- | --- | --- | --- | --- | --- | --- | --- | --- | --- | --- | --- | --- | --- | --- | --- | --- | --- | --- | --- | --- | --- | --- | --- | --- | --- | --- | --- | --- | --- | --- | --- | --- | --- | --- | --- | --- | --- | --- | --- | --- | --- | --- | --- | --- | --- | --- | --- | --- | --- | --- | --- | --- | --- | --- | --- | --- | --- | --- | --- | --- | --- | --- | --- | --- | --- | --- | --- | --- | --- | --- | --- | --- | --- | --- | --- | --- | --- | --- | --- | --- | --- | --- | --- | --- | --- | --- | --- | --- | --- | --- | --- | --- | --- | --- | --- | --- | --- | --- | --- | --- | --- | --- | --- | --- | --- | --- | --- | --- | --- | --- | --- | --- | --- | --- | --- | --- | --- | --- | --- | --- | --- | --- | --- | --- | --- | --- | --- | --- | --- | --- | --- | --- | --- | --- | --- | --- | --- | --- | --- | --- | --- | --- | --- | --- | --- | --- | --- | --- | --- | --- | --- | --- | --- | --- | --- | --- | --- | --- | --- | --- | --- | --- | --- | --- | --- | --- | --- | --- | --- | --- | --- | --- | --- | --- | --- | --- | --- | --- | --- | --- | --- | --- | --- | --- | --- | --- | --- | --- | --- | --- | --- | --- | --- | --- | --- | --- | --- | --- | --- | --- | --- | --- | --- | --- | --- | --- | --- | --- | --- | --- | --- | --- | --- | --- | --- | --- | --- | --- | --- | --- | --- | --- | --- | --- | --- | --- | --- | --- | --- | --- | --- | --- | --- | --- | --- | --- | --- | --- | --- | --- | --- | --- | --- | --- | --- | --- | --- | --- | --- | --- | --- | --- | --- | --- | --- | --- | --- | --- | --- | --- | --- | --- | --- | --- | --- | --- | --- | --- | --- | --- | --- | --- | --- | --- | --- | --- | --- | --- | --- | --- | --- | --- | --- | --- | --- | --- | --- | --- | --- | --- | --- | --- | --- | --- | --- | --- | --- | --- | --- | --- | --- | --- | --- | --- | --- | --- | --- | --- | --- | --- | --- | --- | --- | --- | --- | --- | --- | --- | --- | --- | --- | --- | --- | --- | --- | --- | --- | --- | --- | --- | --- | --- | --- | --- | --- | --- | --- | --- | --- | --- | --- | --- | --- | --- | --- | --- | --- | --- | --- | --- | --- | --- | --- | --- | --- | --- | --- | --- | --- | --- | --- | --- | --- | --- | --- | --- | --- | --- | --- | --- | --- | --- | --- | --- | --- | --- | --- | --- | --- | --- | --- | --- | --- | --- | --- | --- | --- | --- | --- | --- | --- | --- | --- | --- | --- | --- | --- | --- | --- | --- | --- | --- | --- | --- | --- | --- | --- | --- | --- | --- | --- | --- | --- | --- | --- | --- | --- | --- | --- | --- | --- | --- | --- | --- | --- | --- | --- | --- | --- | --- | --- | --- | --- | --- | --- | --- | --- | --- | --- | --- | --- | --- | --- | --- | --- | --- | --- | --- | --- | --- | --- | --- | --- | --- | --- | --- | --- | --- | --- | --- | --- | --- | --- | --- | --- |
| HSPC\_025 | 0.0 | 0.0 | 0.0 | 5.392129 | 8.852337 | 0.000000 | 2.614548 | 0.0 | 0.000000 | 0.0 | 6.711045 | 7.977803 | 8.117451 | 0.0 | 0.000000 | 8.195180 | 7.977803 | 0.000000 | 9.173640 | 0.0 | 12.912375 | 8.427644 | 7.839405 | 0.0 | 1.189716 | 0.0 | 0.0 | 0.000000 | 0.000000 | 5.935712 | 0.0 | 8.021134 | 0.000000 | 7.309000 | 10.293674 | 0.000000 | 0.000000 | 2.275971 | 0.000000 | 0.0 | 9.014787 | 0.0 | 0.000000 | 0.000000 | 0.000000 | 1.832751 | 8.774285 | 1.189716 | 1.189716 | 1.832751 | 0.0 | 3.118770 | 7.285499 | 0.0 | 7.135838 | 8.744431 | 0.000000 | 0.0 | 8.852337 | 0.0 | 0.0 | 0.0 | 1.832751 | 1.189716 | 0.0 | 0.0 | 0.0 | 1.189716 | 0.0 | 0.000000 | 7.225016 | 8.876132 | 7.940672 | 6.184685 | 1.189716 | 0.0 | 8.691769 | 1.189716 | 0.0 | 3.491677 | 7.161885 | 0.0 | 0.0 | 0.0 | 1.832751 | 6.779938 | 8.490658 | 11.925211 | 7.410259 | 3.317239 | 0.000000 | 10.416407 | 4.242991 | 2.275971 | 1.832751 | 1.189716 | 0.000000 | 7.894815 | 0.000000 | 9.681046 | 1.189716 | 0.0 | 0.0 | 9.723202 | 4.141925 | 0.000000 | 0.0 | 7.574401 | 7.174734 | 2.275971 | 0.0 | 2.614548 | 0.000000 | 2.888576 | 0.000000 | 7.806760 | 8.268934 | 3.491677 | 0.0 | 0.0 | 0.0 | 0.0 | 8.585122 | 2.888576 | 2.614548 | 0.000000 | 8.344797 | 6.461289 | 7.285499 | 0.000000 | 7.593666 | 6.657127 | 0.0 | 2.275971 | 8.655561 | 9.077708 | 0.000000 | 1.832751 | 1.189716 | 7.695253 | 10.437884 | 1.832751 | 7.814991 | 0.0 | 0.0 | 1.189716 | 5.935712 | 4.588530 | 2.275971 | 9.025466 | 0.0 | 1.189716 | 0.000000 | 0.0 | 0.0 | 3.118771 | 1.189716 | 1.189716 | 7.161885 | 0.0 | 0.0 | 0.0 | 0.000000 | 10.103505 | 0.0 | 0.0 | 0.0 | 2.888576 | 0.000000 | 0.00000 | 0.000000 | 7.977803 | 6.675324 | 0.000000 | 0.000000 | 7.054751 | 0.000000 | 0.000000 | 0.0 | 0.000000 | 0.000000 | 9.101455 | 7.040782 | 9.449536 | 0.000000 | 0.000000 | 8.310278 | 8.495788 | 0.0 | 0.000000 | 2.888576 | 8.687292 | 7.940672 | 0.0 | 1.189716 | 11.307754 | 7.781782 | 7.894815 | 8.361752 | 0.000000 | 2.888576 | 9.067410 | 4.509590 | 8.310278 | 0.0 | 0.000000 | 0.000000 | 0.000000 | 0.000000 | 7.343549 | 0.000000 | 5.671352 | 7.564672 | 10.551846 | 6.763021 | 0.000000 | 1.189716 | 0.000000 | 0.000000 | 1.189716 | 9.272583 | 0.000000 | 1.189716 | 8.021134 | 8.150349 | 2.275971 | 0.000000 | 7.747791 | 8.130700 | 3.647281 | 0.0 | 7.593666 | 1.832751 | 1.189716 | 7.790156 | 7.421084 | 0.000000 | 0.000000 | 6.779938 | 2.275971 | 6.079295 | 0.000000 | 2.275971 | 2.888576 | 0.0 | 9.014787 | 1.189716 | 8.182512 | 7.977803 | 4.141925 | 1.189716 | 9.826553 | 0.000000 | 9.822478 | 8.594715 | 0.0 | 6.796659 | 0.0 | 0.0 | 7.273603 | 0.000000 | 4.141925 | 1.189716 | 6.845691 | 0.000000 | 1.189716 | 6.923879 | 2.888576 | 5.776244 | 0.000000 | 0.000000 | 3.118771 | 8.475161 | 0.000000 | 0.0 | 10.807313 | 7.122636 | 0.000000 | 6.711045 | 0.0 | 4.242991 | 7.525084 | 2.275971 | 0.0 | 4.033241 | 9.167225 | 0.000000 | 3.317239 | 7.747791 | 0.000000 | 9.816341 | 0.000000 | 0.000000 | 0.000000 | 1.189716 | 0.0 | 10.088305 | 7.955639 | 0.000000 | 0.00000 | 0.000000 | 0.0 | 11.315027 | 7.631440 | 0.000000 | 0.000000 | 0.0 | 1.189716 | 6.079295 | 8.356122 | 7.554876 | 9.390178 | 0.000000 | 7.887028 | 1.832751 | 9.681046 | 9.032541 | 10.105184 | 0.0 | 6.282898 | 6.502641 | 3.787723 | 0.0 | 0.0 | 7.730490 | 0.000000 | 7.992392 | 7.902559 | 8.274914 | 7.668250 | 9.339726 | 0.0 | 8.063203 | 9.710022 | 1.189716 | 4.337438 | 0.0 | 7.593666 | 7.320609 | 0.0 | 0.0 | 1.832751 | 7.871328 | 7.584065 | 1.189716 | 1.832751 | 10.034692 | 0.000000 | 1.832751 | 3.317239 | 0.0 | 5.347433 | 0.0 | 0.0 | 0.0 | 0.000000 | 5.634622 | 7.554876 | 6.502641 | 0.0 | 7.640730 | 9.278547 | 8.971261 | 0.0 | 0.000000 | 7.012433 | 0.000000 | 8.035294 | 1.832751 | 9.438924 | 7.879200 | 0.0 | 6.159051 | 0.000000 | 0.0 | 8.028232 | 0.000000 | 8.156838 | 0.0 | 0.000000 | 2.888576 | 1.832751 | 8.400554 | 0.0 | 4.426081 | 1.832751 | 0.000000 | 0.0 | 9.345421 | 0.000000 | 0.000000 | 0.0 | 8.400554 | 3.118771 | 0.0 | 8.989556 | 7.174734 | 7.704143 | 7.273603 | 0.000000 | 8.433001 | 4.337438 | 0.000000 | 8.124090 | 9.656065 | 0.000000 | 0.0 | 7.095865 | 0.0 | 8.454235 | 0.000000 | 6.132952 | 7.040782 | 0.0 | 0.000000 | 5.905197 | 0.0 | 0.000000 | 1.189716 | 7.839405 | 5.809578 | 0.0 | 0.0 | 7.297297 | 3.491677 | 0.000000 | 8.490658 | 0.0 | 0.000000 | 0.0 | 0.000000 | 0.000000 | 11.130318 | 8.207737 | 0.000000 | 8.993188 | 0.000000 | 9.521714 | 2.614548 | 9.882450 | 0.0 | 0.0 | 9.506549 | 2.275971 | 3.915699 | 0.0 | 6.923879 | 9.690023 | 1.189716 | 0.0 | 0.000000 | 0.0 | 0.000000 | 7.285499 | 7.631440 | 3.118771 | 2.275971 | 0.0 | 7.161885 | 8.926381 | 0.0 | 1.189716 | 0.000000 | 9.342576 | 10.287772 | 5.742120 | 2.275971 | 8.350471 | 8.744431 | 7.847452 | 2.614548 | 0.0 | 1.189716 | 9.812235 | 3.915699 | 0.000000 | 1.189716 | 1.189716 | 0.000000 | 6.796659 | 7.695253 | 7.659135 | 0.000000 | 0.0 | 9.716627 | 7.320609 | 0.0 | 0.0 | 9.428233 | 0.000000 | 1.189716 | 6.983514 | 1.189716 | 0.000000 | 8.536176 | 0.000000 | 8.156838 | 0.0 | 1.832751 | 0.000000 | 0.000000 | 0.0 | 0.0 | 7.535083 | 0.0 | 0.000000 | 7.515015 | 8.274914 | 0.00000 | 4.242991 | 6.711045 | 0.0 | 0.0 | 0.0 | 7.095865 | 0.0 | 0.000000 | 0.0 | 0.000000 | 9.000423 | 7.712979 | 5.045572 | 0.0 | 0.0 | 7.871328 | 1.189716 | 0.0 | 8.774285 | 0.0 | 0.000000 | 8.256901 | 9.091325 | 9.254540 | 7.054751 | 2.888576 | 1.832751 | 0.000000 | 7.399354 | 0.0 | 0.000000 | 9.681046 | 0.0 | 0.0 | 8.570612 | 0.000000 | 6.418716 | 1.832751 | 1.189716 | 1.832751 | 0.0 | 0.000000 | 0.0 | 0.000000 | 0.000000 | 9.903830 | 1.189716 | 1.189716 | 1.189716 | 8.459496 | 0.000000 | 0.000000 | 1.832751 | 0.0 | 0.0 | 1.832751 | 1.189716 | 5.935712 | 0.0 | 0.0 | 1.189716 | 2.275971 | 1.189716 | 0.00000 | 0.0 | 2.275971 | 0.000000 | 1.189716 | 9.707812 | 0.000000 | 0.000000 | 0.0 | 8.786894 | 8.978607 | 8.691769 | 0.0 | 0.000000 | 9.785260 | 1.832751 | 8.967574 | 0.000000 | 0.0 | 0.0 | 6.983514 | 0.0000 | 6.779938 | 3.491677 | 0.000000 | 2.614548 | 0.0 | 8.782703 | 0.000000 | 9.004027 | 0.000000 | 4.802330 | 4.734524 | 3.491677 | 1.189716 | 7.320609 | 0.000000 | 9.299230 | 0.0 | 9.501458 | 1.189716 | 8.555954 | 0.000000 | 15.299863 | 0.000000 | 0.0 | 1.832751 | 9.365173 | 0.0 | 6.106372 | 9.656065 | 8.176136 | 6.258964 | 2.275971 | 9.118180 | 0.0 | 3.491677 | 9.901899 | 0.000000 | 0.0 | 9.362368 | 0.0 | 1.189716 | 7.764886 | 0.000000 | 1.189716 | 0.0 | 0.000000 | 0.0 | 5.392129 | 2.275971 | 0.000000 | 0.0 | 0.000000 | 0.000000 | 0.0 | 8.226370 | 0.000000 | 9.475731 | 2.275971 | 1.832751 | 0.0 | 1.189716 | 1.832751 | 3.491677 | 1.189716 | 0.0 | 0.0 | 7.148920 | 0.0 | 1.832751 | 0.0 | 9.018355 | 0.000000 | 8.280868 | 0.0 | 0.000000 | 9.322510 | 8.536176 | 0.000000 | 8.722721 | 7.756364 | 9.457444 | 9.874597 | 0.000000 | 1.189716 | 1.832751 | 1.189716 | 6.763021 | 0.000000 | 1.189716 | 3.317239 | 8.887885 | 1.189716 | 1.189716 | 1.832751 | 8.922578 | 0.00000 | 0.0 | 2.275971 | 2.888576 | 0.000000 | 1.189716 | 9.454813 | 0.0 | 6.542841 | 8.117451 | 1.832751 | 8.378509 | 0.0 | 0.0 | 0.0 | 0.000000 | 8.627797 | 1.189716 | 1.189716 | 2.275971 | 11.533395 | 0.0 | 5.842161 | 0.000000 | 0.0 | 4.663373 | 7.814991 | 0.0 | 0.0 | 1.189716 | 1.189716 | 8.256901 | 8.333382 | 1.189716 | 9.164007 | 0.0 | 0.0 | 7.721761 | 6.418716 | 7.879200 | 7.535083 | 6.877476 | 0.000000 | 0.000000 | 6.968835 | 0.0 | 9.384659 | 0.000000 | 0.0 | 0.0 | 9.351091 | 0.000000 | 3.118771 | 9.428233 | 0.0 | 7.545014 | 8.872193 | 0.0 | 0.000000 | 6.693295 | 0.0 | 0.0 | 0.000000 | 1.189716 | 0.0 | 0.000000 | 2.275971 | 0.000000 | 9.124817 | 0.0 | 1.832751 | 6.581951 | 0.0 | 0.000000 | 0.0 | 8.464736 | 0.0 | 1.832751 | 8.744431 | 11.084242 | 1.189716 | 10.748367 | 7.525084 | 0.0 | 0.0 | 0.0 | 9.263590 | 0.0 | 0.0 | 1.832751 | 8.832203 | 7.012433 | 1.832751 | 0.0 | 0.0 | 10.351401 | 0.000000 | 0.000000 | 8.021134 | 2.275971 | 9.658353 | 0.0 | 9.000423 | 1.189716 | 0.000000 | 0.000000 | 0.0 | 3.118771 | 0.0 | 0.0 | 8.531189 | 8.753024 | 0.0 | 7.421084 | 0.0 | 0.0 | 3.118771 | 8.367359 | 5.347433 | 0.0 | 9.160781 | 2.614548 | 8.327641 | 0.0 | 0.0 | 0.0 | 4.033241 | 6.693295 | 7.187470 | 9.514152 | 8.213975 | 0.0 | 0.0 | 0.0 | 0.0 | 6.209872 | 0.000000 | 3.317239 | 0.000000 | 2.614548 | 7.122636 | 9.491221 | 1.189716 | 1.189716 | 0.0 | 0.0 | 0.0 | 0.000000 | 8.169733 | 11.876887 | 0.000000 | 8.316089 | 0.0 | 0.000000 | 0.000000 | 0.000000 | 2.888576 | 0.0 | 0.000000 | 1.189716 | 6.184685 | 1.189716 | 5.558232 | 2.614548 | 7.082290 | 8.083785 | 0.0 | 0.000000 | 0.0 | 8.989556 | 9.202161 | 3.317239 | 0.000000 | 0.000000 | 0.0 | 8.832203 | 1.189716 | 8.459496 | 0.0 | 7.695253 | 9.989955 | 0.0 | 0.000000 | 10.686910 | 0.000000 | 2.275971 | 1.189716 | 0.000000 | 0.0 | 0.0 | 7.068586 | 0.0 | 7.739166 | 0.0 | 0.0 | 1.189716 | 0.000000 | 9.892208 | 7.970453 | 1.189716 | 0.0 | 0.0 | 0.0 | 7.484379 | 9.141275 | 0.0 | 0.0 | 0.0 | 0.000000 | 2.275971 | 8.511066 | 8.244766 | 10.309781 | 0.000000 | 7.068586 | 5.518464 | 7.332125 | 0.0 | 8.832203 | 1.189716 | 5.965594 | 0.0 | 3.118771 | 0.0 | 8.384051 | 0.0 | 2.275971 | 8.238660 | 0.0 | 4.588530 | 1.189716 | 0.000000 | 8.411450 | 6.877476 | 7.421084 | 1.832751 | 6.258964 | 0.000000 | 5.842161 | 0.000000 | 11.228325 | 1.189716 | 5.253659 | 1.189716 | 0.0 | 6.923879 | 0.000000 | 1.832751 | 1.832751 | 3.118771 | 0.00000 | 9.157549 | 0.000000 | 0.000000 | 0.0 | 8.433001 | 7.442493 | 7.261610 | 0.000000 | 7.704143 | 0.000000 | 0.000000 | 9.588054 | 1.189716 | 0.000000 | 11.117064 | 2.275971 | 9.563837 | 0.0 | 8.967574 | 6.893110 | 8.575464 | 1.832751 | 1.189716 | 8.673779 | 0.0 | 9.114851 | 3.118771 | 0.000000 | 0.0 | 0.0 | 0.000000 | 8.316089 | 0.0 | 10.128487 | 6.968835 | 7.174734 | 0.0 | 8.056275 | 1.189716 | 7.442493 | 1.189716 | 8.848332 | 0.0 | 9.296293 | 0.000000 | 7.747791 | 2.275971 | 0.0 | 1.189716 | 0.000000 | 1.832751 | 7.730490 | 0.000000 | 7.999631 | 7.535083 | 9.438924 | 0.000000 | 1.189716 | 0.000000 | 7.212608 | 9.084533 | 1.189716 | 0.000000 | 11.455247 | 0.0 | 0.0 | 1.189716 | 0.000000 | 0.000000 | 6.418716 | 0.000000 | 0.000000 | 0.000000 | 8.748734 | 1.189716 | 0.0 | 1.189716 | 6.159051 | 7.494663 | 8.718340 | 1.189716 | 7.649962 | 0.0 | 2.614548 | 1.832751 | 6.306441 | 0.0 | 0.0 | 0.0 | 0.000000 | 0.0 | 7.135838 | 0.000000 | 5.253659 | 4.337438 | 8.395074 | 0.000000 | 8.350471 | 9.714428 | 8.526184 | 7.649962 | 0.0 | 8.718340 | 0.000000 | 10.165970 | 8.137279 | 0.000000 | 7.309000 | 1.189716 | 0.000000 | 0.0 | 1.832751 | 8.641746 | 7.992392 | 0.000000 | 9.331144 | 7.431828 | 0.0 | 1.189716 | 9.334011 | 9.403884 | 0.000000 | 1.189716 | 9.007623 | 9.390178 | 0.0 | 0.000000 | 8.028232 | 0.0 | 0.000000 | 7.721761 | 0.000000 | 0.000000 | 1.189716 | 1.189716 | 6.581951 | 8.475161 | 7.054751 | 0.000000 | 0.0 | 0.000000 | 6.939021 | 9.742748 | 6.829531 | 7.992392 | 0.0 | 8.130700 | 0.0 | 0.0 | 1.189716 | 9.214658 | 7.887028 | 0.000000 | 7.399354 | 2.275971 | 1.189716 | 0.0 | 6.763021 | 0.0 | 0.0 | 10.830842 | 0.000000 | 0.0 | 1.832751 | 0.0 | 0.0 | 8.608986 | 8.316089 | 0.0 | 1.189716 | 0.000000 | 9.173640 | 5.809578 | 0.0 | 0.000000 | 9.414757 | 7.612677 | 0.0 | 0.0 | 10.214998 | 6.374848 | 7.545014 | 6.051698 | 0.0 | 0.0 | 9.921090 | 8.860312 | 0.000000 | 8.815891 | 9.269591 | 7.739166 | 7.545014 | 0.0 | 0.000000 | 9.953140 | 10.011602 | 0.0 | 2.275971 | 0.000000 | 8.110780 | 0.000000 | 8.883978 | 7.273603 | 9.449536 | 0.000000 | 0.000000 | 0.0 | 6.282898 | 1.189716 | 9.245434 | 0.0 | 0.0 | 0.0 | 0.0 | 0.0 | 0.000000 | 0.0 | 1.189716 | 0.0 | 2.614548 | 0.000000 | 0.0 | 0.000000 | 1.189716 | 9.263590 |
| HSPC\_031 | 0.0 | 0.0 | 0.0 | 0.686872 | 7.637939 | 0.000000 | 6.838205 | 0.0 | 0.000000 | 0.0 | 8.951078 | 8.584765 | 0.000000 | 0.0 | 0.000000 | 6.956259 | 7.579348 | 0.000000 | 4.175851 | 0.0 | 13.622584 | 7.800662 | 0.000000 | 0.0 | 0.686872 | 0.0 | 0.0 | 0.686872 | 0.000000 | 0.000000 | 0.0 | 7.018651 | 1.150286 | 3.253543 | 7.387780 | 0.686872 | 0.000000 | 0.000000 | 0.000000 | 0.0 | 6.348589 | 0.0 | 0.686872 | 0.686872 | 0.000000 | 0.000000 | 2.827390 | 8.409573 | 0.686872 | 1.782055 | 0.0 | 8.765434 | 8.059983 | 0.0 | 8.638735 | 9.676164 | 0.686872 | 0.0 | 1.782055 | 0.0 | 0.0 | 0.0 | 1.150286 | 1.150286 | 0.0 | 0.0 | 0.0 | 0.000000 | 0.0 | 0.000000 | 9.235683 | 10.222019 | 2.946297 | 0.000000 | 0.000000 | 0.0 | 9.531507 | 0.000000 | 0.0 | 1.500480 | 0.686872 | 0.0 | 0.0 | 0.0 | 0.000000 | 0.000000 | 0.000000 | 11.665664 | 9.216587 | 5.672502 | 0.000000 | 9.484386 | 0.000000 | 1.150286 | 0.000000 | 0.000000 | 0.000000 | 0.000000 | 0.000000 | 6.991254 | 0.000000 | 0.0 | 0.0 | 9.159273 | 3.158217 | 7.403453 | 0.0 | 8.372889 | 8.587055 | 1.500480 | 0.0 | 8.401790 | 0.686872 | 2.017546 | 0.686872 | 0.686872 | 1.500480 | 7.574740 | 0.0 | 0.0 | 0.0 | 0.0 | 7.527841 | 3.342958 | 0.000000 | 1.150286 | 8.598449 | 1.782055 | 1.782055 | 0.000000 | 0.686872 | 1.150286 | 0.0 | 2.219938 | 2.219938 | 9.404856 | 0.686872 | 1.150286 | 0.686872 | 2.017546 | 0.000000 | 0.686872 | 0.000000 | 0.0 | 0.0 | 7.824144 | 7.148337 | 2.827390 | 2.397399 | 9.564413 | 0.0 | 6.623022 | 0.000000 | 0.0 | 0.0 | 2.397399 | 7.032157 | 0.000000 | 0.000000 | 0.0 | 0.0 | 0.0 | 0.000000 | 11.892010 | 0.0 | 0.0 | 0.0 | 1.782055 | 0.000000 | 0.00000 | 1.150286 | 0.000000 | 7.828021 | 1.150286 | 5.925034 | 0.000000 | 0.686872 | 0.000000 | 0.0 | 0.000000 | 0.686872 | 8.915083 | 0.000000 | 9.006865 | 8.155637 | 0.000000 | 9.640235 | 2.397399 | 0.0 | 0.000000 | 2.017546 | 9.543345 | 0.686872 | 0.0 | 0.000000 | 9.419062 | 9.750561 | 1.150286 | 0.686872 | 0.686872 | 6.549791 | 8.233698 | 3.653748 | 1.500480 | 0.0 | 0.000000 | 0.000000 | 0.000000 | 0.000000 | 8.662811 | 0.000000 | 3.253543 | 0.000000 | 2.219938 | 9.646834 | 0.000000 | 0.000000 | 0.000000 | 0.000000 | 0.000000 | 9.962022 | 0.686872 | 8.785511 | 9.802712 | 8.826781 | 0.686872 | 0.000000 | 8.545278 | 2.697797 | 9.449579 | 0.0 | 8.201186 | 0.000000 | 0.000000 | 8.900430 | 2.397399 | 1.500480 | 0.000000 | 0.686872 | 3.582099 | 0.000000 | 0.000000 | 3.506705 | 2.219938 | 0.0 | 10.152610 | 1.150286 | 6.348589 | 0.000000 | 0.000000 | 0.000000 | 7.780798 | 1.150286 | 1.150286 | 8.951078 | 0.0 | 0.000000 | 0.0 | 0.0 | 0.000000 | 0.000000 | 0.000000 | 0.000000 | 6.293577 | 8.896743 | 7.116992 | 0.686872 | 8.445345 | 8.842198 | 0.000000 | 0.686872 | 7.045538 | 1.782055 | 0.000000 | 0.0 | 8.900430 | 0.686872 | 1.500480 | 9.076959 | 0.0 | 0.000000 | 2.219938 | 9.871007 | 0.0 | 8.938581 | 2.017546 | 6.977358 | 2.697797 | 1.150286 | 8.195195 | 10.778556 | 0.000000 | 0.000000 | 0.000000 | 2.017546 | 0.0 | 10.784055 | 0.000000 | 0.000000 | 0.00000 | 5.004467 | 0.0 | 5.565109 | 3.506705 | 0.000000 | 0.000000 | 0.0 | 0.000000 | 5.706594 | 0.686872 | 7.135881 | 7.615685 | 0.000000 | 9.234223 | 0.000000 | 9.659944 | 1.500480 | 8.627656 | 0.0 | 0.000000 | 0.000000 | 2.017546 | 0.0 | 0.0 | 9.060576 | 0.000000 | 8.904107 | 1.782055 | 6.942020 | 8.069839 | 8.233698 | 0.0 | 4.631208 | 9.284465 | 6.783355 | 7.214989 | 0.0 | 0.000000 | 0.000000 | 0.0 | 0.0 | 7.377236 | 0.686872 | 0.000000 | 0.686872 | 0.686872 | 10.663397 | 0.000000 | 0.000000 | 0.000000 | 0.0 | 7.255855 | 0.0 | 0.0 | 0.0 | 0.686872 | 1.150286 | 4.021900 | 0.000000 | 0.0 | 2.219938 | 3.849535 | 2.827391 | 0.0 | 0.000000 | 0.000000 | 8.467882 | 9.385257 | 8.095797 | 10.225698 | 7.393023 | 0.0 | 9.179137 | 0.000000 | 0.0 | 1.782055 | 5.637585 | 8.561668 | 0.0 | 5.449074 | 9.228369 | 4.223722 | 2.946297 | 0.0 | 6.380618 | 9.852094 | 0.000000 | 0.0 | 9.311023 | 0.000000 | 0.686872 | 0.0 | 0.000000 | 7.273021 | 0.0 | 1.500480 | 9.495397 | 7.209055 | 3.427154 | 0.000000 | 10.735345 | 6.022986 | 0.000000 | 7.565481 | 2.555402 | 2.397399 | 0.0 | 8.570951 | 0.0 | 8.009667 | 8.874422 | 1.150286 | 9.864415 | 0.0 | 0.000000 | 6.984322 | 0.0 | 0.000000 | 0.000000 | 7.694245 | 0.000000 | 0.0 | 0.0 | 0.000000 | 2.397399 | 0.686872 | 8.259764 | 0.0 | 0.000000 | 0.0 | 0.686872 | 0.000000 | 9.967304 | 8.682216 | 0.000000 | 4.520574 | 5.723342 | 8.989663 | 1.782055 | 2.827391 | 0.0 | 0.0 | 8.030005 | 3.342958 | 0.000000 | 0.0 | 8.137007 | 2.017546 | 0.686872 | 0.0 | 0.686872 | 0.0 | 0.000000 | 8.477787 | 8.566318 | 6.502060 | 1.500480 | 0.0 | 0.000000 | 1.500480 | 0.0 | 0.000000 | 0.686872 | 2.397399 | 3.253543 | 8.393966 | 2.017546 | 8.251127 | 7.719509 | 0.000000 | 0.686872 | 0.0 | 1.500480 | 9.514768 | 0.000000 | 0.000000 | 0.686872 | 8.533455 | 0.686872 | 7.664200 | 8.304972 | 1.150286 | 0.000000 | 0.0 | 10.264835 | 0.000000 | 0.0 | 0.0 | 9.858742 | 9.715412 | 8.730652 | 1.500480 | 6.462704 | 0.686872 | 4.595264 | 0.000000 | 10.762946 | 0.0 | 0.686872 | 0.000000 | 0.000000 | 0.0 | 0.0 | 8.989663 | 0.0 | 0.686872 | 0.686872 | 7.892380 | 0.00000 | 1.782055 | 2.697797 | 0.0 | 0.0 | 0.0 | 2.219938 | 0.0 | 2.697797 | 0.0 | 0.686872 | 8.785511 | 6.830496 | 7.317821 | 0.0 | 0.0 | 7.659856 | 0.000000 | 0.0 | 2.219938 | 0.0 | 0.000000 | 2.219938 | 9.460858 | 3.342958 | 8.124452 | 8.716082 | 0.000000 | 7.672848 | 1.500480 | 0.0 | 2.017546 | 8.830651 | 0.0 | 0.0 | 0.686872 | 0.000000 | 8.568637 | 6.101957 | 0.000000 | 10.293858 | 0.0 | 0.686872 | 0.0 | 8.186162 | 0.000000 | 3.427154 | 0.000000 | 0.000000 | 6.076110 | 4.075063 | 0.686872 | 0.686872 | 0.000000 | 0.0 | 0.0 | 7.065378 | 0.000000 | 5.387358 | 0.0 | 0.0 | 0.000000 | 2.017546 | 0.000000 | 0.00000 | 0.0 | 2.697797 | 7.877493 | 5.672502 | 11.297833 | 0.686872 | 2.697797 | 0.0 | 1.500480 | 7.999391 | 1.500480 | 0.0 | 0.686872 | 5.967830 | 2.827391 | 8.502254 | 0.686872 | 0.0 | 0.0 | 8.161794 | 4.0219 | 8.753254 | 8.177073 | 0.686872 | 3.722005 | 0.0 | 5.865923 | 8.880034 | 2.219938 | 0.000000 | 2.397399 | 0.000000 | 1.500480 | 7.005018 | 9.698571 | 0.686872 | 1.150286 | 0.0 | 9.231299 | 0.000000 | 5.449074 | 6.492322 | 14.849717 | 0.686872 | 0.0 | 0.000000 | 2.697797 | 0.0 | 1.500480 | 3.722005 | 8.149453 | 8.417314 | 0.686872 | 8.817061 | 0.0 | 7.209055 | 8.634314 | 0.686872 | 0.0 | 5.925034 | 0.0 | 0.686872 | 8.972252 | 0.000000 | 7.839589 | 0.0 | 0.000000 | 0.0 | 8.354193 | 0.000000 | 0.000000 | 0.0 | 3.158217 | 0.686872 | 0.0 | 9.163881 | 5.084431 | 9.607904 | 0.686872 | 8.894896 | 0.0 | 0.686872 | 0.000000 | 0.000000 | 0.686872 | 0.0 | 0.0 | 1.500480 | 0.0 | 0.000000 | 0.0 | 6.861088 | 0.000000 | 9.492957 | 0.0 | 8.775508 | 9.517171 | 0.000000 | 7.928939 | 2.397399 | 0.000000 | 3.056145 | 11.210691 | 0.686872 | 9.372041 | 6.511734 | 9.587467 | 0.000000 | 9.053970 | 1.500480 | 9.685810 | 5.184588 | 0.000000 | 0.000000 | 0.000000 | 9.000010 | 0.00000 | 0.0 | 4.700515 | 8.140128 | 0.000000 | 1.500480 | 8.654102 | 0.0 | 0.000000 | 9.431856 | 3.909308 | 3.506705 | 0.0 | 0.0 | 0.0 | 5.655149 | 8.634314 | 1.500480 | 6.861088 | 9.823251 | 10.901846 | 0.0 | 0.000000 | 0.000000 | 0.0 | 1.782055 | 9.126594 | 0.0 | 0.0 | 0.000000 | 8.728580 | 2.219938 | 4.595264 | 0.000000 | 9.771850 | 0.0 | 0.0 | 8.789493 | 0.000000 | 1.782055 | 9.018787 | 3.787179 | 8.822902 | 0.000000 | 5.322884 | 0.0 | 2.397399 | 1.150286 | 0.0 | 0.0 | 10.055096 | 0.686872 | 0.000000 | 8.612002 | 0.0 | 3.056145 | 8.751214 | 0.0 | 0.000000 | 9.337102 | 0.0 | 0.0 | 0.000000 | 0.000000 | 0.0 | 0.000000 | 6.807118 | 0.000000 | 8.318813 | 0.0 | 0.000000 | 1.150286 | 0.0 | 1.150286 | 0.0 | 0.686872 | 0.0 | 1.150286 | 7.925325 | 11.145000 | 0.686872 | 10.561133 | 8.130743 | 0.0 | 0.0 | 0.0 | 9.722719 | 0.0 | 0.0 | 2.946297 | 8.765435 | 0.000000 | 0.000000 | 0.0 | 0.0 | 1.150286 | 7.267321 | 0.000000 | 9.810572 | 2.219938 | 9.793820 | 0.0 | 9.333015 | 4.700515 | 5.184588 | 0.000000 | 0.0 | 1.500480 | 0.0 | 0.0 | 1.782055 | 7.788776 | 0.0 | 3.342958 | 0.0 | 0.0 | 3.253543 | 6.807118 | 0.000000 | 0.0 | 7.537344 | 8.820957 | 8.288184 | 0.0 | 0.0 | 0.0 | 0.000000 | 7.129612 | 0.686872 | 6.640763 | 6.472644 | 0.0 | 0.0 | 0.0 | 0.0 | 0.000000 | 0.000000 | 0.000000 | 0.000000 | 3.582099 | 2.017546 | 8.043406 | 0.000000 | 0.000000 | 0.0 | 0.0 | 0.0 | 1.150286 | 7.261599 | 11.510276 | 0.686872 | 2.555402 | 0.0 | 0.000000 | 0.000000 | 0.000000 | 0.686872 | 0.0 | 0.000000 | 7.655500 | 0.000000 | 4.075063 | 0.000000 | 9.710171 | 1.782055 | 7.877493 | 0.0 | 0.000000 | 0.0 | 9.001726 | 9.143804 | 6.009394 | 0.000000 | 1.150286 | 0.0 | 10.457334 | 0.686872 | 8.482714 | 0.0 | 7.220899 | 5.850759 | 0.0 | 0.000000 | 9.467087 | 7.350532 | 1.782055 | 0.000000 | 3.158217 | 0.0 | 0.0 | 8.994845 | 0.0 | 8.934992 | 0.0 | 0.0 | 0.000000 | 0.686872 | 6.511734 | 2.219938 | 0.686872 | 0.0 | 0.0 | 0.0 | 0.686872 | 0.686872 | 0.0 | 0.0 | 0.0 | 0.000000 | 0.000000 | 10.249019 | 9.670777 | 9.728952 | 2.555402 | 1.500480 | 9.080214 | 6.807118 | 0.0 | 8.846027 | 0.000000 | 0.000000 | 0.0 | 2.397399 | 0.0 | 5.278242 | 0.0 | 2.397399 | 7.429204 | 0.0 | 7.740229 | 0.686872 | 0.000000 | 2.219938 | 9.839666 | 8.596177 | 0.000000 | 0.000000 | 0.000000 | 0.000000 | 0.000000 | 9.762764 | 1.150286 | 0.686872 | 0.000000 | 0.0 | 0.686872 | 0.000000 | 0.000000 | 1.782055 | 9.107582 | 0.00000 | 2.697797 | 0.686872 | 10.531721 | 0.0 | 1.782055 | 1.150286 | 5.255393 | 0.686872 | 0.686872 | 0.000000 | 0.000000 | 3.506705 | 4.919811 | 0.000000 | 0.686872 | 5.565109 | 9.814485 | 0.0 | 9.297106 | 5.689649 | 8.781518 | 0.000000 | 9.254531 | 7.154525 | 0.0 | 2.555402 | 7.873747 | 1.150286 | 0.0 | 0.0 | 1.150286 | 8.878166 | 0.0 | 1.150286 | 9.615775 | 1.500480 | 0.0 | 1.782055 | 0.000000 | 8.959939 | 6.853501 | 9.008575 | 0.0 | 4.358484 | 0.686872 | 1.782055 | 2.827391 | 0.0 | 0.000000 | 6.623022 | 0.000000 | 0.000000 | 0.000000 | 1.150286 | 5.672502 | 2.017546 | 0.000000 | 0.686872 | 0.000000 | 6.248005 | 4.595264 | 1.782055 | 6.876145 | 10.860805 | 0.0 | 0.0 | 1.150286 | 1.150286 | 0.686872 | 8.236617 | 0.000000 | 8.697130 | 0.000000 | 0.686872 | 0.000000 | 0.0 | 8.470365 | 0.686872 | 9.470811 | 6.152300 | 0.000000 | 0.000000 | 0.0 | 2.697797 | 5.058266 | 8.477787 | 0.0 | 0.0 | 0.0 | 8.164863 | 0.0 | 1.150286 | 0.000000 | 7.513468 | 0.000000 | 5.739898 | 5.655149 | 9.317932 | 6.701193 | 0.000000 | 8.401790 | 0.0 | 1.150286 | 0.686872 | 8.460409 | 0.686872 | 0.000000 | 2.946297 | 0.000000 | 0.686872 | 0.0 | 0.686872 | 9.285875 | 1.782055 | 5.208578 | 9.719592 | 0.000000 | 0.0 | 9.479464 | 6.359344 | 8.380830 | 0.686872 | 0.000000 | 9.978684 | 9.464599 | 0.0 | 0.686872 | 0.686872 | 0.0 | 0.000000 | 2.397399 | 0.000000 | 1.150286 | 2.017546 | 0.000000 | 7.574740 | 9.898919 | 0.686872 | 0.000000 | 0.0 | 0.686872 | 0.686872 | 9.306862 | 0.000000 | 1.782055 | 0.0 | 10.211670 | 0.0 | 0.0 | 0.000000 | 9.347942 | 2.017546 | 0.000000 | 1.782055 | 7.985573 | 0.000000 | 0.0 | 0.000000 | 0.0 | 0.0 | 8.713988 | 0.000000 | 0.0 | 0.686872 | 0.0 | 0.0 | 3.582099 | 2.219938 | 0.0 | 0.686872 | 0.000000 | 8.504679 | 0.000000 | 0.0 | 0.000000 | 7.961069 | 7.943307 | 0.0 | 0.0 | 11.641084 | 6.783355 | 8.111786 | 8.133878 | 0.0 | 0.0 | 9.748516 | 1.150286 | 0.000000 | 3.787179 | 0.000000 | 8.809238 | 1.782055 | 0.0 | 7.454503 | 10.444765 | 8.362235 | 0.0 | 2.017546 | 0.000000 | 0.686872 | 0.000000 | 5.508257 | 0.686872 | 7.615685 | 0.686872 | 0.000000 | 0.0 | 0.000000 | 0.000000 | 7.439376 | 0.0 | 0.0 | 0.0 | 0.0 | 0.0 | 2.827391 | 0.0 | 3.158217 | 0.0 | 2.219938 | 2.017546 | 0.0 | 0.000000 | 0.000000 | 0.686872 |
| HSPC\_037 | 0.0 | 0.0 | 0.0 | 1.869808 | 7.938080 | 0.000000 | 9.405107 | 0.0 | 0.000000 | 0.0 | 7.199831 | 9.495433 | 0.000000 | 0.0 | 0.000000 | 7.394504 | 3.164226 | 0.000000 | 8.086368 | 0.0 | 13.104234 | 9.083711 | 8.014129 | 0.0 | 0.000000 | 0.0 | 0.0 | 0.000000 | 8.418471 | 0.000000 | 0.0 | 2.657566 | 0.000000 | 8.337902 | 10.692584 | 0.000000 | 1.218731 | 8.500077 | 0.000000 | 0.0 | 8.308001 | 0.0 | 0.000000 | 7.275944 | 0.000000 | 0.000000 | 8.862937 | 0.000000 | 0.000000 | 1.218731 | 0.0 | 8.954567 | 2.316800 | 0.0 | 2.316800 | 11.106246 | 0.000000 | 0.0 | 8.510617 | 0.0 | 0.0 | 0.0 | 1.869808 | 5.397461 | 0.0 | 0.0 | 0.0 | 2.316800 | 0.0 | 0.000000 | 8.006701 | 9.250198 | 6.744074 | 1.869808 | 0.000000 | 0.0 | 10.085909 | 0.000000 | 0.0 | 1.218731 | 6.928313 | 0.0 | 0.0 | 0.0 | 0.000000 | 0.000000 | 1.218731 | 12.131326 | 5.568630 | 8.093396 | 1.218731 | 10.313640 | 2.657566 | 1.869808 | 1.218731 | 0.000000 | 7.682451 | 1.869808 | 1.218731 | 2.933024 | 0.000000 | 0.0 | 0.0 | 9.998750 | 7.772788 | 0.000000 | 0.0 | 1.218731 | 1.218731 | 8.148427 | 0.0 | 1.218731 | 0.000000 | 2.657566 | 1.218731 | 8.621744 | 7.815920 | 0.000000 | 0.0 | 0.0 | 0.0 | 0.0 | 4.637700 | 8.546913 | 2.316800 | 0.000000 | 3.538458 | 8.592276 | 1.218731 | 0.000000 | 0.000000 | 1.218731 | 0.0 | 7.615671 | 1.218731 | 7.514567 | 8.833854 | 2.316800 | 1.218731 | 2.657566 | 4.475001 | 6.943951 | 3.694521 | 0.0 | 0.0 | 1.218731 | 0.000000 | 8.664848 | 6.490844 | 7.482801 | 0.0 | 5.647156 | 1.218731 | 0.0 | 0.0 | 2.316800 | 1.218731 | 0.000000 | 0.000000 | 0.0 | 0.0 | 0.0 | 0.000000 | 10.392692 | 0.0 | 0.0 | 0.0 | 7.160213 | 0.000000 | 0.00000 | 0.000000 | 2.316800 | 1.218731 | 8.355550 | 0.000000 | 2.933024 | 0.000000 | 1.218731 | 0.0 | 0.000000 | 0.000000 | 8.669560 | 9.162687 | 9.873687 | 7.755167 | 1.218731 | 7.615671 | 1.218731 | 0.0 | 0.000000 | 8.626598 | 9.247050 | 2.316800 | 0.0 | 7.371559 | 6.779369 | 7.133183 | 1.869807 | 8.246273 | 0.000000 | 6.183499 | 7.348242 | 3.963623 | 8.808450 | 0.0 | 1.218731 | 1.218731 | 1.218731 | 1.218731 | 7.914462 | 4.190334 | 7.133183 | 0.000000 | 1.869807 | 6.813823 | 0.000000 | 0.000000 | 0.000000 | 1.218731 | 0.000000 | 9.162687 | 0.000000 | 6.689457 | 9.741228 | 7.605873 | 2.316800 | 1.218731 | 8.760682 | 3.363442 | 2.933024 | 0.0 | 8.636256 | 7.160213 | 1.869807 | 10.783395 | 8.057904 | 0.000000 | 0.000000 | 6.235258 | 3.164226 | 0.000000 | 0.000000 | 8.429623 | 3.694520 | 0.0 | 1.869807 | 0.000000 | 7.710151 | 1.218731 | 0.000000 | 0.000000 | 9.498083 | 0.000000 | 10.651504 | 10.660998 | 0.0 | 0.000000 | 0.0 | 0.0 | 7.482801 | 0.000000 | 0.000000 | 0.000000 | 0.000000 | 0.000000 | 0.000000 | 8.729440 | 1.218731 | 8.854687 | 1.218731 | 0.000000 | 5.303604 | 9.218403 | 1.869807 | 0.0 | 11.052258 | 1.218731 | 0.000000 | 0.000000 | 0.0 | 0.000000 | 3.164226 | 6.156907 | 0.0 | 7.755167 | 2.933024 | 0.000000 | 2.933024 | 3.164226 | 0.000000 | 9.700377 | 0.000000 | 0.000000 | 8.977543 | 8.577312 | 0.0 | 9.332705 | 1.869807 | 0.000000 | 0.00000 | 0.000000 | 0.0 | 3.835337 | 2.933024 | 1.218731 | 1.218731 | 0.0 | 1.218731 | 6.016053 | 7.348242 | 7.999236 | 8.367196 | 6.285223 | 8.531469 | 0.000000 | 10.283226 | 2.316800 | 7.930251 | 0.0 | 0.000000 | 9.087235 | 0.000000 | 0.0 | 0.0 | 9.492777 | 0.000000 | 1.869807 | 6.864006 | 2.657566 | 1.869807 | 5.303604 | 0.0 | 7.336441 | 7.450320 | 1.869807 | 4.190334 | 0.0 | 0.000000 | 0.000000 | 0.0 | 0.0 | 1.218731 | 9.111674 | 4.386210 | 1.869807 | 8.467986 | 7.849521 | 0.000000 | 9.859326 | 0.000000 | 0.0 | 7.953614 | 0.0 | 0.0 | 0.0 | 1.218731 | 0.000000 | 1.218731 | 0.000000 | 0.0 | 6.813823 | 7.428252 | 3.164226 | 0.0 | 0.000000 | 0.000000 | 2.316800 | 8.907487 | 3.164226 | 4.386210 | 10.124163 | 0.0 | 9.413556 | 3.164226 | 0.0 | 9.759018 | 0.000000 | 1.218731 | 0.0 | 0.000000 | 3.164226 | 0.000000 | 7.275944 | 0.0 | 7.238389 | 2.933024 | 0.000000 | 0.0 | 3.164226 | 0.000000 | 1.869807 | 0.0 | 4.978682 | 9.915931 | 0.0 | 7.173540 | 2.933024 | 0.000000 | 9.615036 | 0.000000 | 8.678935 | 7.874218 | 3.164226 | 7.383077 | 2.316800 | 1.869807 | 0.0 | 2.316800 | 0.0 | 3.363442 | 0.000000 | 9.080177 | 9.040723 | 0.0 | 0.000000 | 8.283626 | 0.0 | 0.000000 | 0.000000 | 1.218731 | 0.000000 | 0.0 | 0.0 | 0.000000 | 1.869807 | 0.000000 | 8.314031 | 0.0 | 0.000000 | 0.0 | 4.190334 | 0.000000 | 10.867803 | 1.218731 | 1.218731 | 8.014129 | 0.000000 | 9.506006 | 3.164226 | 7.472055 | 0.0 | 0.0 | 7.119476 | 3.164226 | 1.869807 | 0.0 | 2.316800 | 7.428252 | 0.000000 | 0.0 | 4.558643 | 0.0 | 1.218731 | 9.457801 | 2.316800 | 2.316800 | 2.316800 | 0.0 | 1.218731 | 2.933024 | 0.0 | 0.000000 | 0.000000 | 9.855197 | 7.968982 | 1.869807 | 2.933024 | 9.090753 | 8.577312 | 1.218731 | 8.148427 | 0.0 | 8.320036 | 9.018738 | 0.000000 | 0.000000 | 7.535363 | 0.000000 | 0.000000 | 7.849521 | 2.657566 | 5.150271 | 5.303604 | 0.0 | 11.012021 | 0.000000 | 0.0 | 0.0 | 2.933024 | 0.000000 | 0.000000 | 9.275141 | 1.869807 | 0.000000 | 7.644670 | 0.000000 | 8.289759 | 0.0 | 7.146761 | 0.000000 | 3.164226 | 0.0 | 0.0 | 8.331971 | 0.0 | 0.000000 | 10.386982 | 10.113830 | 6.55335 | 2.657566 | 1.218731 | 0.0 | 0.0 | 0.0 | 7.077557 | 0.0 | 4.916631 | 0.0 | 0.000000 | 10.516827 | 8.050700 | 8.395907 | 0.0 | 0.0 | 6.707893 | 1.218731 | 0.0 | 8.107351 | 0.0 | 7.700976 | 8.515859 | 10.286297 | 8.546913 | 10.304583 | 2.657566 | 0.000000 | 0.000000 | 2.933024 | 0.0 | 2.933024 | 2.316800 | 0.0 | 0.0 | 8.760682 | 1.218731 | 7.906503 | 3.164226 | 1.869807 | 8.833854 | 0.0 | 0.000000 | 0.0 | 1.869807 | 1.218731 | 8.484121 | 0.000000 | 0.000000 | 9.076634 | 8.372984 | 0.000000 | 6.285223 | 0.000000 | 0.0 | 0.0 | 8.484121 | 0.000000 | 1.218731 | 0.0 | 0.0 | 0.000000 | 3.363442 | 0.000000 | 0.00000 | 0.0 | 2.657566 | 0.000000 | 8.765091 | 10.404042 | 0.000000 | 7.566005 | 0.0 | 3.694520 | 8.923351 | 7.545649 | 0.0 | 0.000000 | 5.986154 | 8.467986 | 4.190334 | 0.000000 | 0.0 | 0.0 | 6.553350 | 0.0000 | 8.301946 | 7.635068 | 7.394504 | 7.348242 | 0.0 | 9.388059 | 0.000000 | 8.361384 | 1.218731 | 7.682451 | 8.641061 | 1.869807 | 8.636256 | 9.756806 | 6.309572 | 9.612592 | 0.0 | 9.883858 | 1.218731 | 7.615671 | 2.657566 | 15.812094 | 0.000000 | 0.0 | 6.074055 | 10.234780 | 0.0 | 7.359948 | 8.271282 | 7.312546 | 1.869807 | 0.000000 | 9.998750 | 0.0 | 7.857800 | 9.885883 | 0.000000 | 0.0 | 8.808450 | 0.0 | 2.316800 | 7.898499 | 0.000000 | 0.000000 | 0.0 | 0.000000 | 0.0 | 1.869807 | 1.218731 | 2.657566 | 0.0 | 0.000000 | 0.000000 | 0.0 | 10.679799 | 0.000000 | 9.476740 | 2.316800 | 4.291594 | 0.0 | 0.000000 | 1.218731 | 0.000000 | 1.218731 | 0.0 | 0.0 | 9.889926 | 0.0 | 1.218731 | 0.0 | 8.233604 | 0.000000 | 8.384491 | 0.0 | 0.000000 | 10.199386 | 7.824394 | 1.869807 | 5.038175 | 0.000000 | 6.896517 | 11.337700 | 0.000000 | 1.218731 | 2.933024 | 1.218731 | 0.000000 | 1.218731 | 0.000000 | 8.867044 | 9.933662 | 8.367196 | 5.527704 | 0.000000 | 7.359948 | 0.00000 | 0.0 | 8.246273 | 9.231205 | 1.218731 | 0.000000 | 8.557117 | 0.0 | 0.000000 | 7.048918 | 3.963623 | 6.129815 | 0.0 | 0.0 | 0.0 | 0.000000 | 9.427529 | 0.000000 | 7.857800 | 8.655380 | 10.146300 | 0.0 | 0.000000 | 0.000000 | 0.0 | 6.235258 | 10.552968 | 0.0 | 0.0 | 1.218731 | 0.000000 | 1.218731 | 8.014129 | 0.000000 | 4.783895 | 0.0 | 0.0 | 6.943952 | 6.726097 | 2.933024 | 4.291594 | 0.000000 | 3.164226 | 0.000000 | 8.582317 | 0.0 | 9.529518 | 0.000000 | 0.0 | 0.0 | 9.828064 | 1.218731 | 8.214389 | 3.363442 | 0.0 | 2.316800 | 9.215184 | 0.0 | 0.000000 | 1.218731 | 0.0 | 0.0 | 0.000000 | 0.000000 | 0.0 | 0.000000 | 1.218731 | 0.000000 | 1.869807 | 0.0 | 0.000000 | 6.912502 | 0.0 | 0.000000 | 0.0 | 2.316800 | 0.0 | 1.869807 | 3.363442 | 9.865499 | 5.859945 | 10.011792 | 8.181786 | 0.0 | 0.0 | 0.0 | 9.476740 | 0.0 | 0.0 | 7.225650 | 7.186745 | 1.218731 | 1.218731 | 0.0 | 0.0 | 0.000000 | 0.000000 | 1.869807 | 8.462568 | 3.538458 | 7.300448 | 0.0 | 9.935619 | 0.000000 | 1.869807 | 5.859945 | 0.0 | 3.538458 | 0.0 | 0.0 | 8.494778 | 10.042988 | 0.0 | 4.475001 | 0.0 | 0.0 | 3.363442 | 8.355550 | 0.000000 | 0.0 | 3.538458 | 8.557117 | 4.978682 | 0.0 | 0.0 | 0.0 | 0.000000 | 8.121174 | 1.218731 | 8.587306 | 9.111674 | 0.0 | 0.0 | 0.0 | 0.0 | 6.553350 | 0.000000 | 0.000000 | 6.813823 | 7.914462 | 8.175175 | 7.930251 | 0.000000 | 1.869807 | 0.0 | 0.0 | 0.0 | 0.000000 | 8.812716 | 12.473957 | 0.000000 | 5.757471 | 0.0 | 0.000000 | 1.218731 | 0.000000 | 9.293568 | 0.0 | 0.000000 | 5.351295 | 0.000000 | 6.796699 | 0.000000 | 2.933024 | 8.451670 | 1.869807 | 0.0 | 1.218731 | 0.0 | 11.369171 | 10.099937 | 7.186745 | 1.218731 | 0.000000 | 0.0 | 8.283626 | 0.000000 | 9.237565 | 0.0 | 2.316800 | 4.081427 | 0.0 | 0.000000 | 10.726889 | 7.336441 | 7.728326 | 0.000000 | 0.000000 | 0.0 | 0.0 | 3.835337 | 0.0 | 6.880354 | 0.0 | 0.0 | 0.000000 | 0.000000 | 7.781518 | 3.164226 | 1.218731 | 0.0 | 0.0 | 0.0 | 4.475001 | 8.473385 | 0.0 | 0.0 | 0.0 | 2.933024 | 7.737329 | 8.289759 | 9.379458 | 9.663070 | 1.869807 | 8.424058 | 7.719267 | 8.981337 | 0.0 | 9.076634 | 1.218731 | 6.333516 | 0.0 | 2.657566 | 0.0 | 3.694520 | 0.0 | 4.190334 | 9.094260 | 0.0 | 8.141662 | 0.000000 | 0.000000 | 8.155161 | 8.114280 | 7.790196 | 4.712649 | 0.000000 | 0.000000 | 1.218731 | 1.218731 | 9.991242 | 1.869807 | 2.933024 | 8.378749 | 0.0 | 9.149260 | 6.129815 | 1.218731 | 3.363442 | 8.181786 | 0.00000 | 5.150271 | 0.000000 | 1.869807 | 0.0 | 8.577312 | 8.331971 | 7.439328 | 0.000000 | 0.000000 | 7.394504 | 0.000000 | 9.379458 | 8.510617 | 1.218731 | 2.933024 | 2.316800 | 10.080613 | 0.0 | 9.895967 | 3.694520 | 1.218731 | 7.091666 | 0.000000 | 8.692885 | 0.0 | 6.490843 | 8.277468 | 0.000000 | 0.0 | 0.0 | 0.000000 | 8.706702 | 0.0 | 1.218731 | 8.833854 | 3.538458 | 0.0 | 8.923351 | 0.000000 | 8.271282 | 1.218731 | 8.747376 | 0.0 | 1.218731 | 7.324543 | 2.657566 | 3.363442 | 0.0 | 1.869807 | 0.000000 | 1.218731 | 1.869807 | 1.218731 | 1.869807 | 2.933024 | 8.825436 | 0.000000 | 0.000000 | 0.000000 | 8.724920 | 0.000000 | 2.316800 | 1.869807 | 11.461703 | 0.0 | 0.0 | 0.000000 | 0.000000 | 2.316800 | 6.403053 | 0.000000 | 1.869807 | 6.016053 | 1.218731 | 0.000000 | 0.0 | 1.218731 | 7.586077 | 8.036182 | 9.734499 | 0.000000 | 0.000000 | 0.0 | 1.218731 | 4.783895 | 1.869807 | 0.0 | 0.0 | 0.0 | 0.000000 | 0.0 | 7.405841 | 0.000000 | 9.115131 | 0.000000 | 8.911469 | 0.000000 | 7.874218 | 8.911469 | 2.657566 | 7.348242 | 0.0 | 6.045346 | 1.869807 | 10.078843 | 1.218731 | 1.218731 | 2.933024 | 0.000000 | 1.218731 | 0.0 | 0.000000 | 9.000159 | 7.417090 | 9.073083 | 7.383077 | 1.218731 | 0.0 | 2.316800 | 4.190334 | 9.083711 | 2.657566 | 1.218731 | 3.164226 | 10.033882 | 0.0 | 0.000000 | 2.657566 | 0.0 | 0.000000 | 8.418471 | 0.000000 | 1.869807 | 1.869807 | 7.832819 | 1.218731 | 9.101251 | 10.376936 | 0.000000 | 0.0 | 8.966101 | 5.924429 | 8.489459 | 0.000000 | 3.694520 | 0.0 | 10.911210 | 0.0 | 0.0 | 1.218731 | 9.804700 | 7.439328 | 1.218731 | 2.657566 | 1.218731 | 0.000000 | 0.0 | 1.218731 | 0.0 | 0.0 | 11.608145 | 0.000000 | 0.0 | 0.000000 | 0.0 | 0.0 | 6.309572 | 2.657566 | 0.0 | 0.000000 | 0.000000 | 4.081427 | 0.000000 | 0.0 | 0.000000 | 8.816968 | 6.309572 | 0.0 | 0.0 | 10.319646 | 5.485582 | 7.019698 | 8.155161 | 0.0 | 0.0 | 9.396608 | 2.657566 | 0.000000 | 9.122022 | 0.000000 | 1.218731 | 2.933024 | 0.0 | 0.000000 | 10.647927 | 8.931218 | 0.0 | 2.657566 | 0.000000 | 9.287452 | 0.000000 | 7.866032 | 3.363442 | 8.384491 | 8.114280 | 0.000000 | 0.0 | 8.320036 | 8.148427 | 7.615671 | 0.0 | 0.0 | 0.0 | 0.0 | 0.0 | 0.000000 | 0.0 | 7.336441 | 0.0 | 8.407233 | 1.218731 | 0.0 | 0.000000 | 1.218731 | 8.641061 |
| LT-HSC\_001 | 0.0 | 0.0 | 0.0 | 7.965715 | 5.885018 | 0.000000 | 8.962827 | 0.0 | 0.000000 | 0.0 | 2.364517 | 8.080749 | 0.000000 | 0.0 | 4.137438 | 3.749470 | 2.364517 | 0.000000 | 7.347331 | 0.0 | 13.944537 | 8.516418 | 3.749470 | 0.0 | 0.000000 | 0.0 | 0.0 | 0.000000 | 0.000000 | 0.000000 | 0.0 | 7.552600 | 0.000000 | 3.749470 | 9.893764 | 6.820748 | 2.364517 | 0.000000 | 0.000000 | 0.0 | 6.872760 | 0.0 | 0.000000 | 7.520353 | 0.000000 | 0.000000 | 2.364517 | 0.000000 | 0.000000 | 3.217169 | 0.0 | 7.232592 | 7.107933 | 0.0 | 8.564652 | 11.130502 | 0.000000 | 0.0 | 9.931038 | 0.0 | 0.0 | 0.0 | 0.000000 | 7.584142 | 0.0 | 0.0 | 0.0 | 8.397178 | 0.0 | 5.095857 | 8.414827 | 9.184807 | 3.217169 | 0.000000 | 2.364517 | 0.0 | 4.442877 | 0.000000 | 0.0 | 2.364517 | 0.000000 | 0.0 | 0.0 | 0.0 | 0.000000 | 0.000000 | 0.000000 | 13.092126 | 4.137438 | 3.217169 | 0.000000 | 9.639814 | 3.217169 | 8.187283 | 0.000000 | 0.000000 | 0.000000 | 3.749470 | 6.074609 | 2.364517 | 2.364517 | 0.0 | 0.0 | 8.742914 | 8.888979 | 0.000000 | 0.0 | 0.000000 | 8.227787 | 3.217169 | 0.0 | 2.364517 | 0.000000 | 8.685906 | 0.000000 | 7.271861 | 9.312700 | 8.914017 | 0.0 | 0.0 | 0.0 | 0.0 | 7.487369 | 4.909226 | 0.000000 | 0.000000 | 7.271861 | 2.364517 | 3.217169 | 5.982925 | 0.000000 | 8.548753 | 0.0 | 4.137438 | 2.364517 | 3.749470 | 0.000000 | 2.364517 | 0.000000 | 3.749470 | 7.645229 | 2.364517 | 3.749470 | 0.0 | 0.0 | 3.217169 | 7.917005 | 7.989468 | 7.674830 | 8.397178 | 0.0 | 2.364517 | 0.000000 | 0.0 | 0.0 | 8.207678 | 7.866592 | 0.000000 | 0.000000 | 0.0 | 0.0 | 0.0 | 0.000000 | 10.297362 | 0.0 | 0.0 | 0.0 | 7.584142 | 0.000000 | 5.77998 | 0.000000 | 8.267186 | 4.137438 | 2.364517 | 0.000000 | 2.364517 | 2.364517 | 0.000000 | 0.0 | 0.000000 | 0.000000 | 7.645229 | 3.217169 | 8.058464 | 7.107933 | 0.000000 | 7.232592 | 7.760152 | 0.0 | 8.267186 | 4.137438 | 9.967373 | 0.000000 | 0.0 | 8.187283 | 6.971475 | 8.926375 | 9.143059 | 7.063866 | 0.000000 | 3.749470 | 3.217169 | 12.461628 | 5.543737 | 0.0 | 0.000000 | 0.000000 | 0.000000 | 0.000000 | 9.110936 | 0.000000 | 3.217169 | 0.000000 | 10.136473 | 8.483344 | 0.000000 | 0.000000 | 2.364517 | 9.033078 | 0.000000 | 8.397178 | 0.000000 | 8.305537 | 8.466517 | 7.760152 | 9.522611 | 0.000000 | 7.760152 | 2.364517 | 2.364517 | 0.0 | 4.137438 | 8.058464 | 4.909226 | 3.749470 | 3.749470 | 0.000000 | 2.364517 | 8.700371 | 0.000000 | 0.000000 | 2.364517 | 8.080749 | 0.000000 | 0.0 | 8.532677 | 8.580378 | 7.674830 | 8.305537 | 0.000000 | 0.000000 | 4.137438 | 0.000000 | 7.645229 | 8.685906 | 0.0 | 0.000000 | 0.0 | 0.0 | 8.901552 | 2.364517 | 0.000000 | 2.364517 | 2.364517 | 0.000000 | 8.227787 | 5.885018 | 0.000000 | 3.217169 | 5.982925 | 7.645229 | 5.543737 | 9.705823 | 10.273376 | 0.0 | 9.438827 | 0.000000 | 0.000000 | 8.863498 | 0.0 | 0.000000 | 8.166596 | 4.694814 | 0.0 | 3.217169 | 3.217169 | 8.516418 | 9.464477 | 2.364517 | 3.217169 | 10.330287 | 2.364517 | 2.364517 | 0.000000 | 2.364517 | 0.0 | 10.442098 | 0.000000 | 7.107933 | 3.74947 | 5.885018 | 0.0 | 8.449492 | 2.364517 | 0.000000 | 0.000000 | 0.0 | 0.000000 | 4.442877 | 0.000000 | 7.192224 | 8.286489 | 3.217169 | 3.749470 | 0.000000 | 9.312700 | 7.150694 | 8.595934 | 0.0 | 0.000000 | 2.364517 | 2.364517 | 0.0 | 0.0 | 8.671295 | 0.000000 | 3.217169 | 8.058464 | 3.217169 | 2.364517 | 3.749470 | 0.0 | 8.166596 | 7.018409 | 7.150694 | 6.160812 | 0.0 | 0.000000 | 0.000000 | 0.0 | 0.0 | 2.364517 | 4.442877 | 2.364517 | 0.000000 | 2.364517 | 11.081615 | 4.137438 | 3.217169 | 3.749470 | 0.0 | 9.594069 | 0.0 | 0.0 | 0.0 | 2.364517 | 0.000000 | 2.364517 | 0.000000 | 0.0 | 2.364517 | 3.217169 | 0.000000 | 0.0 | 0.000000 | 0.000000 | 0.000000 | 8.532677 | 6.820749 | 9.979284 | 8.124310 | 0.0 | 6.461820 | 0.000000 | 0.0 | 5.261087 | 0.000000 | 8.305537 | 0.0 | 0.000000 | 4.694814 | 0.000000 | 9.522611 | 0.0 | 8.124310 | 7.703835 | 0.000000 | 0.0 | 0.000000 | 3.217169 | 3.217169 | 0.0 | 9.849024 | 7.674830 | 0.0 | 8.770595 | 6.652419 | 3.749470 | 2.364517 | 0.000000 | 4.442877 | 3.217169 | 0.000000 | 4.442877 | 9.455977 | 2.364517 | 0.0 | 3.749470 | 0.0 | 9.055756 | 0.000000 | 2.364517 | 9.264843 | 0.0 | 9.174482 | 9.530727 | 0.0 | 7.917005 | 0.000000 | 8.080749 | 3.217169 | 0.0 | 0.0 | 0.000000 | 8.286489 | 0.000000 | 3.217169 | 0.0 | 0.000000 | 0.0 | 0.000000 | 5.779980 | 8.227787 | 3.217169 | 0.000000 | 9.055756 | 0.000000 | 8.414827 | 0.000000 | 7.453613 | 0.0 | 0.0 | 3.749470 | 2.364517 | 0.000000 | 0.0 | 7.965715 | 6.971475 | 0.000000 | 0.0 | 0.000000 | 0.0 | 2.364517 | 9.078081 | 7.271861 | 2.364517 | 0.000000 | 0.0 | 8.837559 | 9.021605 | 0.0 | 0.000000 | 0.000000 | 7.941566 | 8.548753 | 8.286489 | 0.000000 | 0.000000 | 9.078081 | 0.000000 | 4.137438 | 0.0 | 7.347332 | 9.078081 | 0.000000 | 2.364517 | 0.000000 | 2.364517 | 0.000000 | 3.217169 | 3.217169 | 4.442877 | 2.364517 | 0.0 | 10.437787 | 8.483344 | 0.0 | 0.0 | 4.909226 | 0.000000 | 3.749470 | 8.449492 | 0.000000 | 0.000000 | 8.124310 | 2.364517 | 4.694814 | 0.0 | 3.217169 | 6.766791 | 7.941566 | 0.0 | 0.0 | 5.666688 | 0.0 | 0.000000 | 8.286489 | 8.863498 | 0.00000 | 3.217169 | 2.364517 | 0.0 | 0.0 | 0.0 | 8.305537 | 0.0 | 3.749470 | 0.0 | 0.000000 | 10.229170 | 4.137438 | 3.749470 | 0.0 | 0.0 | 8.187283 | 0.000000 | 0.0 | 7.674830 | 0.0 | 0.000000 | 9.055756 | 9.377142 | 7.787507 | 3.217169 | 3.217169 | 0.000000 | 2.364517 | 10.104202 | 0.0 | 0.000000 | 9.078081 | 0.0 | 0.0 | 9.153609 | 0.000000 | 3.749470 | 0.000000 | 0.000000 | 9.705823 | 0.0 | 0.000000 | 0.0 | 0.000000 | 0.000000 | 9.489678 | 0.000000 | 0.000000 | 3.217169 | 9.066961 | 0.000000 | 0.000000 | 0.000000 | 0.0 | 0.0 | 2.364517 | 0.000000 | 8.466517 | 0.0 | 0.0 | 0.000000 | 9.164084 | 0.000000 | 0.00000 | 0.0 | 3.217169 | 0.000000 | 4.694814 | 8.756821 | 0.000000 | 0.000000 | 0.0 | 3.749470 | 9.121723 | 3.749470 | 0.0 | 0.000000 | 9.184806 | 8.595934 | 6.591640 | 7.271861 | 0.0 | 0.0 | 0.000000 | 0.0000 | 6.591640 | 8.449492 | 0.000000 | 3.217169 | 0.0 | 4.137438 | 0.000000 | 8.548753 | 0.000000 | 3.217169 | 0.000000 | 0.000000 | 2.364517 | 9.789383 | 0.000000 | 4.137438 | 0.0 | 6.591640 | 8.641622 | 4.137438 | 0.000000 | 15.593200 | 0.000000 | 0.0 | 7.063866 | 9.421470 | 0.0 | 10.020216 | 5.261087 | 7.674830 | 0.000000 | 0.000000 | 7.760152 | 0.0 | 3.217169 | 7.584142 | 0.000000 | 0.0 | 5.885018 | 0.0 | 0.000000 | 8.548753 | 2.364517 | 2.364517 | 0.0 | 7.107933 | 0.0 | 7.018409 | 0.000000 | 0.000000 | 0.0 | 4.137438 | 0.000000 | 0.0 | 8.035830 | 3.749470 | 4.694814 | 6.710738 | 0.000000 | 0.0 | 8.499976 | 9.447428 | 5.666688 | 0.000000 | 0.0 | 0.0 | 9.594069 | 0.0 | 8.483344 | 0.0 | 9.340672 | 2.364517 | 0.000000 | 0.0 | 3.217169 | 10.219159 | 0.000000 | 3.217169 | 9.078081 | 0.000000 | 9.887456 | 8.342895 | 2.364517 | 8.876294 | 2.364517 | 9.368110 | 0.000000 | 0.000000 | 0.000000 | 5.666688 | 5.982925 | 2.364517 | 5.095857 | 0.000000 | 5.261087 | 0.00000 | 0.0 | 4.442877 | 3.217169 | 0.000000 | 0.000000 | 3.217169 | 0.0 | 0.000000 | 3.217169 | 3.217169 | 10.082279 | 0.0 | 0.0 | 0.0 | 0.000000 | 7.760152 | 0.000000 | 7.232592 | 3.749470 | 11.172608 | 0.0 | 0.000000 | 3.217169 | 0.0 | 6.591640 | 7.419049 | 0.0 | 0.0 | 8.267186 | 2.364517 | 0.000000 | 8.166596 | 0.000000 | 3.217169 | 0.0 | 0.0 | 10.115040 | 7.732268 | 4.442877 | 4.694814 | 8.124310 | 4.137438 | 0.000000 | 7.584142 | 0.0 | 8.714692 | 0.000000 | 0.0 | 0.0 | 9.912520 | 0.000000 | 5.095857 | 10.283019 | 0.0 | 10.093283 | 4.442877 | 0.0 | 4.909226 | 0.000000 | 0.0 | 0.0 | 2.364517 | 5.095857 | 0.0 | 2.364517 | 4.694814 | 0.000000 | 3.217169 | 0.0 | 0.000000 | 8.466517 | 0.0 | 0.000000 | 0.0 | 8.207678 | 0.0 | 0.000000 | 4.442877 | 11.771131 | 8.012835 | 10.802043 | 9.089116 | 0.0 | 0.0 | 0.0 | 11.133169 | 0.0 | 0.0 | 2.364517 | 6.461820 | 3.217169 | 0.000000 | 0.0 | 0.0 | 9.554808 | 2.364517 | 0.000000 | 4.442877 | 3.217169 | 5.543737 | 0.0 | 5.666688 | 6.591640 | 2.364517 | 0.000000 | 0.0 | 8.656534 | 0.0 | 0.0 | 3.217169 | 9.554808 | 0.0 | 8.532677 | 0.0 | 0.0 | 2.364517 | 6.710738 | 0.000000 | 0.0 | 2.364517 | 7.840709 | 3.217169 | 0.0 | 0.0 | 0.0 | 0.000000 | 8.611324 | 5.409323 | 6.710738 | 10.509359 | 0.0 | 0.0 | 0.0 | 0.0 | 4.442877 | 3.217169 | 0.000000 | 0.000000 | 5.261087 | 0.000000 | 8.714692 | 0.000000 | 0.000000 | 0.0 | 0.0 | 0.0 | 0.000000 | 8.342895 | 12.910433 | 0.000000 | 4.442877 | 0.0 | 7.107933 | 0.000000 | 2.364517 | 2.364517 | 0.0 | 2.364517 | 0.000000 | 0.000000 | 2.364517 | 0.000000 | 0.000000 | 3.217169 | 8.811145 | 0.0 | 0.000000 | 0.0 | 10.120428 | 6.710738 | 7.732268 | 0.000000 | 0.000000 | 0.0 | 4.694814 | 0.000000 | 10.788568 | 0.0 | 8.742914 | 6.971475 | 0.0 | 2.364517 | 4.909226 | 0.000000 | 9.768942 | 0.000000 | 0.000000 | 0.0 | 0.0 | 6.820749 | 0.0 | 0.000000 | 0.0 | 0.0 | 0.000000 | 6.319152 | 9.340672 | 8.626553 | 4.137438 | 0.0 | 0.0 | 0.0 | 8.124310 | 8.247621 | 0.0 | 0.0 | 0.0 | 0.000000 | 0.000000 | 10.002816 | 8.926375 | 5.982925 | 0.000000 | 3.749470 | 3.749470 | 9.669524 | 0.0 | 5.982925 | 0.000000 | 0.000000 | 0.0 | 0.000000 | 0.0 | 8.397178 | 0.0 | 4.137438 | 7.383635 | 0.0 | 0.000000 | 5.409323 | 0.000000 | 9.293747 | 8.626553 | 6.160812 | 0.000000 | 0.000000 | 4.694814 | 10.805393 | 6.528191 | 8.247621 | 9.205236 | 0.000000 | 3.217169 | 0.0 | 8.756821 | 0.000000 | 9.143059 | 2.364517 | 7.347332 | 0.00000 | 6.319152 | 0.000000 | 5.409323 | 0.0 | 9.768942 | 3.217169 | 3.217169 | 7.645229 | 2.364517 | 2.364517 | 0.000000 | 7.383635 | 6.319152 | 2.364517 | 6.392249 | 3.217169 | 7.150694 | 0.0 | 8.671295 | 6.766791 | 5.885018 | 0.000000 | 2.364517 | 7.107933 | 0.0 | 8.685906 | 8.012835 | 2.364517 | 0.0 | 0.0 | 0.000000 | 4.137438 | 0.0 | 2.364517 | 3.217169 | 7.453613 | 0.0 | 8.671295 | 0.000000 | 7.760152 | 6.160812 | 3.217169 | 0.0 | 8.499976 | 0.000000 | 7.520354 | 0.000000 | 0.0 | 0.000000 | 3.749470 | 7.814354 | 8.499976 | 0.000000 | 6.766791 | 9.205236 | 8.102695 | 2.364517 | 0.000000 | 0.000000 | 5.261087 | 9.089116 | 0.000000 | 3.217169 | 12.063208 | 0.0 | 0.0 | 0.000000 | 0.000000 | 8.938628 | 9.421470 | 0.000000 | 3.217169 | 0.000000 | 5.666688 | 9.918720 | 0.0 | 7.552600 | 3.217169 | 3.749470 | 7.674830 | 0.000000 | 2.364517 | 0.0 | 4.442877 | 4.442877 | 8.499976 | 0.0 | 0.0 | 0.0 | 0.000000 | 0.0 | 2.364517 | 0.000000 | 12.165795 | 0.000000 | 9.215343 | 0.000000 | 10.471911 | 5.982925 | 0.000000 | 9.322084 | 0.0 | 7.674830 | 0.000000 | 10.389508 | 2.364517 | 0.000000 | 9.601794 | 0.000000 | 0.000000 | 0.0 | 4.442877 | 10.104202 | 0.000000 | 8.361217 | 9.164084 | 0.000000 | 0.0 | 7.866592 | 8.700371 | 8.548753 | 9.143059 | 0.000000 | 0.000000 | 7.965715 | 0.0 | 0.000000 | 9.033078 | 0.0 | 0.000000 | 10.577548 | 0.000000 | 8.564652 | 2.364517 | 10.987443 | 0.000000 | 9.464477 | 8.824412 | 0.000000 | 0.0 | 0.000000 | 0.000000 | 9.802852 | 0.000000 | 4.137438 | 0.0 | 9.943251 | 0.0 | 0.0 | 0.000000 | 9.937158 | 2.364517 | 0.000000 | 8.324337 | 2.364517 | 0.000000 | 0.0 | 0.000000 | 0.0 | 0.0 | 10.420417 | 0.000000 | 0.0 | 2.364517 | 0.0 | 0.0 | 8.080749 | 2.364517 | 0.0 | 0.000000 | 8.145608 | 6.319152 | 0.000000 | 0.0 | 8.811145 | 9.132430 | 0.000000 | 0.0 | 0.0 | 10.173233 | 9.676858 | 7.989468 | 8.267186 | 0.0 | 0.0 | 10.812068 | 2.364517 | 2.364517 | 9.264843 | 7.552600 | 8.580378 | 2.364517 | 0.0 | 8.166596 | 10.933363 | 7.732268 | 0.0 | 0.000000 | 0.000000 | 2.364517 | 0.000000 | 8.166596 | 8.770595 | 2.364517 | 0.000000 | 2.364517 | 0.0 | 0.000000 | 9.121723 | 9.481327 | 0.0 | 0.0 | 0.0 | 0.0 | 0.0 | 0.000000 | 0.0 | 0.000000 | 0.0 | 3.749470 | 6.820749 | 0.0 | 7.107933 | 2.364517 | 6.242153 |
| HSPC\_001 | 0.0 | 0.0 | 0.0 | 8.395500 | 0.000000 | 0.377367 | 0.676211 | 0.0 | 0.676211 | 0.0 | 0.000000 | 8.923709 | 0.377367 | 0.0 | 0.000000 | 8.851780 | 9.012407 | 7.123321 | 7.640356 | 0.0 | 12.746870 | 9.232729 | 9.914646 | 0.0 | 0.000000 | 0.0 | 0.0 | 5.069857 | 0.000000 | 0.000000 | 0.0 | 1.628905 | 0.000000 | 9.412471 | 11.118833 | 0.000000 | 0.000000 | 0.676211 | 0.377367 | 0.0 | 6.802432 | 0.0 | 0.000000 | 4.414804 | 0.377367 | 7.107768 | 8.010188 | 5.867946 | 0.000000 | 9.303379 | 0.0 | 8.824449 | 1.134796 | 0.0 | 1.482235 | 8.987981 | 0.000000 | 0.0 | 1.318950 | 0.0 | 0.0 | 0.0 | 0.377367 | 0.000000 | 0.0 | 0.0 | 0.0 | 0.377367 | 0.0 | 0.000000 | 1.482235 | 7.239191 | 8.617097 | 0.000000 | 0.000000 | 0.0 | 2.921687 | 0.000000 | 0.0 | 1.762030 | 0.000000 | 0.0 | 0.0 | 0.0 | 0.000000 | 0.000000 | 0.000000 | 12.396286 | 0.000000 | 0.676211 | 0.000000 | 11.169149 | 0.377367 | 9.699115 | 0.000000 | 0.000000 | 0.377367 | 0.000000 | 0.000000 | 8.285068 | 0.000000 | 0.0 | 0.0 | 11.201561 | 0.923649 | 0.377367 | 0.0 | 0.676211 | 5.216270 | 9.912410 | 0.0 | 8.639983 | 0.000000 | 2.374501 | 7.993360 | 0.000000 | 7.224846 | 0.676211 | 0.0 | 0.0 | 0.0 | 0.0 | 7.224846 | 9.392715 | 0.000000 | 0.000000 | 9.060862 | 1.628905 | 0.923649 | 0.000000 | 0.000000 | 4.434885 | 0.0 | 8.771148 | 1.628905 | 10.008081 | 8.286449 | 8.276747 | 8.061140 | 1.762030 | 0.000000 | 8.663575 | 4.586200 | 0.0 | 0.0 | 8.923709 | 0.000000 | 7.925790 | 1.482235 | 9.740544 | 0.0 | 8.240127 | 0.000000 | 0.0 | 0.0 | 1.883902 | 8.577000 | 0.000000 | 0.000000 | 0.0 | 0.0 | 0.0 | 7.520972 | 9.818564 | 0.0 | 0.0 | 0.0 | 0.000000 | 0.377367 | 0.00000 | 0.000000 | 0.377367 | 9.422563 | 0.377367 | 0.000000 | 8.668884 | 0.377367 | 0.000000 | 0.0 | 5.918739 | 0.000000 | 4.265819 | 0.000000 | 9.042166 | 0.923649 | 0.000000 | 3.519027 | 8.720921 | 0.0 | 0.000000 | 8.950983 | 8.410789 | 0.676211 | 0.0 | 0.000000 | 0.676211 | 8.584884 | 6.136699 | 7.233470 | 1.628905 | 0.377367 | 1.996276 | 2.288830 | 9.442536 | 0.0 | 0.000000 | 0.000000 | 0.000000 | 0.000000 | 6.111968 | 0.000000 | 2.604657 | 0.000000 | 10.683373 | 8.965724 | 3.853357 | 0.000000 | 0.377367 | 0.000000 | 0.000000 | 1.482235 | 0.000000 | 0.000000 | 9.960822 | 7.587515 | 1.318950 | 0.000000 | 9.564895 | 7.530335 | 1.628905 | 0.0 | 8.609387 | 8.121239 | 0.676211 | 8.514683 | 1.762030 | 0.377367 | 0.000000 | 0.000000 | 1.628905 | 0.377367 | 0.000000 | 0.000000 | 2.288830 | 0.0 | 1.628905 | 1.134796 | 1.134796 | 0.000000 | 0.000000 | 0.377367 | 10.135483 | 0.676211 | 1.318950 | 8.785881 | 0.0 | 0.000000 | 0.0 | 0.0 | 7.854899 | 0.377367 | 0.000000 | 7.663942 | 0.000000 | 0.377367 | 0.000000 | 5.670382 | 0.000000 | 7.499681 | 0.000000 | 0.000000 | 9.520952 | 9.448104 | 0.000000 | 0.0 | 8.703439 | 1.318950 | 6.563421 | 0.377367 | 0.0 | 0.000000 | 8.443370 | 1.318950 | 0.0 | 9.606986 | 6.723001 | 0.377367 | 8.671001 | 0.377367 | 0.000000 | 10.844519 | 0.000000 | 0.000000 | 0.676211 | 1.883902 | 0.0 | 10.464271 | 3.882894 | 0.000000 | 0.00000 | 0.000000 | 0.0 | 8.356553 | 1.482235 | 0.000000 | 7.281391 | 0.0 | 0.000000 | 4.173225 | 8.508774 | 0.923649 | 7.330454 | 0.000000 | 9.775414 | 0.377367 | 8.996450 | 0.377367 | 9.137149 | 0.0 | 0.000000 | 0.000000 | 0.000000 | 0.0 | 0.0 | 9.482276 | 7.088883 | 0.377367 | 1.318950 | 8.542711 | 7.809477 | 1.482235 | 0.0 | 9.920888 | 3.968042 | 1.318950 | 9.261827 | 0.0 | 0.000000 | 0.000000 | 0.0 | 0.0 | 9.772458 | 6.786892 | 0.676211 | 0.676211 | 5.030808 | 11.404691 | 0.000000 | 7.758600 | 7.079347 | 0.0 | 7.278616 | 0.0 | 0.0 | 0.0 | 6.625896 | 0.000000 | 0.000000 | 0.000000 | 0.0 | 9.288282 | 8.392936 | 9.193486 | 0.0 | 0.377367 | 0.000000 | 0.000000 | 0.923649 | 9.289661 | 10.363761 | 7.273049 | 0.0 | 4.099659 | 1.134796 | 0.0 | 2.977514 | 0.000000 | 9.935939 | 0.0 | 0.000000 | 1.996276 | 0.000000 | 4.493504 | 0.0 | 1.134796 | 9.102259 | 3.592379 | 0.0 | 0.923649 | 0.000000 | 7.607558 | 0.0 | 0.377367 | 0.377367 | 0.0 | 0.000000 | 3.995344 | 7.902545 | 9.643577 | 0.377367 | 0.923649 | 8.056288 | 0.000000 | 1.883902 | 1.628905 | 1.628905 | 0.0 | 8.057907 | 0.0 | 9.902980 | 0.676211 | 1.482235 | 8.315172 | 0.0 | 0.000000 | 8.858300 | 0.0 | 0.000000 | 0.000000 | 3.083081 | 0.000000 | 0.0 | 0.0 | 0.000000 | 2.921687 | 0.000000 | 9.107741 | 0.0 | 0.377367 | 0.0 | 0.000000 | 6.608321 | 10.655834 | 1.628905 | 0.000000 | 9.932853 | 0.000000 | 8.069193 | 0.676211 | 8.487300 | 0.0 | 0.0 | 8.833932 | 1.628905 | 5.509264 | 0.0 | 2.604657 | 1.883902 | 8.511140 | 0.0 | 7.281391 | 0.0 | 0.000000 | 0.377367 | 9.534391 | 0.676211 | 1.318950 | 0.0 | 0.676211 | 7.709989 | 0.0 | 0.000000 | 6.074058 | 0.923649 | 6.970060 | 0.676211 | 1.628905 | 1.134796 | 3.519027 | 0.000000 | 0.676211 | 0.0 | 8.524086 | 8.845230 | 0.377367 | 0.000000 | 0.000000 | 0.000000 | 0.000000 | 4.394439 | 0.000000 | 9.883934 | 0.000000 | 0.0 | 10.435905 | 0.000000 | 0.0 | 0.0 | 9.766528 | 0.377367 | 9.098330 | 6.086805 | 0.000000 | 0.000000 | 8.816817 | 0.000000 | 7.525660 | 0.0 | 8.147335 | 0.377367 | 3.401495 | 0.0 | 0.0 | 6.237319 | 0.0 | 0.000000 | 1.134796 | 1.482235 | 0.00000 | 8.566798 | 0.000000 | 0.0 | 0.0 | 0.0 | 1.134796 | 0.0 | 9.515068 | 0.0 | 0.000000 | 9.761568 | 8.943118 | 1.628905 | 0.0 | 0.0 | 9.188320 | 7.544266 | 0.0 | 0.676211 | 0.0 | 5.974679 | 8.515861 | 9.857676 | 9.503825 | 3.882894 | 8.969171 | 0.000000 | 0.000000 | 1.134796 | 0.0 | 0.676211 | 0.676211 | 0.0 | 0.0 | 5.082642 | 7.000696 | 0.000000 | 0.923649 | 0.676211 | 1.482235 | 0.0 | 0.000000 | 0.0 | 0.377367 | 0.000000 | 9.410571 | 0.000000 | 0.000000 | 8.086751 | 2.531943 | 0.000000 | 0.000000 | 0.000000 | 0.0 | 0.0 | 8.571341 | 0.000000 | 0.377367 | 0.0 | 0.0 | 0.000000 | 0.676211 | 0.000000 | 6.38397 | 0.0 | 3.031264 | 5.004176 | 6.759286 | 10.834868 | 0.000000 | 7.183903 | 0.0 | 10.077985 | 8.673117 | 8.596073 | 0.0 | 0.377367 | 0.676211 | 1.628905 | 1.628905 | 8.208397 | 0.0 | 0.0 | 0.000000 | 0.0000 | 0.923649 | 0.676211 | 0.000000 | 4.787026 | 0.0 | 8.127421 | 0.377367 | 9.011572 | 7.007417 | 1.482235 | 0.676211 | 0.377367 | 0.000000 | 0.377367 | 0.377367 | 0.676211 | 0.0 | 1.482235 | 0.377367 | 8.144289 | 6.763263 | 14.466537 | 0.000000 | 0.0 | 0.377367 | 6.021909 | 0.0 | 0.676211 | 8.618195 | 9.168968 | 8.661447 | 0.000000 | 8.059525 | 0.0 | 1.996276 | 1.996276 | 0.000000 | 0.0 | 8.620389 | 0.0 | 0.000000 | 9.508570 | 0.000000 | 1.482235 | 0.0 | 0.000000 | 0.0 | 1.134796 | 0.000000 | 0.000000 | 0.0 | 7.676647 | 0.000000 | 0.0 | 8.396780 | 0.000000 | 2.100526 | 9.437569 | 0.676211 | 0.0 | 0.000000 | 0.000000 | 0.000000 | 8.639983 | 0.0 | 0.0 | 9.758583 | 0.0 | 5.600985 | 0.0 | 9.989108 | 0.000000 | 1.318950 | 0.0 | 0.000000 | 9.627852 | 0.676211 | 7.553480 | 0.000000 | 0.000000 | 7.478070 | 9.471989 | 1.628905 | 0.377367 | 8.708603 | 0.676211 | 0.000000 | 0.000000 | 0.377367 | 8.396780 | 8.817774 | 0.377367 | 0.000000 | 0.000000 | 1.883902 | 4.83315 | 0.0 | 0.000000 | 8.618195 | 0.000000 | 0.000000 | 1.762030 | 0.0 | 0.000000 | 2.977514 | 3.031264 | 8.331331 | 0.0 | 0.0 | 0.0 | 0.000000 | 10.336666 | 0.000000 | 0.377367 | 1.134796 | 9.692875 | 0.0 | 5.069857 | 0.000000 | 0.0 | 1.482235 | 8.778042 | 0.0 | 0.0 | 0.000000 | 0.923649 | 3.853357 | 8.359182 | 0.000000 | 8.839592 | 0.0 | 0.0 | 0.923649 | 0.000000 | 4.690105 | 8.598301 | 0.000000 | 1.318950 | 0.377367 | 7.408690 | 0.0 | 8.307024 | 0.000000 | 0.0 | 0.0 | 11.226616 | 0.000000 | 0.377367 | 8.840533 | 0.0 | 8.171473 | 8.984579 | 0.0 | 0.000000 | 0.000000 | 0.0 | 0.0 | 0.000000 | 5.953955 | 0.0 | 0.000000 | 0.000000 | 7.377903 | 0.923649 | 0.0 | 0.000000 | 7.458585 | 0.0 | 0.000000 | 0.0 | 8.116584 | 0.0 | 8.717852 | 9.644115 | 11.752090 | 0.000000 | 10.211298 | 9.051950 | 0.0 | 0.0 | 0.0 | 9.945156 | 0.0 | 0.0 | 9.002350 | 9.150871 | 2.531943 | 0.377367 | 0.0 | 0.0 | 0.377367 | 0.377367 | 0.000000 | 2.921687 | 7.820968 | 9.959956 | 0.0 | 9.118646 | 0.377367 | 8.493298 | 0.000000 | 0.0 | 0.923649 | 0.0 | 0.0 | 1.628905 | 2.197748 | 0.0 | 9.216145 | 0.0 | 0.0 | 3.519027 | 1.134796 | 0.000000 | 0.0 | 6.167028 | 10.550200 | 7.941667 | 0.0 | 0.0 | 0.0 | 0.000000 | 5.537397 | 0.000000 | 0.676211 | 8.250077 | 0.0 | 0.0 | 0.0 | 0.0 | 0.377367 | 0.000000 | 0.000000 | 0.000000 | 1.482235 | 0.377367 | 9.303379 | 0.000000 | 0.377367 | 0.0 | 0.0 | 0.0 | 0.000000 | 4.604046 | 10.497768 | 0.000000 | 2.531943 | 0.0 | 0.000000 | 0.000000 | 0.000000 | 0.000000 | 0.0 | 0.000000 | 0.000000 | 0.000000 | 5.867946 | 0.000000 | 0.923649 | 1.762030 | 4.099659 | 0.0 | 0.000000 | 0.0 | 10.043252 | 1.134796 | 1.762030 | 0.000000 | 0.000000 | 0.0 | 0.377367 | 0.000000 | 7.636025 | 0.0 | 1.134796 | 9.846475 | 0.0 | 0.676211 | 9.338449 | 0.000000 | 0.377367 | 0.377367 | 0.000000 | 0.0 | 0.0 | 9.600889 | 0.0 | 8.113473 | 0.0 | 0.0 | 0.000000 | 0.377367 | 10.449541 | 2.673882 | 9.999258 | 0.0 | 0.0 | 0.0 | 0.676211 | 0.000000 | 0.0 | 0.0 | 0.0 | 0.377367 | 0.000000 | 10.079579 | 9.070521 | 10.222171 | 8.300199 | 0.676211 | 8.450785 | 10.698247 | 0.0 | 7.622956 | 0.923649 | 0.000000 | 0.0 | 0.676211 | 0.0 | 8.228669 | 0.0 | 8.283684 | 9.610853 | 0.0 | 0.000000 | 0.000000 | 2.604657 | 9.476233 | 6.599451 | 0.923649 | 0.000000 | 0.000000 | 0.000000 | 0.000000 | 8.156434 | 9.612506 | 0.377367 | 0.000000 | 0.000000 | 0.0 | 7.873411 | 0.000000 | 0.000000 | 1.628905 | 6.414630 | 5.52808 | 8.885003 | 0.676211 | 0.676211 | 0.0 | 7.242044 | 1.318950 | 2.921687 | 0.000000 | 0.377367 | 0.000000 | 0.377367 | 10.542702 | 8.477653 | 0.000000 | 0.923649 | 8.533429 | 2.288830 | 0.0 | 9.603663 | 9.580752 | 0.000000 | 0.000000 | 5.370192 | 5.712118 | 0.0 | 1.482235 | 1.628905 | 0.000000 | 0.0 | 0.0 | 0.676211 | 1.134796 | 0.0 | 0.923649 | 7.362259 | 9.059246 | 0.0 | 0.377367 | 0.000000 | 8.791733 | 5.661887 | 8.810106 | 0.0 | 0.000000 | 0.000000 | 1.482235 | 0.377367 | 0.0 | 0.000000 | 0.000000 | 0.676211 | 0.676211 | 0.000000 | 8.979461 | 7.441317 | 0.923649 | 0.000000 | 0.000000 | 0.377367 | 8.317878 | 0.676211 | 1.318950 | 8.222906 | 12.126537 | 0.0 | 0.0 | 0.000000 | 0.000000 | 0.676211 | 0.676211 | 0.377367 | 0.676211 | 0.000000 | 0.377367 | 0.000000 | 0.0 | 8.644301 | 8.425918 | 0.377367 | 0.000000 | 0.000000 | 0.000000 | 0.0 | 2.739936 | 8.959672 | 0.377367 | 0.0 | 0.0 | 0.0 | 0.000000 | 0.0 | 0.000000 | 0.377367 | 1.883902 | 0.000000 | 10.312110 | 0.000000 | 4.124600 | 8.799498 | 1.134796 | 3.360089 | 0.0 | 7.558065 | 2.374501 | 10.136633 | 1.482235 | 0.000000 | 7.551182 | 0.000000 | 7.451210 | 0.0 | 0.676211 | 10.394527 | 8.633481 | 0.377367 | 9.589737 | 0.000000 | 0.0 | 0.000000 | 7.974620 | 9.727375 | 9.550012 | 0.000000 | 9.245576 | 9.297222 | 0.0 | 0.000000 | 8.703439 | 0.0 | 0.377367 | 8.125878 | 0.377367 | 0.000000 | 1.318950 | 0.676211 | 0.676211 | 6.237319 | 6.833019 | 0.377367 | 0.0 | 7.043829 | 0.000000 | 8.814903 | 0.000000 | 8.390367 | 0.0 | 8.732122 | 0.0 | 0.0 | 8.673117 | 9.525641 | 0.923649 | 0.000000 | 10.337334 | 3.853357 | 0.000000 | 0.0 | 0.676211 | 0.0 | 0.0 | 11.414046 | 0.377367 | 0.0 | 0.923649 | 0.0 | 0.0 | 1.628905 | 0.676211 | 0.0 | 0.000000 | 0.000000 | 8.230106 | 0.000000 | 0.0 | 0.000000 | 9.630032 | 0.000000 | 0.0 | 0.0 | 10.754757 | 8.291965 | 7.555774 | 9.070521 | 0.0 | 0.0 | 9.177187 | 1.762030 | 0.000000 | 8.746254 | 0.377367 | 7.629506 | 1.134796 | 0.0 | 0.000000 | 10.477341 | 10.039568 | 0.0 | 1.318950 | 0.377367 | 6.563421 | 0.377367 | 9.440054 | 1.134796 | 1.134796 | 7.707927 | 0.000000 | 0.0 | 0.377367 | 5.359726 | 9.581316 | 0.0 | 0.0 | 0.0 | 0.0 | 0.0 | 0.000000 | 0.0 | 0.377367 | 0.0 | 1.134796 | 1.134796 | 0.0 | 1.883902 | 0.000000 | 1.318950 |

In [9]:

```
%time scbool = scBoolSeq().fit(ref_data)
scbool
```

```
Computing bimodality index for 475/1165 genes
Computing bimodality index for 757/1165 genes
CPU times: user 1min 9s, sys: 1.87 s, total: 1min 11s
Wall time: 11.8 s
```

Out[9]:

```
scBoolSeqBinarizer()
```

**In a Jupyter environment, please rerun this cell to show the HTML representation or trust the notebook.   
On GitHub, the HTML representation is unable to render, please try loading this page with nbviewer.org.**

scBoolSeqBinarizer

```
scBoolSeqBinarizer()
```

## Generate synthetic RNA-Seq data¶

In [10]:

```
SEED = 1928327465
n_samples = 200
synth_pseudocounts = scbool.sample_counts(random_walk_df, n_samples_per_state=n_samples, random_state=SEED)
```

In [11]:

```
n_samples = 300 # number of samples per row
```

In [12]:

```
scbool.sample_counts?
```

In [13]:

```
counts = scbool.sample_counts(random_walk_df, n_samples_per_state=n_samples)
counts.head()
```

Out[13]:

|  | tf | gene1 | gene2 | gene3 | gene4 | gene5 | gene6 | gene7 | gene8 | gene9 | gene10 |
| --- | --- | --- | --- | --- | --- | --- | --- | --- | --- | --- | --- |
| 0 | 9.977497 | 7.735747 | 3.234694 | 2.471814 | 1.349850 | 0.000000 | 2.573179 | 0.000000 | 14.542216 | 0.000000 | 0.000000 |
| 1 | 9.804195 | 9.291098 | 0.000000 | 2.640593 | 0.634105 | 0.000000 | 2.730283 | 0.000000 | 15.698360 | 2.426172 | 0.239030 |
| 2 | 9.569535 | 9.874674 | 1.732880 | 2.625558 | 3.046362 | 0.000000 | 2.233210 | 0.896598 | 16.005468 | 2.356798 | 0.208952 |
| 3 | 10.718736 | 9.959593 | 3.998083 | 12.781793 | 2.259282 | 0.137870 | 3.909543 | 2.476530 | 15.361846 | 2.154460 | 0.000000 |
| 4 | 10.459761 | 9.718845 | 2.988492 | 5.866417 | 0.000000 | 0.041811 | 7.974766 | 0.000000 | 15.878582 | 3.547310 | 0.000000 |

To ease post-analysis with STREAM, we generate unique identifiers for each simulated row (cell):

In [14]:

```
ids = [f"step{x}_{y}"  for y in range(n_samples) for x in random_walk_df.index]
counts.index = ids
counts.index.name = "cellID"
counts
```

Out[14]:

|  | tf | gene1 | gene2 | gene3 | gene4 | gene5 | gene6 | gene7 | gene8 | gene9 | gene10 |
| --- | --- | --- | --- | --- | --- | --- | --- | --- | --- | --- | --- |
| cellID |  |  |  |  |  |  |  |  |  |  |  |
| step0\_0 | 9.977497 | 7.735747 | 3.234694 | 2.471814 | 1.349850 | 0.000000 | 2.573179 | 0.000000 | 14.542216 | 0.000000 | 0.000000 |
| step1\_0 | 9.804195 | 9.291098 | 0.000000 | 2.640593 | 0.634105 | 0.000000 | 2.730283 | 0.000000 | 15.698360 | 2.426172 | 0.239030 |
| step2\_0 | 9.569535 | 9.874674 | 1.732880 | 2.625558 | 3.046362 | 0.000000 | 2.233210 | 0.896598 | 16.005468 | 2.356798 | 0.208952 |
| step3\_0 | 10.718736 | 9.959593 | 3.998083 | 12.781793 | 2.259282 | 0.137870 | 3.909543 | 2.476530 | 15.361846 | 2.154460 | 0.000000 |
| step4\_0 | 10.459761 | 9.718845 | 2.988492 | 5.866417 | 0.000000 | 0.041811 | 7.974766 | 0.000000 | 15.878582 | 3.547310 | 0.000000 |
| ... | ... | ... | ... | ... | ... | ... | ... | ... | ... | ... | ... |
| step6\_299 | 10.161107 | 10.702292 | 0.810351 | 9.580091 | 0.065626 | 0.000000 | 10.125693 | 0.000000 | 15.961203 | 6.716956 | 9.138774 |
| step7\_299 | 9.736781 | 10.238058 | 4.463780 | 10.437671 | 8.922433 | 0.000000 | 6.999750 | 0.000000 | 16.563433 | 9.820661 | 8.632838 |
| step8\_299 | 10.018816 | 9.449022 | 12.816000 | 9.407083 | 6.404636 | 0.000000 | 9.852068 | 0.000000 | 15.497264 | 8.976746 | 9.376482 |
| step9\_299 | 10.230816 | 9.839100 | 9.119868 | 8.456540 | 10.268140 | 0.000000 | 9.272988 | 7.548856 | 15.352179 | 8.357286 | 8.079808 |
| step10\_299 | 10.545584 | 14.181191 | 11.633391 | 9.678786 | 9.767101 | 0.423314 | 6.832149 | 7.861243 | 16.230053 | 10.747740 | 9.779068 |

3300 rows × 11 columns

In [15]:

```
%time synth_scbool = scBoolSeq().fit(counts)
```

```
Computing bimodality index for 7/11 genes
Computing bimodality index for 7/11 genes
CPU times: user 2.21 s, sys: 1.11 s, total: 3.32 s
Wall time: 359 ms
```

In [16]:

```
synth_scbool.criteria_.Category.value_counts()
```

Out[16]:

```
Category
Bimodal     7
Unimodal    3
ZeroInf     1
Name: count, dtype: int64
```

In [17]:

```
synth_scbool.criteria_.plot.scatter(x="Mean", y="Variance")
```

Out[17]:

```
<Axes: xlabel='Mean', ylabel='Variance'>
```

In [18]:

```
synth_scbool.criteria_.plot.scatter(x="Mean", y="DropOutRate")
```

Out[18]:

```
<Axes: xlabel='Mean', ylabel='DropOutRate'>
```

In [19]:

```
from sklearn.decomposition import PCA
from sklearn.manifold import TSNE
from sklearn.pipeline import Pipeline, FunctionTransformer
from sklearn.manifold import LocallyLinearEmbedding, SpectralEmbedding
```

In [20]:

```
def rename_columns(df):
    """ """
    df.columns = [f"dim{i}" for i in range(df.shape[1])]
    return df

vis_pipeline = Pipeline([
    ('pca', PCA()),
    ('subset_pca', FunctionTransformer(lambda x: x.iloc[:, :4])),
    ('lle', LocallyLinearEmbedding(n_neighbors=300, n_components=2, random_state=SEED)),
    ('rename_cols', FunctionTransformer(rename_columns))
])
```

In [21]:

```
%time _pre_vis = vis_pipeline.fit_transform(synth_pseudocounts)
```

```
CPU times: user 21.5 s, sys: 59.1 s, total: 1min 20s
Wall time: 5.51 s
```

In [22]:

```
random_walk_df
```

Out[22]:

|  | tf | gene1 | gene2 | gene3 | gene4 | gene5 | gene6 | gene7 | gene8 | gene9 | gene10 |
| --- | --- | --- | --- | --- | --- | --- | --- | --- | --- | --- | --- |
| 0 | 1 | 0 | 0 | 0 | 0 | 0 | 0 | 0 | 0 | 0 | 0 |
| 1 | 1 | 0 | 0 | 0 | 0 | 0 | 0 | 0 | 1 | 0 | 0 |
| 2 | 1 | 1 | 0 | 0 | 0 | 0 | 0 | 0 | 1 | 0 | 0 |
| 3 | 1 | 1 | 0 | 1 | 0 | 0 | 0 | 0 | 1 | 0 | 0 |
| 4 | 1 | 1 | 0 | 1 | 0 | 0 | 1 | 0 | 1 | 0 | 0 |
| 5 | 1 | 1 | 0 | 1 | 0 | 0 | 1 | 0 | 1 | 1 | 0 |
| 6 | 1 | 1 | 0 | 1 | 0 | 0 | 1 | 0 | 1 | 1 | 1 |
| 7 | 1 | 1 | 0 | 1 | 1 | 0 | 1 | 0 | 1 | 1 | 1 |
| 8 | 1 | 1 | 1 | 1 | 1 | 0 | 1 | 0 | 1 | 1 | 1 |
| 9 | 1 | 1 | 1 | 1 | 1 | 0 | 1 | 1 | 1 | 1 | 1 |
| 10 | 1 | 1 | 1 | 1 | 1 | 1 | 1 | 1 | 1 | 1 | 1 |

In [23]:

```
import matplotlib as mpl
def colorFader(c1, c2, mix=0):
    """fade (linear interpolate) from color c1 (at mix=0) to c2 (mix=1)
    taken from:
    https://stackoverflow.com/questions/25668828/how-to-create-colour-gradient-in-python
    """
    c1 = np.array(mpl.colors.to_rgb(c1))
    c2 = np.array(mpl.colors.to_rgb(c2))
    return mpl.colors.to_hex((1 - mix) * c1 + mix * c2)
```

In [24]:

```
n_active_genes = random_walk_df.sum(axis=1)
n_active_genes.name = "n_active_genes"
vis_frame = _pre_vis.join(n_active_genes)
vis_frame.index.name = "index"
vis_frame = vis_frame.reset_index().drop("index", axis="columns")
vis_frame.head()
```

Out[24]:

|  | dim0 | dim1 | n\_active\_genes |
| --- | --- | --- | --- |
| 0 | -0.022211 | -0.019980 | 1 |
| 1 | -0.026448 | -0.009560 | 1 |
| 2 | -0.020374 | -0.018226 | 1 |
| 3 | -0.025110 | -0.021894 | 1 |
| 4 | -0.023226 | -0.013966 | 1 |

In [25]:

```
levels = vis_frame.n_active_genes
norm_levels = levels / levels.max()
print(norm_levels.shape)
norm_levels.unique()
```

```
(2200,)
```

Out[25]:

```
array([0.09090909, 0.18181818, 0.27272727, 0.36363636, 0.45454545,
       0.54545455, 0.63636364, 0.72727273, 0.81818182, 0.90909091,
       1.        ])
```

In [26]:

```
color = norm_levels.apply(lambda x: colorFader("darkblue", "lightgreen", x))
color.name = "color"
vis_frame = vis_frame.join(color)
vis_frame
```

Out[26]:

|  | dim0 | dim1 | n\_active\_genes | color |
| --- | --- | --- | --- | --- |
| 0 | -0.022211 | -0.019980 | 1 | #0d168b |
| 1 | -0.026448 | -0.009560 | 1 | #0d168b |
| 2 | -0.020374 | -0.018226 | 1 | #0d168b |
| 3 | -0.025110 | -0.021894 | 1 | #0d168b |
| 4 | -0.023226 | -0.013966 | 1 | #0d168b |
| ... | ... | ... | ... | ... |
| 2195 | 0.025303 | -0.033900 | 11 | #90ee90 |
| 2196 | 0.026706 | -0.021673 | 11 | #90ee90 |
| 2197 | 0.027313 | -0.007632 | 11 | #90ee90 |
| 2198 | 0.023092 | -0.024051 | 11 | #90ee90 |
| 2199 | 0.027196 | -0.024065 | 11 | #90ee90 |

2200 rows × 4 columns

In [27]:

```
ax = None
for label, frame in vis_frame.groupby('n_active_genes'):
    scatter_kwargs = dict(x='dim0', y='dim1', label=label, c=frame['color'], alpha=.8)
    if ax is not None:
        scatter_kwargs.update({'ax': ax})
    ax = frame.plot.scatter(**scatter_kwargs)
```

In [ ]:

```

```
