# Supplementary material for: scBoolSeq: Linking scRNA-seq statistics and Boolean dynamics
Source: PLoS Comput Biol. 2024 Jul 8;20(7):e1011620. doi: 10.1371/journal.pcbi.1011620 (PMC11257695; doi:10.1371/journal.pcbi.1011620)
Supplement: S1 Fig — Left: Distribution of rate parameters λ estimated on dataset GSE122466. Right: Dropout probabilities computed between the minimum and maximum values of a sample from the parametric distributions corresponding to the same dataset. Each line corresponds to an individual gene. (PDF) [file pcbi.1011620.s002.pdf]

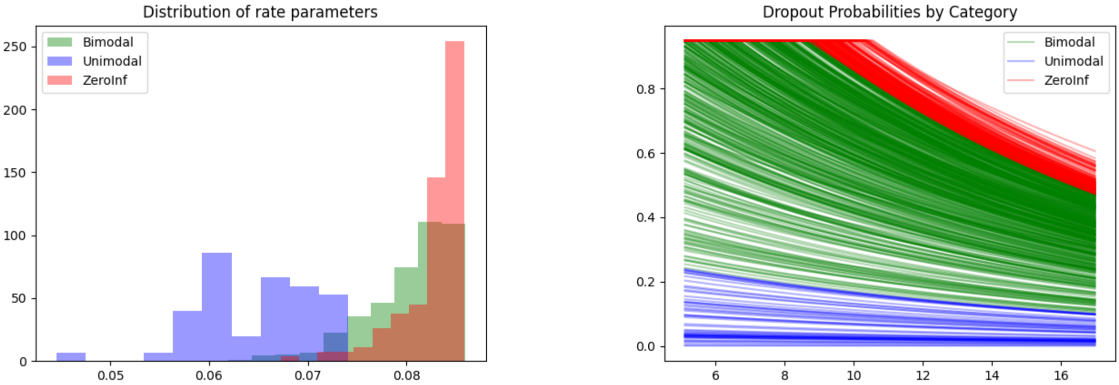

S1 Fig. Example of distribution of rate parameters and dropout probabilities learnt by scBoolSeq. Left: Distribution of rate parameters λ estimated on dataset GSE122466. **Right**: Dropout probabilities computed between the minimum and maximum values of a sample from the parametric distributions corresponding to the same dataset. Each line corresponds to an individual gene.
