# Supplementary material for: scBoolSeq: Linking scRNA-seq statistics and Boolean dynamics
Source: PLoS Comput Biol. 2024 Jul 8;20(7):e1011620. doi: 10.1371/journal.pcbi.1011620 (PMC11257695; doi:10.1371/journal.pcbi.1011620)
Supplement: S2 Fig — Each green point represents the average of 100 independent replicates with the same sample size as the reference dataset. (PDF) [file pcbi.1011620.s003.pdf]

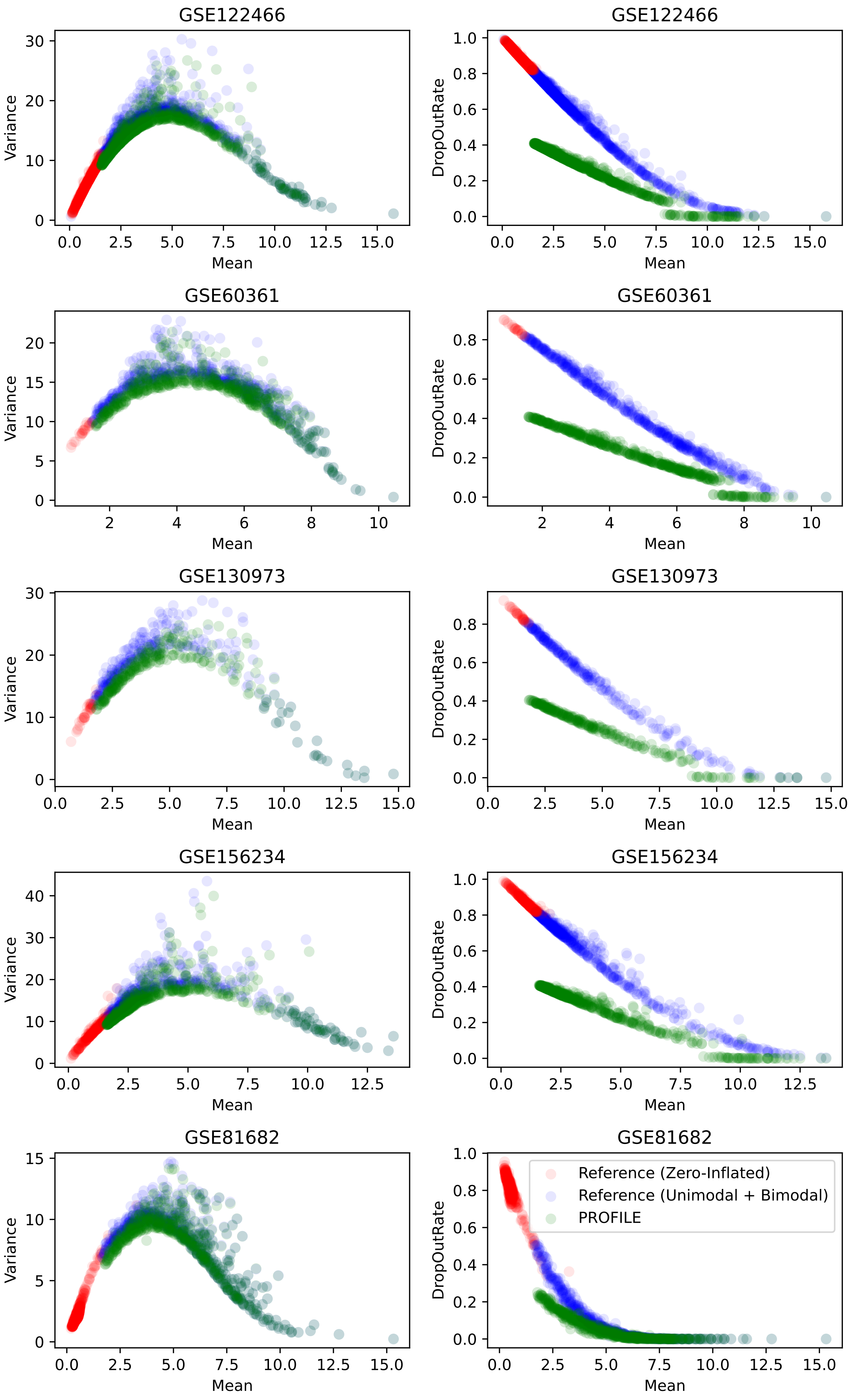

S2 Fig. Mean - Variance and Mean - DropOutRate relationships of HVGs using PROFILE parametric distibutions for bimodal and unimodel genes on selected scRNA-Seq datasets. Each green point represents the average of 100 independent replicates with the same sample size as the reference dataset.
