# Supplementary material for: scBoolSeq: Linking scRNA-seq statistics and Boolean dynamics
Source: PLoS Comput Biol. 2024 Jul 8;20(7):e1011620. doi: 10.1371/journal.pcbi.1011620 (PMC11257695; doi:10.1371/journal.pcbi.1011620)
Supplement: S3 Fig — (PDF) [file pcbi.1011620.s004.pdf]

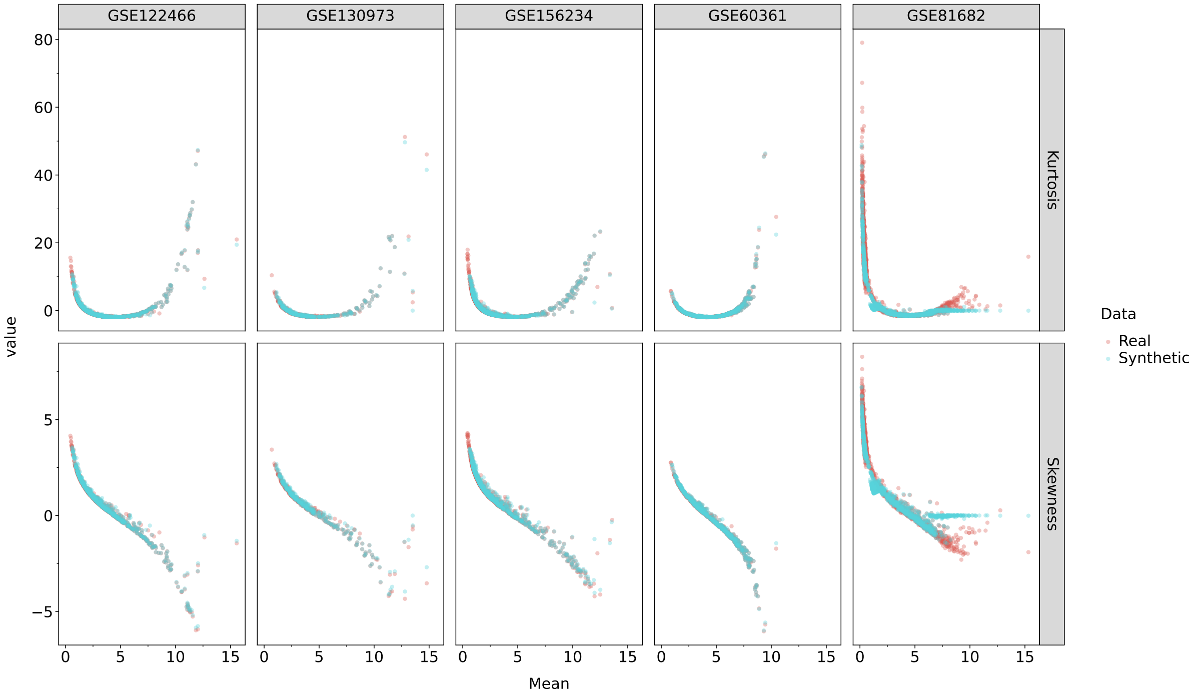

S3 Fig. Correlation between higher moments of real pseudocount data and from data generated from distributions and dropout model learnt by scBoolSeq on selected scRNA-Seq datasets
