# Supplementary material for: scBoolSeq: Linking scRNA-seq statistics and Boolean dynamics
Source: PLoS Comput Biol. 2024 Jul 8;20(7):e1011620. doi: 10.1371/journal.pcbi.1011620 (PMC11257695; doi:10.1371/journal.pcbi.1011620)
Supplement: S4 Fig — t-SNE and UMAP projections trained on the top 25 principal components (log pseudocount matrix). Colours indicate cell identities determined by binary value of known markers (see Table 1). (PDF) [file pcbi.1011620.s005.pdf]

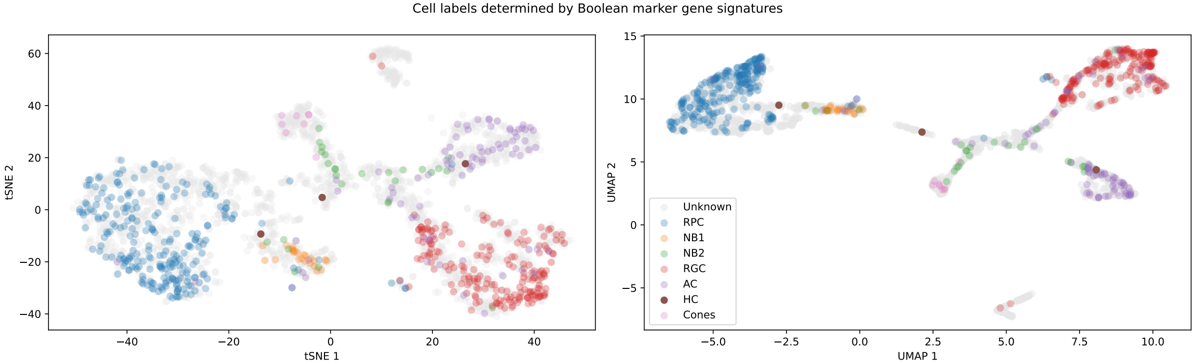

S4 Fig. Position of cells classified using scBoolSeq binarisation and prior-knowledge markers. t-SNE and UMAP projections trained on the top 25 principal components (log pseudocount matrix). Colours indicate cell identities determined by binary value of known markers (see Table 1 of main text).
