# Supplementary material for: scBoolSeq: Linking scRNA-seq statistics and Boolean dynamics
Source: PLoS Comput Biol. 2024 Jul 8;20(7):e1011620. doi: 10.1371/journal.pcbi.1011620 (PMC11257695; doi:10.1371/journal.pcbi.1011620)
Supplement: S5 Fig — UMAP projection of the first 25 principal components to 3 dimensions (only 2 are shown). The cluster labels are determined by the majority label of unambiguous cell types identified via scBoolSeq binarisation. (PDF) [file pcbi.1011620.s006.pdf]

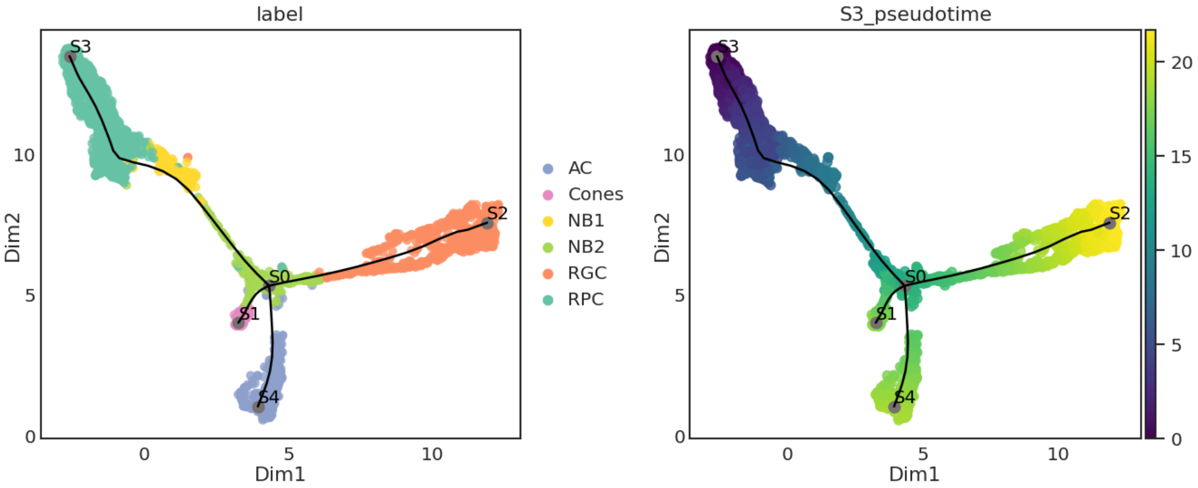

S5 Fig. Result of trajectory reconstruction using STREAM on early-born retinal neurons scRNA-Seq data. UMAP projection of the first 25 principal components to 3 dimensions (only 2 are shown). The cluster labels are determined by the majority label of unambiguous cell types identified via scBoolSeq binarisation.
