# Supplementary material for: scBoolSeq: Linking scRNA-seq statistics and Boolean dynamics
Source: PLoS Comput Biol. 2024 Jul 8;20(7):e1011620. doi: 10.1371/journal.pcbi.1011620 (PMC11257695; doi:10.1371/journal.pcbi.1011620)
Supplement: S6 Fig — This graph comprises 184 nodes forming Boolean networks that can reproduce the Boolean dynamics of early-born retinal neurons differentiation process. This graph is a subgraph of the input DoRothEA TF-TF interaction database. Green arrows indicate positive regulations, red arrows indicate negative regulations. Nodes without predecessors indicate nodes with constant function in the Boolean networks. Thus, the Boolean state of these nodes is identical in all stable states, and is in opposite state in the precursor state RPC. (PDF) [file pcbi.1011620.s007.pdf]

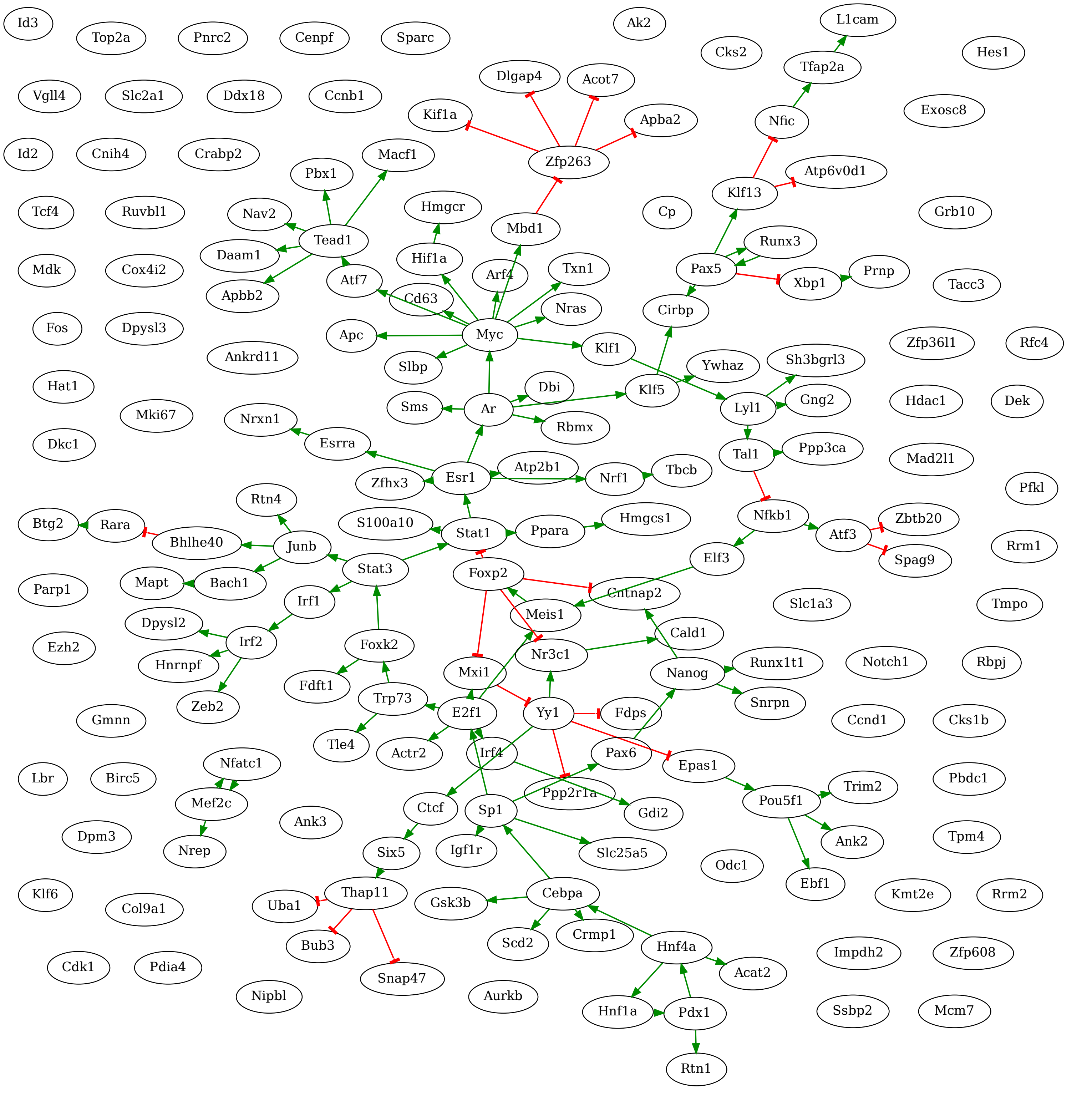

S6 Fig. Influence graph of sparser Boolean networks learnt using BoNesis from qualitative dynamics of case study obtained with scBoolSeq binarisation. This graph comprises 184 nodes forming Boolean networks that can reproduce the Boolean dynamics of early-born retinal neurons differentiation process. This graph is a subgraph of the input DoRothEA TF-TF interaction database. Green arrows indicate positive regulations, red arrows indicate negative regulations. Nodes without predecessors indicate nodes with constant function in the Boolean networks. Thus, the Boolean state of these nodes is identical in all stable states, and is in opposite state in the precursor state RPC.
