# Supplementary material for: scBoolSeq: Linking scRNA-seq statistics and Boolean dynamics
Source: PLoS Comput Biol. 2024 Jul 8;20(7):e1011620. doi: 10.1371/journal.pcbi.1011620 (PMC11257695; doi:10.1371/journal.pcbi.1011620)
Supplement: S7 Fig — See S1 Notebooks for full pipeline. (PDF) [file pcbi.1011620.s008.pdf]

```python
# Domain of possible Boolean networks from input GRN
pkn = bonesis.domains.InfluenceGraph(
    pkn_biggest_weakly_connected_component, maxclause=8, allow_skipping_nodes=True, canonic=False
)
# BoNesis setup
bo = bonesis.BoNesis(pkn, binarised_data)
# trajectory from RPC to NB1 and then to NB2
~bo.obs("RPC") >= ~bo.obs("NB1") >= ~bo.obs("NB2")
# trajectory from NB2 to stable state Cones
~bo.obs("NB2") >= bo.fixed(~bo.obs("Cones"))
# trajectory from NB2 to stable state RGC
~bo.obs("NB2") >= bo.fixed(~bo.obs("RGC"))
# trajectory from NB2 to stable state AC
~bo.obs("NB2") >= bo.fixed(~bo.obs("AC"))

# explain dynamics of as much as genes as possible
bo.maximize_nodes()
# rely on the state changes of as less as genes as possible
bo.maximize_strong_constants()
# give access to the genes whose dynamics is necessary to obtain the dynamical properties
view = bonesis.NonStrongConstantNodesView(bo, mode="optN")
```

S7 Fig. Python code snippet showing usage of BoNesis for the inference of Boolean networks for the retinal differentiation case study. See S1 Code/3. - Retinal Differentiation BN Inference for full pipeline.
