# Supplementary material for: scBoolSeq: Linking scRNA-seq statistics and Boolean dynamics
Source: PLoS Comput Biol. 2024 Jul 8;20(7):e1011620. doi: 10.1371/journal.pcbi.1011620 (PMC11257695; doi:10.1371/journal.pcbi.1011620)
Supplement: S8 Fig — Genes classified as Unimodal may exhibit heavy tails or remain skewed after the preprocessing log-transformation step. However, these characteristics do not hinder their coarse-graining: scBoolSeq uses a nonparametric quantile-based binarisation scheme that makes no assumptions about the underlying distribution. This is independent from the biased sampling procedure: By using half-normal distributions, scBoolSeq produces synthetic data reflecting unimodal activation patterns found in Boolean gene dynamics. (PDF) [file pcbi.1011620.s009.pdf]

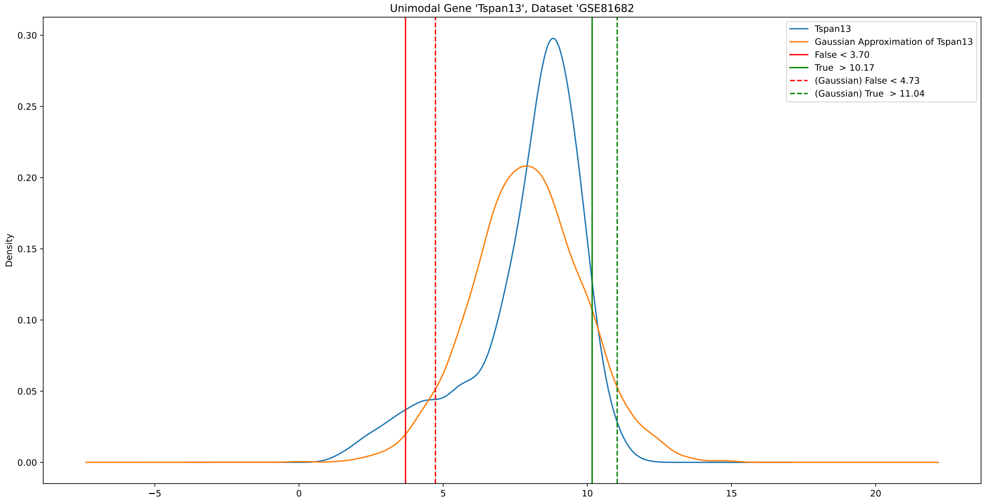

S8 Fig. Disambiguation of Unimodal genes' coarse-graining and sampling parametrisations Genes classified as Unimodal may exhibit heavy tails or remain skewed after the preprocessing log-transformation step. However, these characteristics do not hinder their coarse-graining: scBoolSeq uses a nonparametric quantile-based binarisation scheme which makes no assumptions about the underlying distribution. This is independent from the biased sampling step: By using half-normal distributions, scBoolSeq produces synthetic data reflecting unimodal activation patterns found in Boolean gene dynamics.
