# Supplementary material for: scBoolSeq: Linking scRNA-seq statistics and Boolean dynamics
Source: PLoS Comput Biol. 2024 Jul 8;20(7):e1011620. doi: 10.1371/journal.pcbi.1011620 (PMC11257695; doi:10.1371/journal.pcbi.1011620)
Supplement: S9 Fig — A Gaussian distribution (for Unimodal Genes) or two-component Gaussian Mixture (for Bimodal Genes) by themselves do not suffice to capture the statistical characteristics of log-transformed and normalised Highly Variable Genes of scRNA-seq datasets. However, when combined with our probabilistic dropout model these parametric distributions are able to recover the statistics of these data. (PDF) [file pcbi.1011620.s010.pdf]

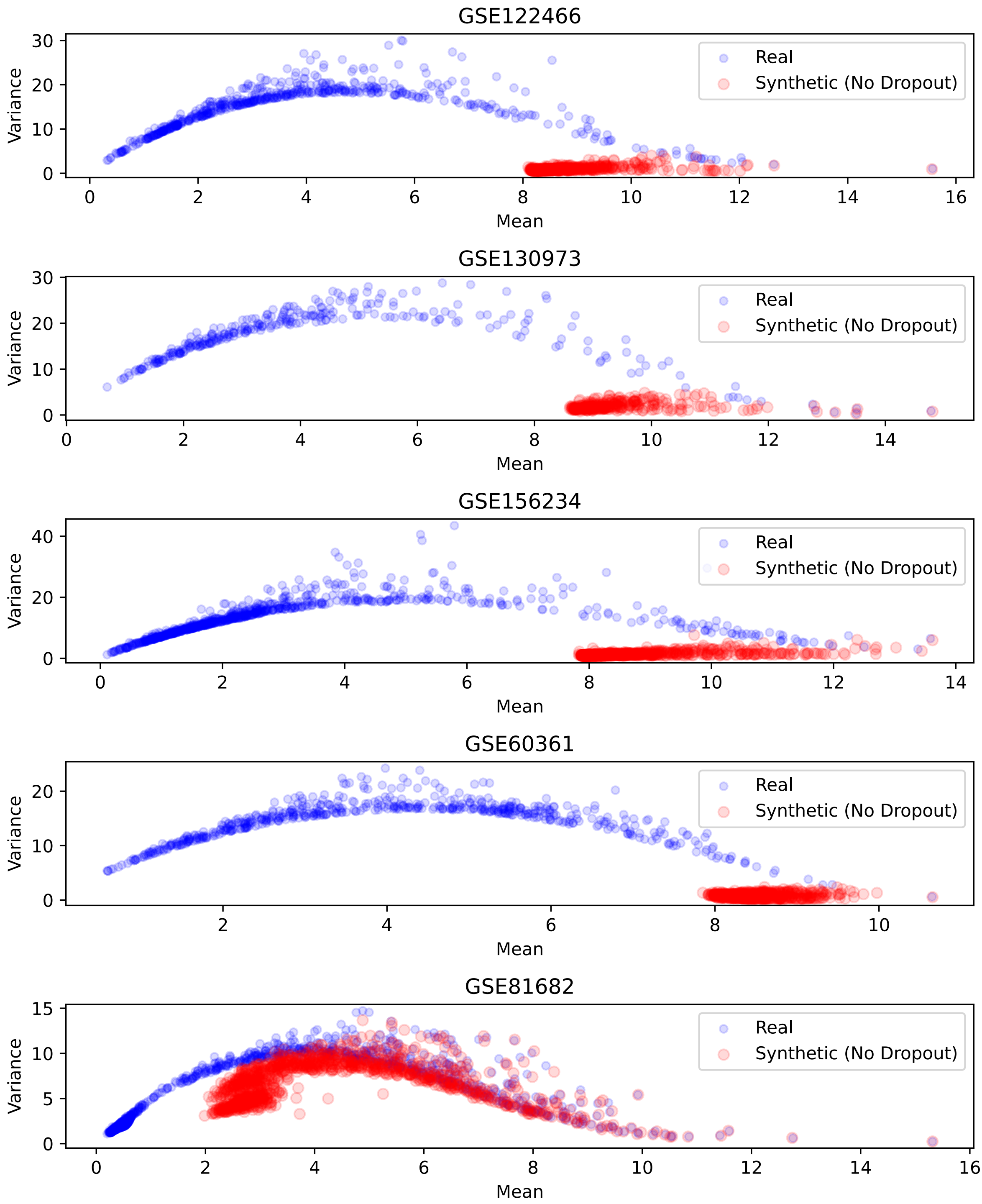

S9 Fig. Importance of scBoolSeq's Dropout model A Gaussian distribution (for Unimodal Genes) or two-component Gaussian Mixture (for Bimodal Genes) by themselves do not suffice to capture the statistical characteristics of log-transformed and normalised Highly Variable Genes of scRNA-seq datasets. As shown in the main text, these parametric distributions combined with our probabilistic dropout model are able to recover the statistics of these data.
