# Supplementary material for: scBoolSeq: Linking scRNA-seq statistics and Boolean dynamics
Source: PLoS Comput Biol. 2024 Jul 8;20(7):e1011620. doi: 10.1371/journal.pcbi.1011620 (PMC11257695; doi:10.1371/journal.pcbi.1011620)
Supplement: Attachment — Submitted filename: reply_to_reviews.pdf [file pcbi.1011620.s011.pdf]

We thank the reviewers for carefully evaluating our manuscript and for their suggestions which significantly improved the manuscript's readability. Furthermore, their valuable feedback allowed us to better elaborate on the preprocessing and validation that should be performed on scRNA-seq data prior to the usage of scBoolSeq.

Please find below a point by point response to reviewers comments.

Reviewer #1

Authors propose a novel method for binarizing scRNAseq data to generate a Boolean representation of experimental data. These approaches are crucial for enhancing dynamic modeling of genetic regulatory networks by formalizing data representation. They compare their method to another, presenting advantages. The techniques appear sound, and I believe the work is valuable and merits publication after addressing some queries and making necessary revisions.

Questions:

- Is the classification of scRNAseq data into only three categories sufficiently general?

We employed the following validation scheme: Comparing the moments of gene-wise distributions sampled from our model to those computed on experimental data. Rather than just checking each moment's distribution individually, we explored pairwise scatter plots in order to verify that our model correctly captured the correlation structure that characterises these data. In the 5 studied datasets, we observed that moments seem very well preserved, as well as the pairwise correlations between them. This suggests that the three categories are sufficient to capture the main statistical features of the data, at least for the purpose of classification in Boolean activation states.

We extended the Validation section with more details on the validation methodology and the studied datasets. We also added the comparison when ignoring the dropout model, which leads to drastically different statistics for the generated data.

Nevertheless, future work could consider exploring other classification schemes, perhaps applied to other transformations or raw count data.

- The "zeroinf" case appears to be the most noisy. Are the "?" cases discarded? It would be beneficial to observe cases that do not fit into these three categories and how the authors handle them.

Albeit genes classified as "zeroinf" may appear to be noisy, the zero-or-not coarse-graining scheme has proven to be effective, arguing that zeros can be interpreted as a meaningful biological insight as detailed in *Qiu P.*

Embracing the dropouts in single-cell RNA-seq analysis. *Nature Communications. 2020;11(1):1–9. doi:10.1038/s41467-020-14976-9.* We recognise that this is a modelling choice that must be made by the end-user analysing the data. The binarisation, descirbed in Eq. (8) this parametrisation.

In general, a gene which has not been discarded in the training step cannot be classified as "?" accross all the cells.

We added further explanation on the procedure to discard genes in the "Classification of Pseudocount Distributions and Dropout Model" section, and further details on the binarisation procedure in the "Binarisation of scRNA-seq data" section.

- In the second paragraph of the section 'From pseudocounts to...': Can the authors devise a method in scBoolseq to reject a dataset if it is inadequate for binarization? Otherwise, users may employ it in inappropriate cases.

We added a section on the data prepocessing that must be performed on the dataset in order to ensure that our method can be meaningful. This notably involves the selection of highly variable genes (HVG). Indeed, in order to binarise the data, we assume that we have enough observations of the genes in both states. Our hypothesis is that HVG possess this property. All other genes should be discarded for the application of scBoolSeq. The identification of HVGs is out of the scope of this paper. We hope that his section addressses the reviewer's concern.

- I was able to install the software without problems, however I did not test it with data. A tutorial on using scBoolSeq would be highly beneficial.

We thank the reviewer for this suggestion. We added tutorals notebooks for both complementary operations, which complement the already provided "Basic scBoolSeq usage" with preprocessing steps. These showcase the integration of scBoolSeq with the popular scRNA-seq data analysis package scanpy.

- Can an inferred Boolean network be exported from the software to be used in another Boolean tool?

Yes, the Boolean networks returned by the software BoNesis can be exported to standard textual format (BoolNet). We added a note about that in the Material and Methods.

Minor:
Line 38: "active"
Line 57: Please define 'GRN'.
Line 86: "HVGs"
Line 355: Provide a reference for 'k-nearest-neighbors classifier'.
Line 356: Provide a reference for 'by-category bijective matching'.

We applied the suggested corections

Please cite the link 'github.com/colomoto/colomoto-docker' for scBoolSeq, as other Colomoto webpages in the web appear to be outdated.

We used official links to the CoLoMoTo Docker, which are up-to-date.

Reviewer #2

In this manuscript, López et al. present scBoolSeq, a method to link scRNA-seq data with Boolean network models by learning gene expression distributions to binarize data or simulate realistic data from Boolean states.

The method addresses an important need and the manuscript is generally well-written. However, the below points should be addressed to strengthen the work. In particular, further evaluation of the binarization results and discussion of computing requirements would help users assess the method's applicability. With these issues addressed, the work will be a valuable contribution to the field.

Major points:

1) How does the binarization model treat housekeeping genes that are constitutively active? By looking at the unimodal example in Figure 3, it seems that these genes will have 3 states instead of being in the 1 state.

We thank the reviewer for this comment as the prerequisite for selecting Highly Variable Genes (HVGs) and applying scBoolSeq exclusively on them rather than on the whole dataset was not clear and explicit in the main text.

We have added a subsection at the beginning of the Results section devoted to the prerequisites on data processing. In particular, we explicitely mention that housekeeping genes are not supposed to be binarised. Indeed, our binarisation methods require that the gene have sufficient observations both in low/off and high/on states. We provided a reference that explains how the HVGs selection step filters out housekeeping genes.

2) How do you treat unimodal genes that are not symmetric? Symmetric thresholds may be suboptimal.

We thank the reviewer for raising this important point. The thresholds are not symmetric, they are based on the empirical distributions' quantiles. We meant that the *margin quantiles* used to calculate the binarisation thresholds are symmetric q, $1 - q$. As explained in the revised version of the text, the binarisation scheme for unimodal genes does not require their distributions to be symmetric.

3) Regarding the statement on lines 120-121, "The log transformation is necessary in order to ensure the validity of the underlying parametric distributions", please consider briefly expanding on this.

We added a full paragraph explaining this statement in the new "Prerequisites on data preprocessing" section.

4) Lines 116-117 state "...would represent the standard library size normalisation, yielding counts/reads per million (CPM/RPM)." This is not correct; a multiplicative factor of 1E6 is needed.

Indeed. We corrected the statement accordingly.

5) The text states, "In a first step, genes which do not exhibit a high enough variability". Please define "high enough" quantitatively.

In the section "Classification of Pseudocount Distributions and Dropout Model", we detailed how the selection is performed and how the variability is measured. We rely on thresholds on the dropout rate and dynamic range, by comparing the amplitude of gene pseudocounts with median of amplitude accross all genes.

6) Regarding the statement on lines 411-412, "Future engineering work will focus on leveraging the AnnData [59] Python package for handling large datasets that cannot be fit in RAM," please expand on the computing requirements and maximum dataset size that can be analyzed.

The current implementation of scBoolSeq (relying on `pandas` library) requires loading the full pseudocount matrices in memory. This consistutes a bottleneck for handling very large datasets, which could be circumvented by using more advanced data structures.

However, usual scRNA-seq datasets over a few thousands of cells ten thoushand of genes can be handled with classical hardware setup. For example, For example, the case study on early born retinal neurons used a typical 10X dataset comprising 5347 measurements (single-cells) across 15176 genes and required about 660 MB of memory. We added this precision in the Validation section.

Minor points:
It is better to spell "scRNA-Seq" as "scRNA-seq" throughout the manuscript.
On line 127, fix the typo: "patters" -¿ "patterns".

We applied the suggested corrections.
